# The cotranscriptional folding landscape for two cyclic di-nucleotide-sensing riboswitches with highly homologous aptamer domains acting either as ON- or OFF-switches

**DOI:** 10.1093/nar/gkac514

**Published:** 2022-06-23

**Authors:** Tom Landgraf, Albrecht Eduard Völklein, Boris Fürtig, Harald Schwalbe

**Affiliations:** Institute for Organic Chemistry and Chemical Biology, Center for Biomolecular Magnetic Resonance (BMRZ), Goethe University Frankfurt am Main, Hessen, Max-von-Laue-Str. 7 60438 Frankfurt/Main, Germany; Institute for Organic Chemistry and Chemical Biology, Center for Biomolecular Magnetic Resonance (BMRZ), Goethe University Frankfurt am Main, Hessen, Max-von-Laue-Str. 7 60438 Frankfurt/Main, Germany; Institute for Organic Chemistry and Chemical Biology, Center for Biomolecular Magnetic Resonance (BMRZ), Goethe University Frankfurt am Main, Hessen, Max-von-Laue-Str. 7 60438 Frankfurt/Main, Germany; Institute for Organic Chemistry and Chemical Biology, Center for Biomolecular Magnetic Resonance (BMRZ), Goethe University Frankfurt am Main, Hessen, Max-von-Laue-Str. 7 60438 Frankfurt/Main, Germany

## Abstract

Riboswitches are gene regulatory elements located in untranslated mRNA regions. They bind inducer molecules with high affinity and specificity. Cyclic-di-nucleotide-sensing riboswitches are major regulators of genes for the environment, membranes and motility (GEMM) of bacteria. Up to now, structural probing assays or crystal structures have provided insight into the interaction between cyclic-di-nucleotides and their corresponding riboswitches. ITC analysis, NMR analysis and computational modeling allowed us to gain a detailed understanding of the gene regulation mechanisms for the Cd1 (*Clostridium difficile*) and for the pilM (*Geobacter metallireducens*) riboswitches and their respective di-nucleotides c-di-GMP and c-GAMP. Binding capability showed a 25 nucleotide (nt) long window for pilM and a 61 nt window for Cd1. Within this window, binding affinities ranged from 35 μM to 0.25 μM spanning two orders of magnitude for Cd1 and pilM showing a strong dependence on competing riboswitch folds. Experimental results were incorporated into a Markov simulation to further our understanding of the transcriptional folding pathways of riboswitches. Our model showed the ability to predict riboswitch gene regulation and its dependence on transcription speed, pausing and ligand concentration.

## INTRODUCTION

Riboswitches are RNA regulatory elements that modulate gene expression. Metabolites of low molecular weight act as inducer molecules that bind to riboswitches. The majority of riboswitches are located in the 5′-untranslated region (5′-UTR) of mRNAs (1–3). The inducer molecules range from cations and anions over coenzymes to amino acids, nucleobases and nucleotide derivatives to cyclic di-nucleotides (CDNs) including cyclic di-guanosine monophosphate (c-di-GMP) and cyclic guanosine-adenosine-monophosphate (c-GAMP) (4–7).

Riboswitches contain two parts, an aptamer domain for ligand recognition and an expression platform for gene regulation. The regulatory power of riboswitches stems from distinct conformational transitions induced upon ligand binding. In case of transcriptional riboswitches, regulation has been shown to occur during transcription either by formation of a terminator state or avoidance of this conformation. These non-terminator conformations are commonly summarized as antiterminator conformation (1). Depending on whether ligand binding stabilizes the antiterminator or terminator conformation, riboswitches can act as ON- or OFF-switches either enabling or repressing gene transcription, respectively.

**Figure 1.**
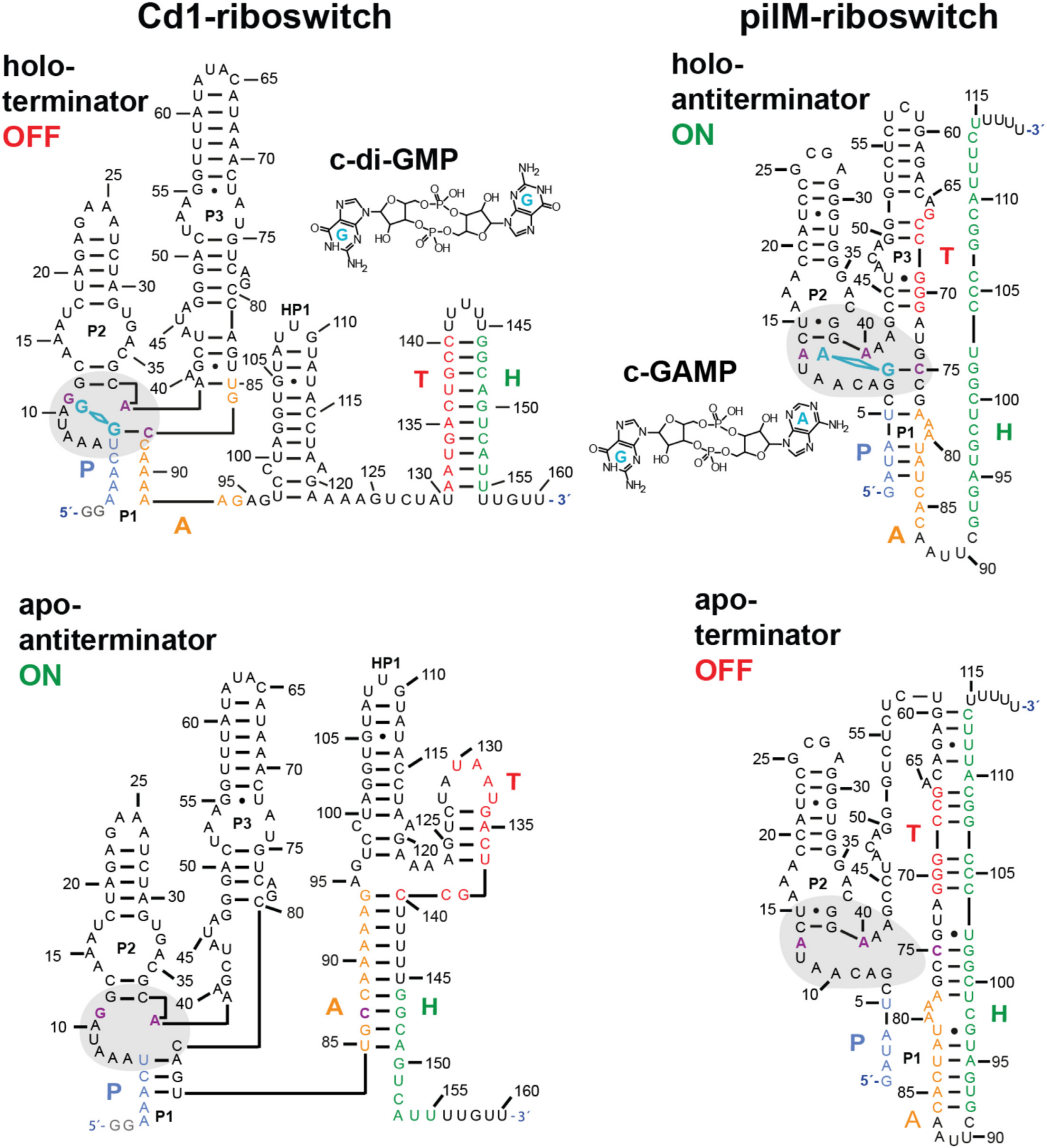
Nucleotide sequence and secondary structures of full-length pilM and Cd1 riboswitches in their ligand-bound conformation with their cognate ligands c-di-GMP and c-GAMP, respectively. The bound ligands are indicated in turquoise and stems are annotated with P1, P2 and P3. The nucleotides of the binding pocket are indicated in violet. The strands that contribute to riboswitch function are color-coded in the P1ATH nomenclature in P1(blue), A(orange), T(red) and H(green). The 5′-aptamer strand P pairs an aptamer-stabilizing strand A to form the binding capable aptamer. The switching strand T and the terminator strand H are located downstream on the mRNA. For Cd1, the terminator conformation is formed through interactions of strands T + H and antiterminator through T + A + H. For pilM, the terminator conformation is formed through interactions of strands T + A + H and antiterminator through P + A. Additionally, the binding pockets are marked with a grey background.

Riboswitches that bind CDNs have been shown to be part of regulatory networks involving genes for the environment, membranes and motility (GEMM) (8). External stimuli cause the formation or degradation of CDNs through cyclases and phosphordiesterases, respectively. The resulting change in signaling molecule concentration alters gene expression at multiple riboswitch-controlled loci and leads to lifestyle changes including biofilm formation and pili expression (9). These lifestyle changes have been shown to be key factors of pathogen virulence in *Vibrio cholera* and *Clostridium difficile* (10,11). c-di-GMP is crucial in the regulation of mobility lifestyles in gram-positive and negative bacteria. For gram-positive bacteria, including *C. difficile*, it was shown that elevated levels of c-di-GMP lead to biofilm formation (9,12). Several *in vitro* studies have linked the formation of biofilms to increased antibiotic resistance, and pilus protein expression to toxin production in *C. difficile* (13–16). During colony and biofilm formation, *C. difficile* switches its protein expression from flagella to pilus proteins. As consequence, *C. difficile* gains increased antibiotic resistance and a rapid growth of the population during prolonged antibiotic treatment (17,18). The increase in *C. difficile* population is the main cause of about 95% of pseudomembranous colitis cases. These inflammatory diseases originate from the production of toxins TcdA and TcdB (19–21). In the complex network of factors that contribute to regulation of flagellar proteins in *C. difficile*, the Cd1-riboswitch (Figure 1) is located directly upstream of eleven genes coding for flagellar proteins. The riboswitch seems to function in tandem with a *cis*-acting DNA sequence that determines in large part whether individual bacteria produce flagellar proteins (22). Interference with riboswitch regulation may therefore provide an opportunity to target biofilm formation and in turn antibiotic resistance. The Cd1-riboswitch is a transcriptional OFF-switch. When the native ligand c-di-GMP is bound, expression of flagellar proteins is down-regulated (23).

The c-GAMP-sensing riboswitch class can be found in several gram-negative bacteria (6,10,24). Interestingly, due to the high similarity in secondary structure, c-GAMP-sensing riboswitches were initially annotated as c-di-GMP-sensing riboswitches (24). Keller *et al.* were recently able to demonstrate that c-GAMP riboswitches bind c-di-GMP using a stably protonated adenine in the ligand binding pocket (25). Beyond showing a highly similar binding mode in crystal structures, it could also be shown that single point mutations in the lower part of P2 can affect ligand affinity and selectivity between c-di-GMP and c-GAMP (26). An interesting organism in this context is *Geobacter metallireducens* since it utilizes both, c-GAMP and c-di-GMP signaling. *G. metallireducens* gene expression is regulated by thirteen loci controlled by c-GAMP-sensing riboswitches (6). One of these loci is a polycistronic gene coding for pili. This pilM gene enables *G. metallireducens* to utilize metal oxides as an electron sink under anaerobic conditions. The highly conductive pili form a root-like network interconnecting the organism and the metal oxide particles while transferring electric charges (27). The presence of these interconnections enables *G. metallireducens* to thrive under these anerobic conditions and predominate its habitat (28). Like the Cd1 riboswitch, the pilM riboswitch (Figure 1) acts on the transcriptional level, but it functions as an ON-switch. The presence of c-GAMP prevents formation of the terminator conformation in the pilM riboswitch and thus does not repress transcription of the subsequent genes. In the pilM riboswitch, the requirements for adoption of the aptamer conformation or the terminator conformation are mutually exclusive. Contrarily, in the Cd1 riboswitch these folds can coexist and compete with the formation of the antiterminator conformation. The two riboswitches therefore provide a unique opportunity to study conformational changes required to achieve opposite regulatory decisions.

In rho-independent, riboswitch-based transcriptional gene regulation, only mRNA structures that are populated co-transcriptionally contribute to the regulation mechanism. The RNAs elongating during transcription populate an ensemble of minimal energy structures and the kinetic barriers between them determines the rate of equilibration. Transcriptional riboswitches possess the intrinsic property that only barriers that can be surpassed during transcription are part of the regulatory mechanism. Due to the sequential nature of transcription, RNA transcription intermediates can populate low energy structures on multidimensional folding landscapes that constantly change as these intermediates are being elongated. These local minima structures are the initial microscopic or macroscopic states of subsequent refolding and substantially impact the final RNA structure (29,30). In the case of riboswitches, a small inducer molecule can bind an intermediate aptamer domain and stabilize it. This stabilization of the aptamer domain can shift subsequent equilibria towards terminator or antiterminator folds (1–3).

The dynamic nature of riboswitch gene regulation requires sophisticated methodology to study cotranscriptional RNA folding. The methodology spans from real-time transcription observation, over roadblock systems halting transcription at desired stops, to the detailed analyses of isolated key states. Realtime folding of an adenine-sensing riboswitch during transcription was directly observed using single molecule force spectroscopy (31). PAGE analyses of single round riboswitch transcriptions pioneered by Wickiser *et al.* paired with kinetic models could show pausing as a necessary step for riboswitch gene regulation (32,33). To gain further insight into the structure of folding states, a wide range of chemical probes can be applied. Strobel *et al.* designed an assay probing all transcriptional intermediates of a RNA in a single mixture (34). Insight into sequence length-dependent reactivity of RNA residues provides key information regarding the predominately populated RNA structures. Beyond the unique feature to study single molecules, smFRET also contributes a detailed understanding of cotranscriptional processes for example how the interactions with RNA polymerase stabilizes the native fold of the preQ_1_ riboswitch, binding of the ligand signals pause site release (35).

For a detailed analysis of key states, methods for rapid RNA screening by NMR have been developed by us to minimize time for sample preparation (36). The fast sample preparation allowed us to identify and characterize structures of different transcript lengths of the *Mesoplasma florum* 2′dG-sensing riboswitch (37). Here, we utilize this previously established workflow that provides detailed base pairing information for each transcript length to gain key insight into ligand-dependent RNA refolding for the characterization of the two CDN-sensing riboswitches with close-to-identical aptamer domains but opposite regulatory readouts. Previous studies have shown the necessity of pausing for riboswitch function (33). Interestingly, the pilM riboswitch does not contain a known putative pause site beyond the termination associated site, while the Cd1 switch contains a poly-U sequence that could act as a pause site. Here, we delineate the structural basis for the opposite regulatory output and determine binding affinities and kinetics by ITC. The experimental data are used as basis for Markovian simulations allowing us to dissect the response to alteration of the key parameters: change in ligand concentration and transcription speed for transcriptional ON- and OFF-switches. The simulations also outline to which extent pausing contributes to Cd1 riboswitch function.

## MATERIALS AND METHODS

### Transcription

Plasmids were purchased from eurofins® containing the full-length native sequence of the c-di-GMP-sensing riboswitch Cd1 from *C. difficile* with an additional GG-start modification and the full-length native sequence of the pilM riboswitch from *G. metallireducens* (SI Information 1). The plasmids were transformed in *E.coli* DH5α cells and grown in LB medium. The harvested plasmids were sequenced. The plasmids were PCR-amplified to generate DNA templates for transcription. 2′-methoxy modified reverse primers were used in the transcription to generate specific lengths of RNA with high 3′-end homogeneity, the 2′-OMe-modified nucleotides are marked with square brackets in Supplementary Table S1. The forward primers were designed to contain a truncated T7 RNA polymerase promoter and the 5′-end of the riboswitch sequence. The PCR was performed in accordance with the standard protocol by New England Biolabs^®^ (0.5 μM of each primer, 200 μM dNTPs, 10 ng plasmid) using Phusion polymerase. Sample integrity was ensured through native and denaturing PAGE (Supplementary Figures SI1 and SI2).

### RNA and ligand preparation

All transcriptions were conducted *in vitro* using T7 RNA polymerase. DNA template PCR mixtures were directly applied for transcription without further purification. Transcription mixtures contained transcription buffer (250 mM Tris–HCl pH 8.1), 2 mM spermidine, 20 mM dithiothreitol (DTT), 10 or 20% (v/v) DMSO, NTPs were added in sequence adjusted amounts up to 6 mM for the most occurring, 15 to 30 mM Mg(OAc)_2_, 1 u/ml yeast inorganic pyrophosphatase (New England Biolabs) and 40 μg/ml T7 RNA polymerase. DMSO and Mg(OAc)_2_ concentrations were optimized for preparative transcriptions (PAGE preparative transcriptions shown in Supplementary Figure S1 and S2). Unlabeled NTPs were purchased from Carl Roth GmbH + Co. KG (Germany). ^13^C,^15^N-labeled NTPs were purchased from Silantes GmbH (Germany).

The transcripts were purified according to the protocol developed by Helmling *et al.*(36) After transcription in 3 to 25 ml scale, the RNAs were washed several times with NMR buffer (50 mM Bis–Tris buffer, pH 6.1, with 120 mM NaCl and 8 mM MgCl_2_ for Cd1, or 50 mM KCl and 5 mM MgCl_2_ for pilM) in 3–10 kMWCO (molecular weight cut-off) centrifugal concentrators (Vivaspin 20^®^ from Sartorius AG, Germany). These NMR buffer conditions were chosen instead of the standard potassium phosphate buffer regularly used for nucleic acid NMR, since c-di-GMP is known for its ability to form G-quadruplexes with K^+^ ions in solution.(38–40) The final concentration of the stock solution was determined using OD measurements on a Nanodrop (ThermoFisher) and nearest-neighbor corrected extinction coefficients.

Samples of the Cd1 riboswitch RNAs required for secondary structure determination and ITC measurements were purified by HPLC. The purification was performed on an HPLC-System of Hitachi/VWR using a Kromasil RP18 100A 5 μm 10 × 250 mm column. For buffer A, 50 mM potassium phosphate at pH 5.9/2 mM tetra-butyl-ammonium-hydrogen sulfate were used. Buffer B was composed of 40% buffer A and 60% Acetonitrile. The temperature was set to 60°C. Cd1 RNAs were refolded by snap cooling (5 min 95°C followed by 20 min on ice) before NMR and ITC measurements.

C-GAMP and c-di-GMP were purchased from Sigma Aldrich or in the case of ^13^C/^15^N-labeled c-di-GMP provided by Heiko Keller (25).

### Cd1 mutations

Modified RNA constructs G11C, G11A, C81A, G87A and G88A were produced by ordering DNA templates from eurofins listed in Supplementary Table S2. For RNA A24-26U we also ordered a DNA template but amplified the DNA by PCR as described above by using the primers for Cd1^88^.

### NMR spectroscopy

NMR-samples with a volume of 180–280 μl contained 50 mM Bis–Tris buffer, 110 mM NaCl (Cd1) or 50 mM KCl (pilM), 5–10% v/v D_2_O, 100–1600 μM RNA. While pilM measurements were carried out at 5 mM MgCl_2_, Cd1 measurements were performed at [RNA]:[MgCl_2_]-ratio of 1:40. TSP was added as internal reference, and all measurements were conducted in Shigemi NMR tubes (Shigemi Inc.) or 3 mm NMR tubes for automation (Bruker). Bruker AV600, AV700, AV800, AV900 and AV950 spectrometers, equipped with cryogenic probes were used for NMR experiments. Data were processed with Bruker Topspin 3.5 (Bruker Biospin) and Sparky 3.14 (Supplementary Figures S3–S10). In ^1^H 1D and ^1^H/^1^H NOESY spectra (Supplementary Figure S13), water suppression was achieved using jump-and-return echo pulse schemes (41).

### ITC

ITC measurements were performed on a MicroCal iTC200 (GE). The RNAs were provided in a concentration range from 50 to 80 μM in a measurement volume of 205 μl. Individual measurement condition for every ITC measurement are shown in Supplementary Figure S11 and S12. CDNs were added in a 10-fold higher concentration then the RNA in 4 μl steps after an initial 0.4 μl step. For ITC-data analyses the program Nitpic was used for raw data processing and integration.(42) The final fits were obtained using SEDPHAT (Supplementary Figure S11 and S12) (43). Determination of *k*_on_ and *k*_off_ rates was carried out using the equilibration time curve (ETC) fitting of the KinITC software (Affinimeter Supplementary Figure S13).

### Cotranscriptional folding simulation

Markovian simulations were performed to compare the folding processes of the Cd1 and the pilM riboswitches modelling transcription elongation (44). The model transitions of the transcription intermediates involves refolding from an initial state to three different states for each RNA with various length. The states represent an *apo* state with a ligand free aptamer, the terminator for pilM and the antiterminator for Cd1 and as third state a *holo* state where ligand is bound to aptamer. The kinetics of transitions between states are modelled by first order rate equations. The three states for each length are interconnected through a total of six rates. The *apo holo* transitions are described by the corresponding *k*_on_ and *k*_off_ rates determined in ITC measurements. In ITC data collected for numerous pilM riboswitch transcript lengths, a constant *k*_on_ rate was observed. This finding was extrapolated to the Cd1 riboswitch and also for states which did not allow an accurate *k*_on_ rate determination due to low affinity. The *k*_off_ rates were derived by dividing the *K*_D_ with the constant *k*_on_ rates. Constructs without measured *K*_D_ were interpolated using the mean of adjacent *K*_D_ values. The *apo* terminator/antiterminator transitions used the difference in formed base pairs to derive transition rates. This procedure utilized the stability predictions published by Fürtig *et al.* (45). The *holo* and terminator/antiterminator transitions also used the difference in formed base pairs unless the *k*_off_ rate for unbinding of the ligand had a lower rate. The rate values of state transitions are illustrated in Supplementary Figure S14. The riboswitch behavior was accessed for changes in ligand concentration, transcription speed, base pair closing and pausing duration. To account for the fact that RNAs are occluded from folding while hybridized or in the exit tunnel of the RNA polymerase, pausing was set to occur 10 nucleotides before the pause site at nucleotide 145. This setting allows only residues to engage into folding that have left the polymerase at the time of encountering the pause site. With this adjustment only states with a 10 nucleotide state located within the exit tunnel are able to refold (46).

## RESULTS

### Secondary structure of the aptamer domains of pilM and Cd1 riboswitches and their key nucleotides involved in ligand binding and conformational stabilization

#### NMR chemical shift assignment of the Cd1 Aptamer

**Figure 2.**
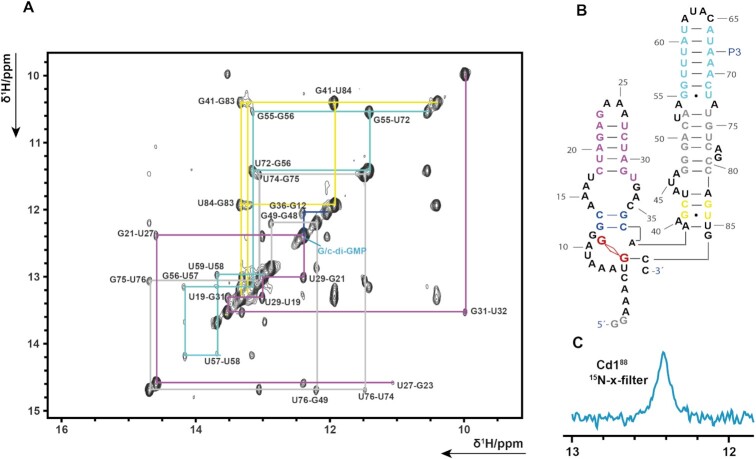
(**A**) shows the imino region of Cd1^88 1^H/^1^H NOESY spectrum. The assignment walks in the spectrum are indicated in the same color scheme as was used in B). (**B**) The Secondary structure model for Cd1-88 when bound to c-di-GMP is depicted. (**C**) Experiments on a sample containing only c-di-GMP in ^13^C/^15^N-labeled form. A single peak is observed in the imino region in a ^1^H/^15^N x-filter 1D experiment unambiguously showing that the detected G–C base pair is formed between the ligand c-di-GMP and the RNA.

The pilM- and Cd1-sensing riboswitches are classified as part of the GEMM I family of riboswitches which contain three helices P1, P2 and P3 and form a three-stem junction in the ligand-bound state.(47) Investigations on the similarly classified Vc2 riboswitch from *V. cholerae* showed that the P1 helix is crucially important for ligand binding, even though the Cd1 riboswitch is described in recent literature as lacking the P1 helix (12,22,48). This contradiction, and the lack of structural data in the literature, opens questions regarding how ligand binding is achieved in the Cd1 aptamer domain. Preceding the NMR characterization of Cd1^88^, the construct Cd1^98^ was investigated as it was assumed to be a good model for the aptamer domain which could possibly form a P1 helix. Initial results seemed promising as significant changes could be detected that occur in the NOESY spectra of Cd1^98^ upon addition of c-di-GMP (Supplementary Figure S4A and B). NMR resonance assignment, however, was impossible as the number of signals did not match with the number of possible base pairs. During the search for the minimal structural motif of the aptamer domain, we discovered Cd1^88^ to yield the best resolved ^1^H/^1^H NOESY spectra, showing well resolved cross peaks of several neighboring imino hydrogen base pairs indicative for a single stable RNA stem structures. Thus, the final NMR resonance assignment was performed using NOESY data of this construct (Figure 2). With the assignment of Cd1^88^ in hand, it became apparent that spectra of Cd1^98^ feature signals arising from conformations adopted by P2 in its unbound state (Supplementary Figure S4 A and B indicated with orange). To clarify which imino signals were corresponding to a guanosine (G) or a uridine (U) nucleotide the common ^1^H/^15^N HSQC assignment strategy was insufficient due to G/U signal overlap in the spectral region (49). Instead, two samples of Cd1^98^ were transcribed either with ^15^N labeled U or with ^15^N labeled G. With these samples, ^15^N x-filter NOESY type spectra were measured. In this type of spectra, cross peaks are observed only for imino protons that are bound to a ^15^N atom (Supplementary Figure S7). This strategy allowed us to transfer the GU assignment from our datasets of Cd1^98^ to the new Cd1^88^ datasets which matched nicely allowing for the assignment of Cd1^88^ by matching the cross peak pattern of Cd1^88^ with the cross peak pattern of two model construct for the P2 and P3 stem (Supplementary Figure S3) through application of a divide-and-conquer strategy and standard imino walk assignments as also recent literature examples show (50).

Analysis of the cross peak patterns showed that the signals of P2 (Figure 2, indicated in violet) shift strongly upon binding to c-di-GMP while for the signals of P3 (Figure 2, indicated in turquoise) only small changes are observed. Additionally, the P3 stem seems to be unstable on its own as only the base pairs from U59-G56 are visible in NMR spectra of the model construct. Using ^13^C/^15^N labeled c-di-GMP and ^15^N x-filter 1D NMR spectroscopy we could show that the ligand forms one GC base pair with the riboswitch (Figure 2C and Supplementary Figure S6).

#### NMR chemical shift assignment of the pilM riboswitch

**Figure 3.**
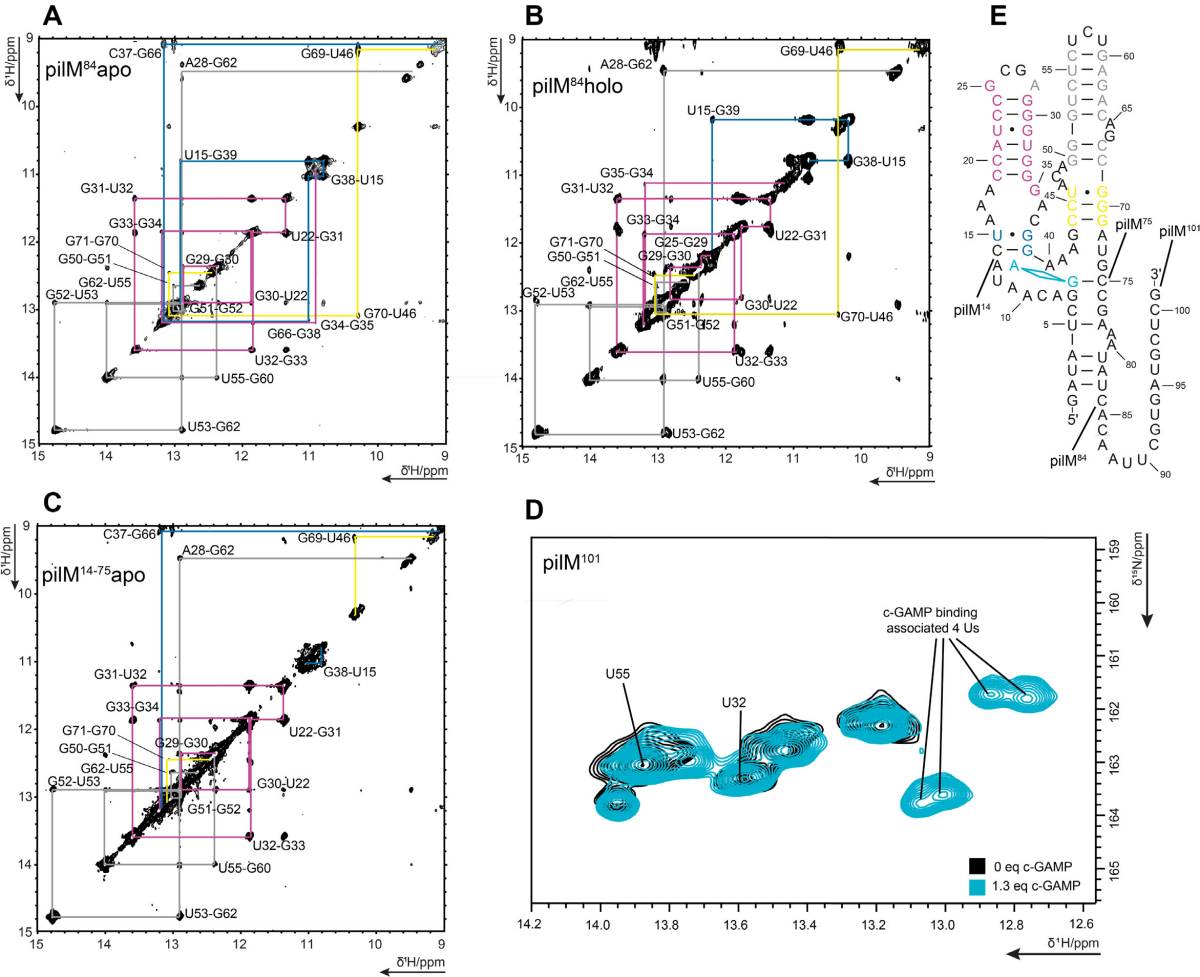
Imino region of ^1^H/^1^H NOESY spectrum for pilM^84^ in *apo* state (**A**), *holo* state (**B**) and double truncated pilM^14-75^ (**C**). (**D**) An overlay of the pilM^101^*apo* (black) and a *holo* (blue) spectrum with four additional Us resulting from c-GAMP addition. (**E**) The pilM secondary structure indicating the key last residues and the start residue of pilM^84^, pilM ^101^ and pilM^14–75^. The assignment walks of (A), (B) and (C) are indicated in the same color scheme as was used for residues in the secondary structures in (E).

The pilM riboswitch features a more stable P1 stem compared to the Cd1 riboswitch. An almost complete imino assignment could be accomplished through a combination of ^1^H/^1^H-NOESY and inspection of the crystal structure of a homologous RNA (26) with and without native ligand and the assignment of a P1-truncated pilM RNA (Figure 3). The NMR assignment was facilitated by the presence of multiple GU base pairs in the secondary structure, with their characteristic strong cross peaks in the region between 12 to 9 ppm. These GU imino signals allowed a complete assignment of the nucleotides found in the P2 and the P3 stems of the pilM riboswitch. Nevertheless, the assignment of P2 and P3 presented multiple challenges to overcome: the upper part of the P3 stem is inherently difficult to assign due to its palindromic sequence of GUGUG. The assignment was further complicated by the fact that G52 and G62 have the same chemical shift. We observed a cross peak originating from a C2H2 side of A28 to the imino NH of G63. This observation is in line with a distances of ∼5 Å from A28 H2 to G63 H1 as derived from the crystal structure (26). The identity of this cross peak to originate from an adenosine C2H2 side was confirmed in NOESY experiments with selectively ^13^C labeled adenosine residues. Thus, we postulate that due to stacking within this loop-loop interaction, the cross peak with chemical shifts (δ(^1^H) = 9.5 ppm, δ(^13^C) = 156 ppm) lies outside the typical chemical shift region. Additional cross peaks from G62 to A28 enabled an unambiguous assignment of the palindromic region.

Comparison with the P1-truncated pilM^14–75^ aptamer showed that almost all imino signals of the entire pilM aptamer domains originate from nucleotides found in the P2 and P3 stem (Figure 3C). After ligand addition, several large chemical shift changes can be detected in the NOESY spectra of the pilM aptamer (Figure 3B). One of the largest up field shift occurs for the lower GU base pair of U15 and G38 in the P2 stem. G38 shifts 0.2 ppm from 11.0 ppm to 10.8 ppm and U15 shifts 0.6 ppm from 10.8 ppm to 10.2 ppm. Interestingly, G38 does not just have a NOESY cross peak to G39 in accordance with secondary structure predictions, but an additional cross peak to a second base pair. In the *holo* crystal structure by Ren *et al.*, G66 is base paired to C37 and located right on top of G38. The distance observed between G38 and G66 is longer than 5 Å. It can reasonably be assumed that *apo* pilM shows a G38–G66 cross peak (Figure 3A) that is lost after ligand addition, because the distance between the guanosines becomes larger and undetectable in NOESY experiments (Figure 3B). This observation also indicates significant structural changes near the binding pocket. A second interesting signal to observe is the most down field signal U53. It shifts further downfield by 0.04 ppm from 14.78 ppm to 14.82 ppm when ligand is bound. To aid assignment, ^1^H–^15^N correlation spectra were acquired (Figure 3D). For the *holo* RNA these show additional U signals in the spectral region from ^1^H 13.1 ppm to 12.7 ppm and ^15^N 161 ppm to 164 ppm. Due to the limited chemical shift resolution in this region, no NOESY cross peaks could be resolved and thus unambiguously assigned, but it is very likely that these signals correspond to the U residues found in the lower part of the P1 stem, indicating that ligand binding stabilizes the pilM structure and its P1 stem.

### Secondary structure of full length Cd1-riboswitch

Reaching nucleotide 148, the NOESY spectra of Cd1 constructs change significantly compared to the NOESY spectra of preceding constructs Cd1^88^ and Cd1^133^. The signals indicative for a stabilized helix P3 vanish and cross peaks indicative for helix P2 bound to c-di-GMP reappear at their resonance position characteristic for the unbound state. As the spectral resolution decreases with the size of the RNA, the unambiguous assignment of the longer transcript lengths becomes increasingly difficult due to limited chemical shift resolution. To circumvent this problem, we recorded NOESY spectra of model constructs for the Cd1 P2, HP1 stem and the terminator-stem (Supplementary Figure S3) and compared their respective cross peak patterns with that of the Cd1^fl^ RNA. This comparison shows that the P2, P3, HP1 and the terminator stem are formed in the full-length riboswitch Cd1^fl^.

### Binding competence of different transcript lengths

A transcriptional riboswitch needs to enact its switching function within the time limit set by the ongoing transcription of binding-competent RNA transcription intermediates of varying lengths. For the two riboswitches studied here, the minimum and maximum lengths of transcript lengths with ligand binding capability were determined using NMR and ITC within the regulatory window at single-nucleotide resolution.(36) For this, the construct lengths were extended or shortened starting from the literature know aptamers lengths until ligand binding did no longer occur. In some cases, additional NMR signals upcoming in extended RNA construct can either be directly attributed to the new residue forming a base pair or the stabilization of a different base pair. Based on the predicted secondary structure of pilM, it is possible to differentiate between additional residues for stems that are expected to contribute new imino signals and loop residues that will not contribute any additional signals.

**Figure 4.**
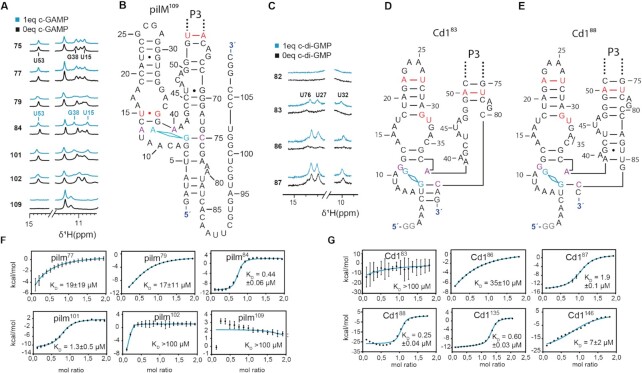
(**A**) and (**C**) show the NMR reporter signal regions of a ^1^H NMR titration, for c-GAMP-binding to pilM-RNAs ending on nucleotides 75, 77, 79, 84, 101, 102 and 109 and for c-di-GMP binding to Cd1-RNAs ending on nucleotides 82, 83, 86, and 87. Indicated in turquoise are the spectra of metabolite-containing samples and in black samples without. Complete NMR spectra of the shown datasets and all other lengths measured are shown in the Supplementary Figure S8, S9 and S10, the full peak lists can be found in Supplementary Table S3 and S4. (**B**), (**D**) and (**E**) show the secondary structure models for ligand recognition, indicated in violet are the key nucleotides of the binding pocket and highlighted in red are the bases contributing to the reporter signals. Section (**F**) and (**G**) show examples of the ITC titration curves for constructs relevant in ligand recognition. Values and errors in the *K*_D_ are derived from triplicate measurements, error bars indicated in the ITC plots refer to the individual fit.

Ligand titration conducted for the pilM riboswitch constructs of different lengths showed a ligand binding-sensitive window for transcript lengths comprising nucleotides 77–102. Interestingly, while the mRNA chain of 77 nucleotide length did not show a change in the NMR-spectra in the absence or presence of ligand we were able to detect a *K*_D_ of 19 μM in our ITC measurements (Table 1). The aptamer gains its full binding capability at a length of 84 nucleotides with a *K*_D_ of 0.44 μM. The 1D NMR spectrum of pilM^84^ shows a down field shift of U53 at 14.8 ppm and a characteristic change at 11 ppm upon ligand addition (Figure 4A). This characteristic change corresponds to a structural rearrangement in the GU base pair closest to the binding pocket resulting in upfield shifts of U15 and G38. These among several other changes could be consistently observed for all tested constructs with lengths between 79 and 101 nucleotides. For construct 102, these ligand-binding associated chemical shift changes could no longer be detected. Further extension showed a major restructuring of the RNA fold for a length of 109 evident by loss of the two reporter peaks resonating at 14.8 and 11 ppm, respectively (Figure 4A).

**Table 1.** K_D_ determination by ITC for ligand binding to riboswitch RNA transcripts of different lengths. Values were measured as triplicates. Errors represent the standard deviation of the determined triplicate values. Sequence-aligned constructs are shown in the same line

Construct	*K*_D_/μM for c-GAMP	*k*_on_/1/M*s for c-GAMP	*k*_off_/1/s for c-GAMP	Construct	*K*_D_/μM for c-di-GMP	*k*_on_/1/M*s for c-di-GMP	*k*_off_/1/s for c-di-GMP
				Cd1^83^	>100		
				Cd1^86^	35 ± 10		
pilM^75^	>100						
pilM^14-75^	12 ± 5			Cd1^87^	1.9 ± 0.1	6300 ± 800	0.0119 ± 0.0015
pilM^77^	19 ± 19			Cd1^88^	0.25 ± 0.04	21000 ± 300	0.0045 ± 0.0005
pilM^79^	17 ± 11						
pilM^80^	8.5 ± 1.4						
pilM^82^	2.12 ± 0.17	18000 ± 8000	0.028 ± 0.012				
pilM^83^	1.67 ± 0.09						
pilM^84^	0.44 ± 0.06	28000 ± 7000	0.0037 ± 0.0009				
pilM^88^	0.25 ± 0.04	17000 ± 3000	0.0042 ± 0.0005				
pilM^91^	0.32 ± 0.05	18000 ± 3000	0.0040 ± 0.006				
pilM^93^	0.38 ± 0.06	13900 ± 1700	0.0037 ± 0.0005	Cd1^135^	0.6 ± 0.03	11900 ± 1600	0.0084 ± 0.0011
pilM^95^	1.6 ± 0.6	40000 ± 30000	0.028 ± 0.015				
pilM^96^	2.9 ± 1.5						
pilM^97^	1.6 ± 0.7						
pilM^99^	0.25 ± 0.04						
pilM^100^	0.5 ± 0.3			Cd1^146^	7 ± 2		
pilM^101^	1.3 ± 0.5						
pilM^102^	>100						
pilM^103^	>100						
pilM^105^	>100						
pilM^106^	>100						
pilM^109^	>100						

To investigate the minimum length of the aptamer domain of the Cd1 riboswitch to be binding capable, six constructs that ended with nucleotides between 80 and 98 were transcribed. Preliminary investigation of the binding behavior of the riboswitch showed that Mg^2+^ ions needed to be present in at least 40 fold excess over RNA to detect binding of c-di-GMP. Under these conditions, no changes in the ^1^H NMR spectra of Cd1^80^ to Cd1^82^ upon addition of c-di-GMP could be observed. Reaching nucleotide 83, faint characteristic reporter peaks for ligand binding can be detected in the NMR spectra as shown in Figure 4C. Interestingly, the corresponding *K*_D_ for Cd1^83^ could not be determined by ITC. While NMR clearly shows an interaction, ITC analyses suggest a *K*_D_ higher than 100 μM. Comparing the NMR spectra for Cd1^83^ and Cd1^86^ the faint emergence of reporters U76, U27 and U32 become stronger as more nucleotides are added to the aptamer domain. This is possibly attributed to the temporary formation of a small helix that stabilizes binding as shown in Figure 4D. Reaching nucleotide 87, the binding-competent conformation of the aptamer domain is preformed as the reporter signals can be observed even before the addition of c-di-GMP. For these transcripts, addition of c-di-GMP only increases the intensity of the reporter signals. The same preformation can be observed for Cd1^88^ but vanishes for longer transcripts.

As shown by Sudarsan*et al.*, the Cd1 riboswitch is a transcriptional OFF-switch. Thus, binding of the inducer ligand c-di-GMP promotes folding of the terminator conformation (5). Interestingly, to adapt the antiterminator conformation the riboswitch needs to loose, at least temporarily, its ligand binding capability. Utilizing the same methodology as for the determination of shortest transcript length capable to bind inducer ligand, we measured 2D ^1^H/^15^N-heteronuclear correlation experiments beginning with the transcript length Cd1^133^ as reference point for larger constructs.

**Figure 5.**
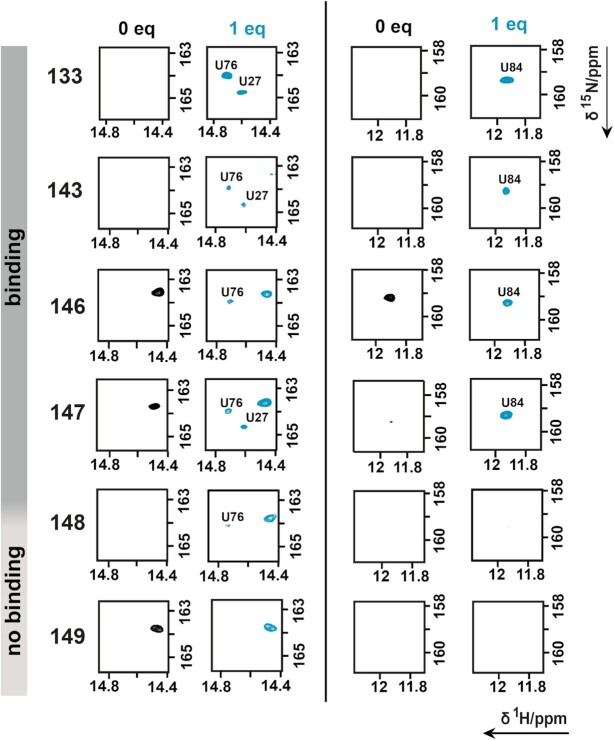
Titration followed in 2D ^1^H/^15^N correlation experiments of Cd1^133,143,146,147,148^ and Cd1^149^. Two regions R1 and R2 for ligand binding reporter peak are shown with and without ligand. The entire spectra are provided in Supplementary Figure S5.

The emergence of reporter signal U84 upon addition of c-di-GMP can be tracked clearly for transcript lengths Cd1^133^ to Cd1^147^ (Figure 5). transcript lengths comprising 148 nucleotides of the Cd1 riboswitch sequence no longer bind c-di-GMP. To further elucidate the changes observed in NMR, we measured ITC experiments of key transcript lengths. Cd1^87^ binds ligand with a *K*_D_ of 1.9 μM while Cd1^88^ binds ligand more tightly with *K*_D_ of 250 nM (Table 1). Compared to Cd1^86^, Cd1^87^ shows a clear sigmoidal ITC titration curve further reinforcing our model the RNA chain changes fold from a length of 87 nucleotides onward.

#### C-di-GMP induced interaction in the Cd1 Aptamer and tertiary interactions

**Figure 6.**
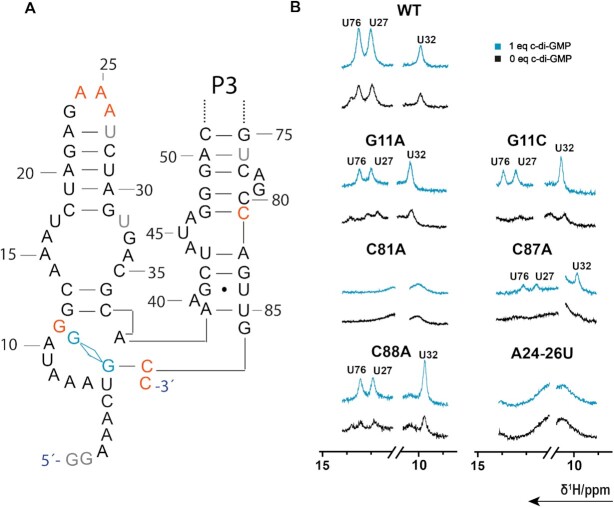
(**A**) Secondary structure of Cd1^88^ with the mutated nucleotides marked in orange and reporter nucleotides marked in gray. (**B**) The reporter peak region for c-di-GMP binding from ^1^H-1D NMR experiments. Indicated in turquoise are the spectra of c-di-GMP containing samples and in black samples without.

Our studies show that the aptamer of Cd1 length 87 adopts a more stable fold (Figure 4G and Table 1), as the *K*_D_ change indicates a refolding event. This observation raises the question whether nucleotide C87 or nucleotide C88 is the GC base pair partner of c-di-GMP. To address this question, we mutated C87 or C88 to A and compared the ^1^H-1D spectra of both constructs (Figure 6B). The reporter peaks of construct C87A are less intense than the reporter peaks in the C88A construct, leading us to conclude that C87 is the base pairing partner for c-di-GMP in Cd1. That C87A shows any response to c-di-GMP originates from residual Cd1^83^ like binding. This observation might also explain why the P1 helix is absent in the Cd1 riboswitch after Cd1^87^. With nucleotide 87 as part of the binding pocket, the frame of the nucleotide sequence is shifted in a way that no canonical helix can be formed adjacent to the binding pocket. In analogy to the *Vc2* riboswitch, nucleotide G11 of the riboswitch should bind the second guanine nucleobase of the ligand. However, our mutational studies can neither confirm nor exclude this hypothesis as there is a loss in preformation of reporter signals compared to the WT spectra but c-di-GMP binding capability is clearly not lost or enhanced. Other mutational studies show that the G11 position, equivalent to G20 in *Vc2*, rather represents a selectivity factor for different CDNs than a hard filter for ligand recognition (26,48,51).

Overall, the mutations of C87 and C11 have an impact on the secondary structure in the ligand-free state as can be seen in the reduction of preformed reporter signal compared to the WT. By comparing the mutations to each other, the specific effect of each mutation is clearly evident. This further shows that the instability of the ligand free fold and how easy it can be changed. The stabilizing and refolding effect of ligand binding seems to stem from two contributions including the tetraloop interaction of P2 to the bulk of U52-A54 which is a known tetraloop binding site in other RNAs (52,53) and the pairing of G79 with C35 as observed by Sudarsan *et al.* (22). Our mutational studies can confirm these assumptions as no binding can be observed for RNAs C81A and A24-26U.

### Cotranscriptional modeling of conformational landscape adopted during transcription of GEMM riboswitch with ON- and OFF-regulatory functions

**Figure 7.**
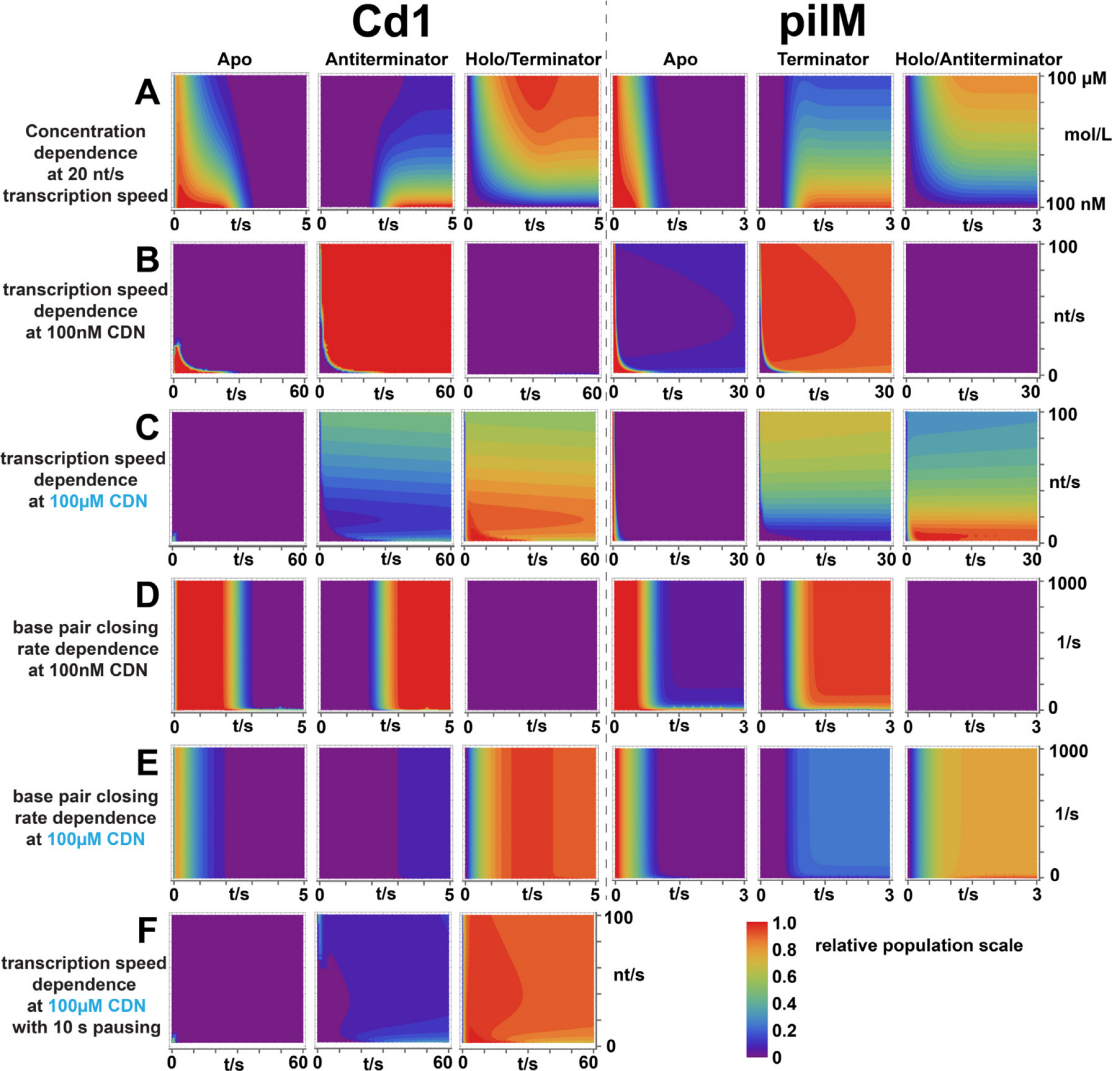
Contour level plots of cotranscriptional folding states for the Cd1 and pilM riboswitch, the population of the three functional relevant states over time is depicted in 5% levels of relative population. The population of the macrostates is the sum of all contributing RNA lengths that are in the corresponding state. The conditions of the corresponding simulation were 20nt/s transcription speed, no pausing, 400/s base pair closing rate and either 100nM or 100μM cyclic-di-nucleotide (CDN), c-di-GMP for Cd1 and c-GAMP for pilM. The second variables in the contour level plots were the concentration of CDN (**A**), the transcription speed at low ligand concentration (**B**), the transcription speed at high ligand concentration (simulated from 2 to 100 nt/s) (**C**), the base pair closing rate at low ligand concentration (simulated from 2 to 100 nt/s) (**D**), the base pair closing rate at high ligand concentration (**E**) and the transcription speed at high ligand concentration with 10 s pausing at the possible pause site at nucleotides 141–145 (simulated from 3 to 100 nt/s) (**F**). The images of Figure 7 with a lower cutoff for transcription speed, base pair closing speed and a concentration plot with higher concentrations of CDNs, is provided in Supplementary Figure S15.

In order to derive a predictive model for riboswitch function based on the experimental data discussed above, we performed Markov modeling with kinetic rates determined by ITC measurements and standard base closing rates as determined in Fürtig *et al.* (Figure 7) (54). The analyses built on the previously developed theory to model the guanine-sensing riboswitch (33). Our model introduces three parallel macrostates for each RNA length that are interconnected through transition rates (Supplementary Figure S14). The states for pilM are a ligand free apo/antiterminator fold, a competing terminator fold and a ligand-bound holo/antiterminator fold. The states for Cd1 are a ligand free apo fold, a competing antiterminator fold and a ligand-bound holo/terminator fold. For Cd1 Rate-limiting steps describing the cotranscriptional refolding between terminator and antiterminator fold are inherently linked to the number of base pairs required to break before they can subsequently form base pairs in the alternative fold. Rates requiring ligand binding and unbinding were directly obtained from our ITC measurements. For state transitions that required a binding or unbinding and refolding event our model uses the numerically lower rate corresponding to the higher reaction barrier. A few transitions in this model do not require base pairs to be broken but simply require a base pair closing and its associated rate. It is known that these base pair closing events are very fast and are generally in the ns time scale (55). As a control, we tested the impact of reducing the numerical value for the base closing rate on our model. As can be seen in Figure 7D and E, the model yields identical results for base closing rates between 100 and 1000 nt/s for Cd1 and pilM. Therefore, all base pair closing rates faster than 10ms have a similar impact on the model. Following these results, we set the base closing rate to 400 nt/s (2.5 ms) for all further calculations. To see whether our models can reproduce the switching behavior for Cd1 and pilM we simulated changes in c-di-GMP and c-GAMP concentrations to be between 100 nM and 100 μM. The Cd1 simulation resulted in <5% terminator fold at very low and a maximum of 90% at high c-di-GMP concentrations and already only 20% antiterminator fold at medium c-di-GMP concentrations from 100% at low c-di-GMP concentrations (Figure 7A). In the pilM simulation, the change in c-GAMP concentration resulted in 100% terminator fold at low and 20% at high c-GAMP concentration, the holo fold is populated to 0% at low and to 80% at high c-GAMP concentration. As the actual RNA synthesis rates of the native *C. difficile* and *G. metalireducens* RNA-polymerase are unknown, we modeled a synthesis rate of 20 nt/s for the models as average value for transcription rates described in the literature (56–59). To estimate the boundaries of this assumption, we simulated synthesis rates from 1 to 100 nt/s (Figure 7B and C). Under low c-di-GMP conditions, one can see an increasingly fast buildup of 100% antiterminator fold and as expected no terminator fold. The same simulation under high c-di-GMP conditions results in 80–100% populations for the terminator fold up to a transcription rate of 30 nt/s. After this point, in case transcription proceeds with higher rates, also the antiterminator fold starts to be populated resulting in a roughly equal population of both, terminator and antiterminator states at rates over 90 nt/s. In case of the Cd1 riboswitch, this loss of function at high transcription speed could be counteracted by a putative pause site from nucleotides 141–145. Interestingly, our simulations show that for a given concentration at low transcriptions speeds (20 nt/s), a change in the duration of pausing does not change the population of states. On the other hand, with transcription speeds over 30 nt/s pausing prevents the equilibration of the terminator and antiterminator fold (Figure 7F).

For the pilM simulation we find that at low c-GAMP concentration the change in transcription speed results in the population of 80–100% terminator fold and absence of the holo state (Figure 7B). Under high c-GAMP concentration, we encounter an even more extreme behavior as for Cd1 (Figure 7C). With increasing transcription speeds the populations of terminator and holo states start to equilibrate and reach this equilibrium at transcription speed around 50 nt/s. At transcription speed over 90 nt/s, the terminator population reaches 70% and the holo population 30% compared to the 10% terminator and 90% holo fold at low transcription speeds. Thus, the populations of the functional relevant states for regulation are markedly affected by transcription speed. While the simulations for both riboswitches show that they can perform their biological function over a broad spectrum of concentrations of inducer ligand, the pilM riboswitch appears to be much more sensitive to the transcription speed of the RNA-polymerase compared to the Cd1 riboswitch.

## DISCUSSION

The Cd1 and pilM riboswitches possess key functional roles as they regulate changes in bacterial lifestyle. To reach a holistic understanding of the metabolic cycles in which a riboswitch plays a regulatory part, one needs to understand the external and internal factors that lead to the expression of genes associated with a specific lifestyle change. External factors include the signal cascades and enzymes that govern ligand production and degradation. Important internal factors are specific riboswitch-ligand interactions resulting in transcription regulation. Previous to this work, the secondary structure for Cd1 was predicted based on an analogy model to the Vc2 riboswitch from *V. cholera* (48). In contrast, a crystal structure of a pilM homolog was reported (26). We here used solution NMR, ITC and computational modelling to investigate the dynamics transcription regulation by these two riboswitches, beyond the static interaction in the reported structures.

### Determination of key nucleotides in the pilM and Cd1 riboswitch

As a first step to probe the functional conformational transitions of Cd1 and pilM, we conducted NMR experiments on transcription intermediates by single nucleotide extension. For Cd1, the base pairing nucleotide for c-di-GMP was determined to be C87 which is preceded by an alternative less stable binding conformation from nucleotides 83 to 86 (Figure 4 and 6). The base pairing RNA nucleotide for c-GAMP in pilM is known from the crystal structure to be C75. Based on the results for Cd1^87^, pilM^75^ was tested for ligand binding competence and confirmed to be binding incompetent. An extension by only two nucleotides to pilM^77^ results in a *K*_D_ of 18 μM. The construct pilM^14-75^ was prepared containing an additional truncation of the 5′-end. *K*_D_ measurements for pilM^14–75^ show a similar affinity as pilM^77^. Thus, the pilM aptamer can bind the inducer in similar manner as Cd1 with only the Watson crick residue, yet with drastically reduced affinity. The P2 and P3 junction alone functions as the binding pocket for both Cd1 and pilM. Additional P1 residues increase the pilM aptamer affinity into the nanomolar range. The extension to full aptamer pilM^84^ results in a 43 fold affinity increase compared to pilM^77^ (Table 1). In light of these results the binding incapability of pilM^75^ is likely the result of the 5′-end forming an alternative fold that is preventing the correct formation of the binding pocket. Additionally, interactions that are of vital importance for the function of the Cd1 aptamer domain could be supported by mutational studies (Figure 6). Through mutation of C81A the interaction of G79 with C35 was disrupted, rendering the aptamer binding-incompetent. Also with mutation A24–26 to U the proposed tetra loop interactions between P2 and P3 were interrupted, also leading to binding incompetence. G11 is the second key nucleotide responsible for c-di-GMP recognition according to the consensus sequence for GEMM I riboswitches, by mutating G11 to A and C but no large stabilizing or destabilizing effect could be observed with either of the two mutations (Figure 6). The nucleotide in a similar position as G11 is known in pilM to guide selectivity between CDNs, but to have no complete inhibiting effect on ligand binding. (26)

At equilibrium, transcript lengths starting from Cd1^148^ become binding incompetent. This is caused by the antiterminator fold base pairs G147 with C87 and C148 with G86, which unfolds the key part of the binding pocket (Figure 1). PilM^102^ is the first RNA transcription length that can no longer bind ligand. It contains the terminator sequence in a length that allows formation of base pairs required to stabilize the terminator conformation. It is also sequestering C75, the residue that is involved in Watson–Crick-type base pairing with the ligand cGAMP. The intramolecular interaction between C75 and G102 outcompetes the intermolecular interaction between ligand and aptamer. Both riboswitches share a loss in binding capability right after sequestering of the ligand binding Watson-Crick base pairing residues. Initial work on the deoxyguanosine-sensing riboswitch by Helming *et al.* suggested a large window of several nucleotides length of gradually diminishing binding capability, while more recent work by Binas *et al.* shows a very sharp transition for transcript lengths differing by a single nucleotide from high to no binding capability (37,60). Similar to findings for the ZMP sensing riboswitch, we observe a sharp transition in binding capability through single nucleotide extensions for pilM^101^ to pilM^102^ and for Cd1^145^ to Cd1^148^, respectively.

### The importance of the P1 helix (P1A)

The consensus sequence for GEMM I riboswitches suggests a conserved three stem junction as binding aptamer. The expectation is that a highly conserved sequence is required for binding including a fully formed P1 (47). The best known example in literature for a GEMM I switch is the translational OFF switch Vc2 (61). The key factor in Vc2 regulation is indeed the stability of the P1 helix which is key to achieve tight ligand binding, as it is known that with loss of the P1 helix Vc2 can no longer bind its ligand c-di-GMP (22). For Cd1, we could not detect NMR signals that support P1 helix formation. With c-di-GMP binding occurring at nucleotide C87, there are no residues in the secondary structure available to form a base-paired helix adjacent to the binding pocket. A drastic decrease in *K*_D_ is observed between Cd1^87^ with 1.9 μM and Cd1^88^ with 0.25 μM. In contrast to Cd1, pilM^77^ shows no drastic increase in affinity by adding a single nucleotide to the transcribed RNA. In PilM, we cannot observe indication of a formed P1 stem in absence of c-GAMP, and P1 is only formed in the presence of ligand (Figure 3D). In addition, a remarkable decrease in *K*_D_ is observed for constructs that contain P1 residues (Table 1 and Figure 4), further conforming that these pilM RNAs forms a stable P1 stem after c-GAMP addition.

This lack of P1 stability and the strong difference in the relative stability of P1 compared to P2 and P3 could not be predicted from sequence nor from x-ray structure. This finding is remarkably similar to previous reports of the stability of the P2–P3 and the instability of P1 required for the allosteric change in riboswitches (48,62). We conclude that the P2 and P3 stem represent a highly conserved aptamer scaffold that preforms a binding pocket that can be modified in its selectivity and specificity by exchanging residues of the binding pocket as discussed in the literature (26). Beyond that, there seems to be possibility for affinity fine tuning. P1 stability tuning would allow matching riboswitch sensitivity to the prevalent CDN concentration in the corresponding organism. Given that the CDN concentration is coupled its interaction within a network of several CDN-binding proteins, a tunable riboswitch affinity could be evolutionary favored. A dynamic P1 also enables a translational riboswitch based on the CDN riboswitch aptamer (61). In the example of the translational Vc2 riboswitch the P1 element plays the key role in switching between ON and OFF function.

### Cotranscriptional modelling

Our Markov modeling of the Cd1 and the pilM riboswitches provide a unifying model for the gene regulatory function of the two riboswitches despite their opposite functions to be either an ON- or an OFF-switch. We could show high sensitivity to ligand concentration within the ranges of changes in concentration associated with lifestyle changes reported in the literature (63–65). Both riboswitches showed practically no *holo* population for low ligand concentration (100 nM) and a *holo* population of 80% for pilM and 95% for Cd1 for high ligand concentration (100 μM), respectively. Beyond the impact of ligand concentration, we investigated the impact of transcription speed, pause sites and the relevance of the rate of closing base pairs. Transcription speed markedly affects the regulatory function of both riboswitches, always favoring the ligand-bound states at low speeds. This is in line with the idea that lower speeds allow for a longer presence of binding-competent states that increase the likelihood of forming a stable and refolding-resistant ligand-bound RNA complex. Based on the ITC derived *k*_off_ rates, the stable ligand-bound RNA complexes have half-life times ranging from 25 to 190 s. These stabilities make ligand release during the short transcription time windows unlikely. Interestingly, the authors of the translational Vc2 riboswitch assume that it is also kinetically controlled due to the short lifetime of mRNAs in prokaryotic cells (61). They further argue that under kinetic control a longer expression platform might increase ligand sensitivity by giving the aptamer more time to bind ligand, which is in line with our simulations. At high transcription speeds a drop in switching efficiency was observed for both riboswitches (Figure 7A). The pilM riboswitch drops off more sharply showing only 30% ON state at 100 nt/s transcription speed. This sensitivity towards the transcription speed indicates the possibility for higher level regulation through changes in transcription speed. The Cd1 riboswitch shows lesser sensitivity to high transcription speeds, due to the longer expression platform prior to the genetic decision point, compared to pilM (Figure 7). Adding a 10 s pausing delay results in a switching efficiency of 95% over a transcription speed window from 5–100 nt/s possibly extending far beyond 100 nt/s. In this case, the riboswitch sensitivity can be considered independent from the transcription speed and in consequence independent of the RNA construct length. We observe a high sensitivity of the pilM riboswitch regulatory function to changes in transcription speed, while Cd1 does not show this sensitivity.

Within the conformational transitions in riboswitches, the rate of base pair closing is by far the fastest conformational step during RNA refolding. Base pair closing drives the transition from *apo* to terminator and antiterminator folds. These rates are usually in the ns range (55,66). According to the Markov model, this base pair closing must be faster than 20 ms for Cd1 or 200 ms for pilM, else a non-regulatory riboswitch behavior would occur.

**Figure 8.**
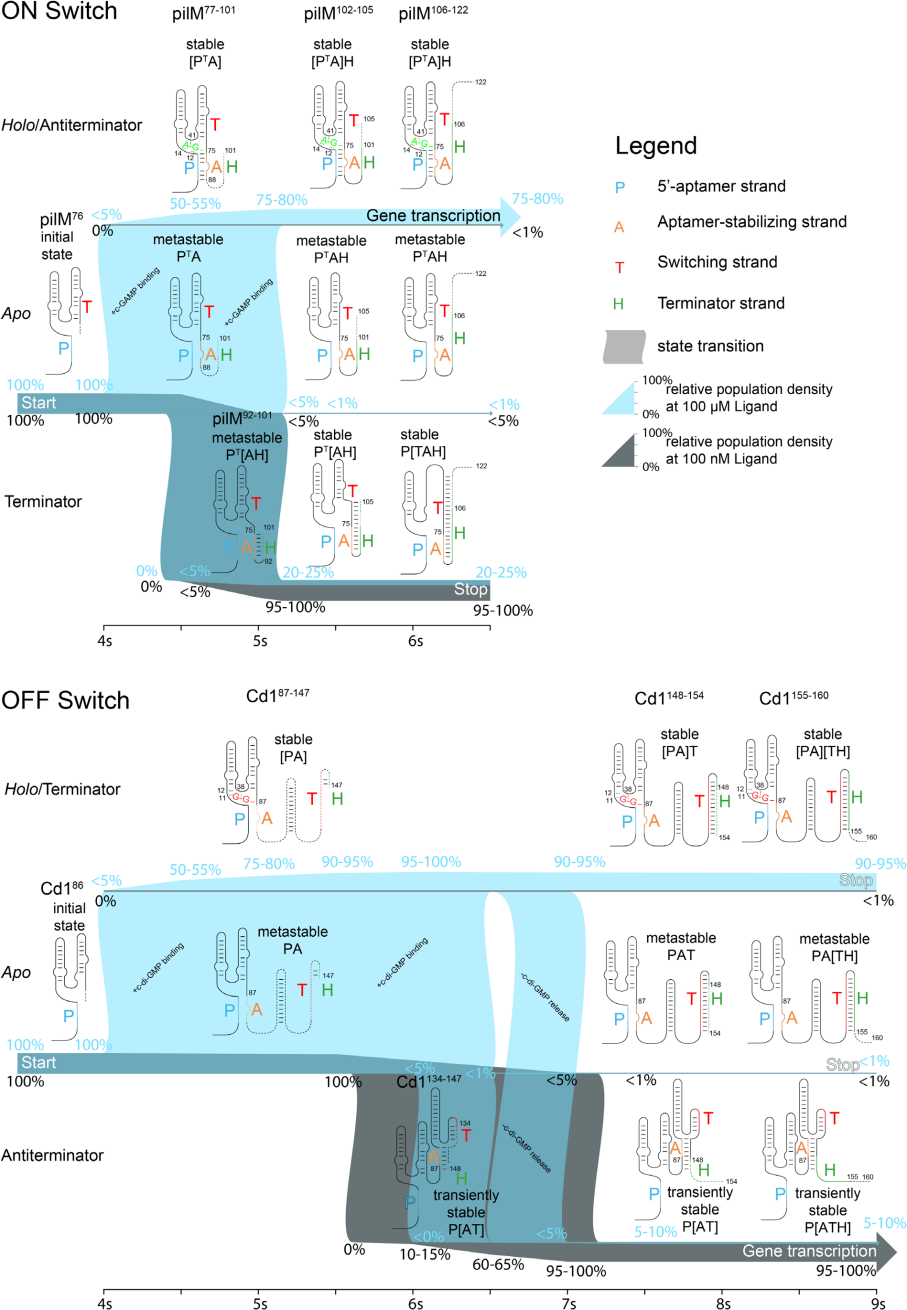
Markov model simulations of cotranscriptional folding state distribution over time for the pilM (ON) and Cd1 (OFF) riboswitch at 20 nt/s transcription speed with no pausing. State population densities are shown for 100 nM ligand concentration (gray, based on data shown in Figure 7B) and 100 μM ligand concentration in (blue, based on data shown in Figure 7C). Areas of the Figure where population densities overlap are shown in a gray-blue color. The thickness of the bar is indicative of relative population ranging from 5% to 100%. Population densities are also shown in percent at 0.5 s increments above and below the population bars if the changes by more then 5% in the timeframe. Population densities below 1% are marked explicitly. The aptamer domain is formed with nucleotide 76 and 86, when ligand can first be bound. Upon reaching nucleotide 77 and 87, the aptamer domain can bind ligand and transition up into the *holo* state. Transcription intermediates of length from 77–101 nucleotides for pilM and 87–147 nucleotides for Cd1 are binding capable. The time frames available for these transitions are indicated as state transitions. PilM can fold terminator from nucleotide 92 onward and Cd1 can fold antiterminator starting with nucleotide 134. Both RNAs transition between the three states until reaching a point of decision with transcription lengths 102 and 148 when binding is impaired.

The Markov model yields a comprehensive picture of the timeframes necessary for riboswitch-regulated gene transcription (Figure 8). We use PATH representation introduced by Helmling *et al.* and Steinert and Sochor *et al.* to discuss different structural arrangements (33,37,67). This representation describes riboswitches as assemblies of four core elements P, A, T and H. The 5′-aptamer strand P pairs an aptamer-stabilizing strand A to form the binding capable aptamer, represented as [PA]. The switching strand T and the terminator strand H are located downstream on the mRNA. In OFF switches like Cd1, the T and H strand from the terminator [TH] in addition to the ligand binding domain stabilized by [PA]. PilM is an interesting exception to common riboswitches in that its T strand is located in front of its A strand. The pilM riboswitch has a binding-competent P^T^A structure in a time window of 1.2 s, the Cd1 riboswitch has a 3.0 s window presenting the PA structure. Next, pilM starts to fold metastable P^T^[AH] terminator after 0.4 s compared to 1.5 s for Cd1 until transiently stable P[AT] antiterminator folding occurs. We differentiate between different degrees of RNA structure stability. While metastable structures can be readily refolded through ligand addition, transiently stable structures must be stable throughout the transcription process to enable the riboswitch function. The transiently stable Cd1 antiterminator structures will refold to form terminator after extended periods of time. At high ligand concentration, even before the terminator P^T^[AH] or antiterminator fold P[AT] can occur, the two riboswitches reach different levels of *holo* state population. The Cd1 riboswitch reaches 95% *holo* state population [PA] before the antiterminator conformation is folded. PilM on the other hand reaches 55% *holo* population [P^T^A] before terminator fold can occur. This results in pilM having a contested *apo* state P^T^A at the point of decision that even in the high ligand regimes folds into 20% terminator P[TAH], compared to the 5% antiterminator fold P[ATH] observed for Cd1. These results show that an early uncontested population of the *holo state* is important for both riboswitches to function in their allocated timeframe. Interestingly, the large difference in the time available for ligand binding does not result in large population differences of their *holo* states at full length challenging the idea that a larger time frame increases the likelihood of ligand binding under these conditions. Yet, there remain uncertainties in our chosen parameters. There are no data available on transcription speed and on life style-dependent CDN concentration in addition to local concentration effects at the site of transcription due to known strong localization of cyclases and phosphordiesterases at bacterial poles (68).

Riboswitch switching efficiency is imperfect, and we refer this erroneous signal transfer as leakiness. The additional time observed for Cd1 caused by the transcription of the antiterminator sequences reduces the amount of leakiness Cd1 signaling linked to structures forming that do not correspond to the correct ligand-induced switch state. Our model predicts a remarkably low leakiness for both riboswitches in the absence of cognate ligand. The Cd1 riboswitch will signal under 2% OFF signal as [PA][TH]/PA[TH] while being ON and the pilM riboswitch will only signal around 6% [P^T^A]H/P^T^AH ON signal while being OFF according to the simulations.

This low leakiness was also observed in *in vitro* single-round transcription assays for pilM (47). The leakiness increases for both riboswitches when high ligand concentrations are present, probably due to two thermodynamically favored states competing. The P[ATH] of 5–10% found for Cd1 is smaller than the corresponding 20–25% P[TAH] for pilM.

In summary, in this contribution, we delineate the secondary structures of several transcript lengths through NMR spectroscopy. The measurement of binding affinities and kinetics of the transcript lengths allow us to incorporate these biophysical data into a cotranscriptional model. This model predicts the regulatory output as function of transcription speed, base pair closing speed, pausing and concentration changes of the inducer molecules, resulting in an understanding of the key nucleotides and the temporal dynamics of state population required for riboswitch function. These biophysical studies provide a benchmark to understand the coupling of kinetics to function of kinetic riboswitches and their transcriptional intermediates.

## DATA AVAILABILITY

mFold: http://unafold.rna.albany.edu/?q = mfold

Sedphat: http://www.analyticalultracentrifugation.com/sedphat/download.htm

NITPIC: http://biophysics.swmed.edu/MBR/software.html

Sparky: https://www.cgl.ucsf.edu/home/sparky/

Topspin: https://www.bruker.com/service/support-upgrades/software-downloads/nmr/free-topspin-processing/nmr-topspin-license-for-academia.html.

## Supplementary Material

gkac514_Supplemental_File

## Notes

Present address: Tom Landgraf, Albrecht Eduard Völklein, Boris Fürtig, Harald Schwalbe, Institute for Organic Chemistry and Chemical Biology, Center for Biomolecular Magnetic Resonance (BMRZ), Goethe University Frankfurt am Main, Hessen, Max-von-Laue-Str. 7 60438 Frankfurt/Main, Germany.
