# Supplementary material for: The cotranscriptional folding landscape for two cyclic di-nucleotide-sensing riboswitches with highly homologous aptamer domains acting either as ON- or OFF-switches
Source: Nucleic Acids Res. 2022 Jun 23;50(12):6639–55. doi: 10.1093/nar/gkac514 (PMC9262584; doi:10.1093/nar/gkac514)
Supplement: gkac514_Supplemental_File [file gkac514_supplemental_file.docx]

SI Information to

**The cotranscriptional folding landscape for two cyclic di-nucleotide-sensing riboswitches with highly homologous aptamer domains acting either as ON- or OFF-switches**

Tom Landgraf^1,+^, Albrecht Eduard Völklein^1,+^, Boris Fürtig^1,^* and Harald Schwalbe^1,^*

^1^Institute for Organic Chemistry and Chemical Biology, Center for Biomolecular Magnetic Resonance (BMRZ), Goethe University Frankfurt am Main, Hessen, Max-von-Laue-Str. 7 60438 Frankfurt/Main, Germany

^+^Both authors contributed equally

*To whom correspondence should be addressed. Tel: +49 (0)69 / 798-29737; Fax: +49 (0)69 / 798-29515; Email: [schwalbe@nmr.uni-frankfurt.de](mailto:schwalbe@nmr.uni-frankfurt.de), [fuertig@nmr.uni-frankfurt.de](mailto:fuertig@nmr.uni-frankfurt.de)

Present Address:[Tom Landgraf], [Albrecht Eduard Völklein], [Boris Fürtig], [Harald Schwalbe] Institute for Organic Chemistry and Chemical Biology, Center for Biomolecular Magnetic Resonance (BMRZ), Goethe University Frankfurt am Main, Hessen, Max-von-Laue-Str. 7 60438 Frankfurt/Main, Germany.

**SI Information 1**

DNA template plasmids used for PCRs:

The DNA sequences were cloned into a Puc57 vector between EcoRI and SmaI

Cd1 sequence:

5´‑TAATACGACTCACTATAGGGAGACCTATTTAGTTTCTGATGAGAGCGAAAGCTCGAAACAGCTGTGAAGCTGTCAAACTAAATAGGCAAATCTAGAGAAATCTAGTGACGCAAAGCTATAGGGACTAAGGTTTATATACATAAACTATGTCAGCCAGTTGCCAAAAAGAGTCCTAGGTGTATTGTATACCTAAGAAAAGTCTATAATGACTGCCTTTTTGGCAGTCATTTTGTT‑3´

pilM sequence:

5´‑TAATACGACTCACTATAGGGAGATCGGAGCTGATGAGAGCGAAAGCTCGAAACAGCTGTGAAGCTGTCCTCCGATATCGACAATACTAAACCATCCGCGAGGGTGGGACGGAAAGCCTACAGGGTCTCTCTGAGACAGCCGGGATGCCGAAATATCACAATTCGTGATGCTCGGTCCCGGCATTTCTTTTTGGGTCGGCATGGCATCTCCACCTCCTCGCGGTCCGACCTGGGCTACTTCGGTAGGCTTAAGGGAGAAG‑3´

T7-Promotor, 5´-Hammerhead ribozyme, riboswitch sequence, 3´-HDV ribozyme

**SI Table 1:** PCRs primers for Cd1 and pilM. Primers are named as the corresponding construct and market with fwd for forward primer and rev for the revers primer. Nucleotides marked with [] are 2´ methoxy modified.

| **Primername** | **Primersequence** |
| --- | --- |
| Cd1^1^_fwd_ | 5´- TAATACGACTCACTATAGGAAACTAAATAGGCAAATCTAGA-3´ |
| Cd1^80^_rev_ | 5´- [GC]TGACATAGTTTATGTATAT-3´ |
| Cd1^81^_rev_ | 5´- [GG]CTGACATAGTTTATGTATA-3´ |
| Cd1^82^_rev_ | 5´-[UG]GCTGACATAGTTTATGTAT-3´ |
| Cd1^83^_rev_ | 5´-[CU]GGCTGACATAGTTTATGTA-3´ |
| Cd1^86^_rev_ | 5´-[CA]ACTGGCTGACATAGTTTAT-3´ |
| Cd1^87^_rev_ | 5´-[GC]AACTGGCTGACATAGTTTA-3´ |
| Cd1^88^_rev_ | 5´-[GG]CAACTGGCTGACATAGTTT-3´ |
| Cd1^132^_rev_ | 5´-[UU]ATAGACTTTTCTTAGGTATA-3´ |
| Cd1^143^_rev_ | 5´-[AA]AGGCAGTCATTATAGACTTTTCT-3´ |
| Cd1^146^_rev_ | 5´-[CA]AAAAGGCAGTCATTATAGACTT-3´ |
| Cd1^147^_rev_ | 5´-[CC]AAAAAGGCAGTCATTATAGACT-3´ |
| Cd1^148^_rev_ | 5´-[GC]CAAAAAGGCAGTCATTATAGAC-3´ |
| Cd1^149^_rev_ | 5´-[UG]CCAAAAAGGCAGTCATTATAGA-3´ |
| Cd1^150^_rev_ | 5´-[CU]GCCAAAAAGGCAGTCATTATAG-3´ |
| Cd1^151^_rev_ | 5´-[AC]TGCCAAAAAGGCAGTCATTATA-3´ |
| pilM^1^_fwd_ | 5´- TAATACGACTCACTATAGATATCGACAATACTAAACCATCC-3´ |
| pilM^14^_fwd_ | 5´- TAATACGACTCACTATAGCTAAACCATCCGCGAGGGTGGGACGG-3´ |
| pilM^75^_rev_ | 5´-[GC]ATCCCGGCTGTCTCAG-3´ |
| pilM^77^_rev_ | 5´-[CG]GCATCCCGGCTGTCTC-3´ |
| pilM^79^_rev_ | 5´-[UU]CGGCATCCCGGCTGTC-3´ |
| pilM^80^_rev_ | 5´-[UU]TCGGCATCCCGGCTGTC-3´ |
| pilM^81^_rev_ | 5´-[AU]TTCGGCATCCCGGCTGTC-3´ |
| pilM^82^_rev_ | 5´-[UA]TTTCGGCATCCCGGCTG-3´ |
| pilM^83^_rev_ | 5´-[AU]ATTTCGGCATCCCGGCTG-3´ |
| pilM^84^_rev_ | 5´-[GA]TATTTCGGCATCCCG-3´ |
| pilM^88^_rev_ | 5´-[TT]GTGATATTTCGGCATCCCG-3´ |
| pilM^91^_rev_ | 5´-[GA]ATTGTGATATTTCGGCATCCC-3´ |
| pilM^93^_rev_ | 5´-[AC]GAATTGTGATATTTCGGCATCC-3´ |
| pilM^95^_rev_ | 5´-[TC]ACGAATTGTGATATTTCGGCATC-3´ |
| pilM^96^_rev_ | 5´-[AT]CACGAATTGTGATATTTCGGCATC-3´ |
| pilM^97^_rev_ | 5´-[CA]TCACGAATTGTGATATTTCGGC-3´ |
| pilM^99^_rev_ | 5´-[AG]CATCACGAATTGTGATATTTCG-3´ |
| pilM^100^_rev_ | 5´-[GA]GCATCACGAATTGTGATATTTC-3´ |
| pilM^101^_rev_ | 5´-[CG]AGCATCACGAATTGTG-3´ |
| pilM^102^_rev_ | 5´-[CC]GAGCATCACGAATTGTGATAT-3´ |
| pilM^103^_rev_ | 5´-[AC]CGAGCATCACGAATTGTGAT-3´ |
| pilM^104^_rev_ | 5´-[GA]CCGAGCATCACGAATTGTG-3´ |
| pilM^105^_rev_ | 5´-[GG]ACCGAGCATCACGAATTG-3´ |
| pilM^109^_rev_ | 5´-[GC]CGGGACCGAGCATCAC-3´ |

**SI Table 2:** DNA constructs used to transcribe modified constructs of Cd1^88^.

| **Construct** | **Forward strand** | **Reverse strand** |
| --- | --- | --- |
| G11C | TAATACGACTCACTATAGGAAACTAAATACGCAAATCTAGAGAAATCTAGTGACGCAAAGCTATAGGGACTAAGGTTTATATACATAAACTATGTCAGCCAGTTGCC | GGCAACTGGCTGACATAGTTTATGTATATAAACCTT AGTCCCTATAGCTTTGCGTCACTAGATTTCTCTAGA TTTGCGTATTTA GTTTCCTATAGTGAGTCGTATTA |
| G11A | TAATACGACTCACTATAGGAAACTAAATAAGCAAATCTAGAGAAATCTAGTGACGCAAAGCTATAGGGACTAAGGTTTATATACATAAACTATGTCAGCCAGTTGCC | GGCAACTGGCTGACATAGTTTATGTAT ATAAACCTT AGTCCCTATAGCTTTGCG TCACTAGATTTCTCTAGA TTTGCTTAT TTA GTTTCCTATAGTGAGTCGTATTA |
| C81A | TAATACGACTCACTATAGGAAACTAAATAGGCAAATCTAGAGAAATCTAGTGACGCAAAGCTATAGGGACTAAGGTTTATATACATAAACTATGTCAGCAAGTTGCC | GGCAACTGGCTGACATAGTTTATGTAT ATAAACCTT AGTCCCTATAGCTTT GCG TCACTAGATTTCTCTAGA TTTGCTTATTTA GTTTCCTATAGTGAGTCGTATTA |
| G87A | TAATACGACTCACTATAGGAAACTAAATAGGCAAATCTAGAGAAATCTAGTGACGCAAAGCTATAGGGACTAAGGTTTATATACATAAACTATGTCAGCCAGTTGAC | GTCAACTGGCTGACATAGTTTATGTAT ATAAACCTT AGTCCCTATAGCTTTGCG TCACTAGA TTTCTCTAGA TTTGCCTATTTA GTTTCCTATAGTGAGTCGTATTA |
| G88A | TAATACGACTCACTATAGGAAACTAAATAGGCAAATCTAGAGAAATCTAGTGACGCAAAGCTATAGGGACTAAGGTTTATATACATAAACTATGTCAGCCAGTTGCA | TGCAACTGGCTGACATAGTTTATGTATATAAACCTT AG TCCCTATAGCTTTGCG TCACTAGATTTCTCTAGA TTTGCCTATTTA GTTTCCTATAGTGAGTCGTATTA |
| A24-26U | TAATACGACTCACTATAGGAAACTAAATAGGCAAATCTAGAGTTTTCTAGTGACGCAAAGCTATAGGGACTAAGGTTTATATACATAAACTATGTCAGCCAGTTGCC | - |

**
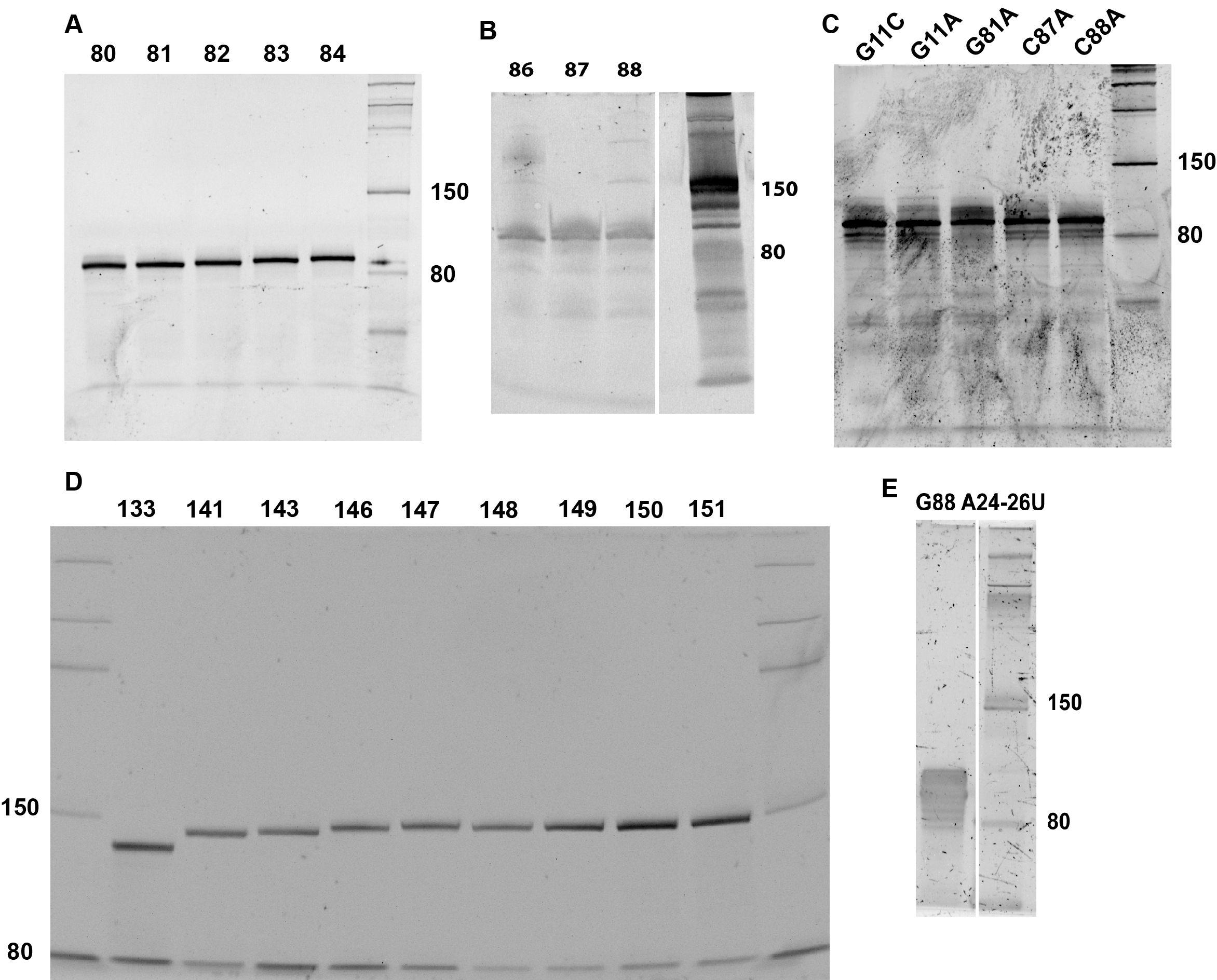
**

**SI Figure 1:** 8% denaturing polyacrylamide gel-electrophoresis with 7M urea of Cd1 RNAs **(A)**80-84 and **(B)** 86-88 at ~30°C. Gel was run for 0.5 hours at 180V using 1x TBE-buffer as running buffer. **(C)** 10% denaturing polyacrylamide gel-electrophoresis with 7M Urea of RNAs G11C-C88A at ~30°C. Gel was run for 0.5 hours at 180V using 1x TBE-buffer as running buffer. Gel was run for 0.5 hours at 210V using 1x TBE-buffer as running buffer. **(D)** 6% denaturing polyacrylamide gel-electrophoresis with 8M Urea of RNAs 133-151 at ~30°C. Gel was run for 0.5 hours at 180V using 1x TBE-buffer as running buffer. **(E)** 8% denaturing polyacrylamide gel-electrophoresis with 7M Urea of RNA A24-26U at ~30°C. Gel was run for 0.5 hours at 210V using 1x TBE-buffer as running buffer. Samples contained 1μL of a 1/100 dilution of the final RNA stock, formaldehyde, xylene cyanol and bromophenol blue. The low range ssRNA ladder (500μg/mL; 50-1k bp; New England Biolabs) was used as a reference.


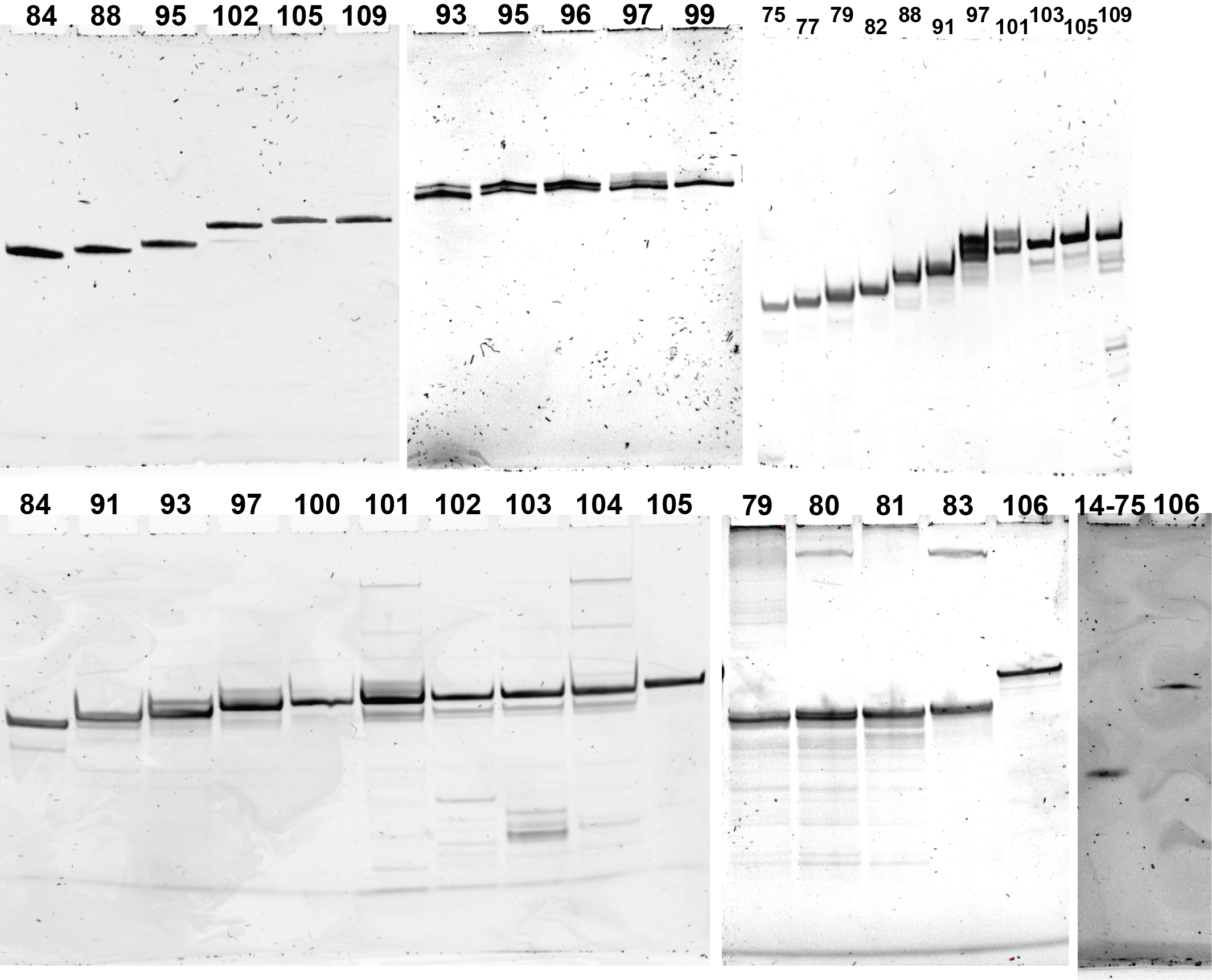


**SI Figure 2:** 10% denaturing polyacrylamide gel-electrophoresis with 7M urea of pilM14-75 and pilM RNAs 75-109 and at ~30°C. Gel was run for 0.5 hours at 200-240V using 1x TBE-buffer as running buffer. Samples contained 0.5μL of a 1/100 dilution of the final RNA stock, formaldehyde, xylene cyanol and bromophenol blue.


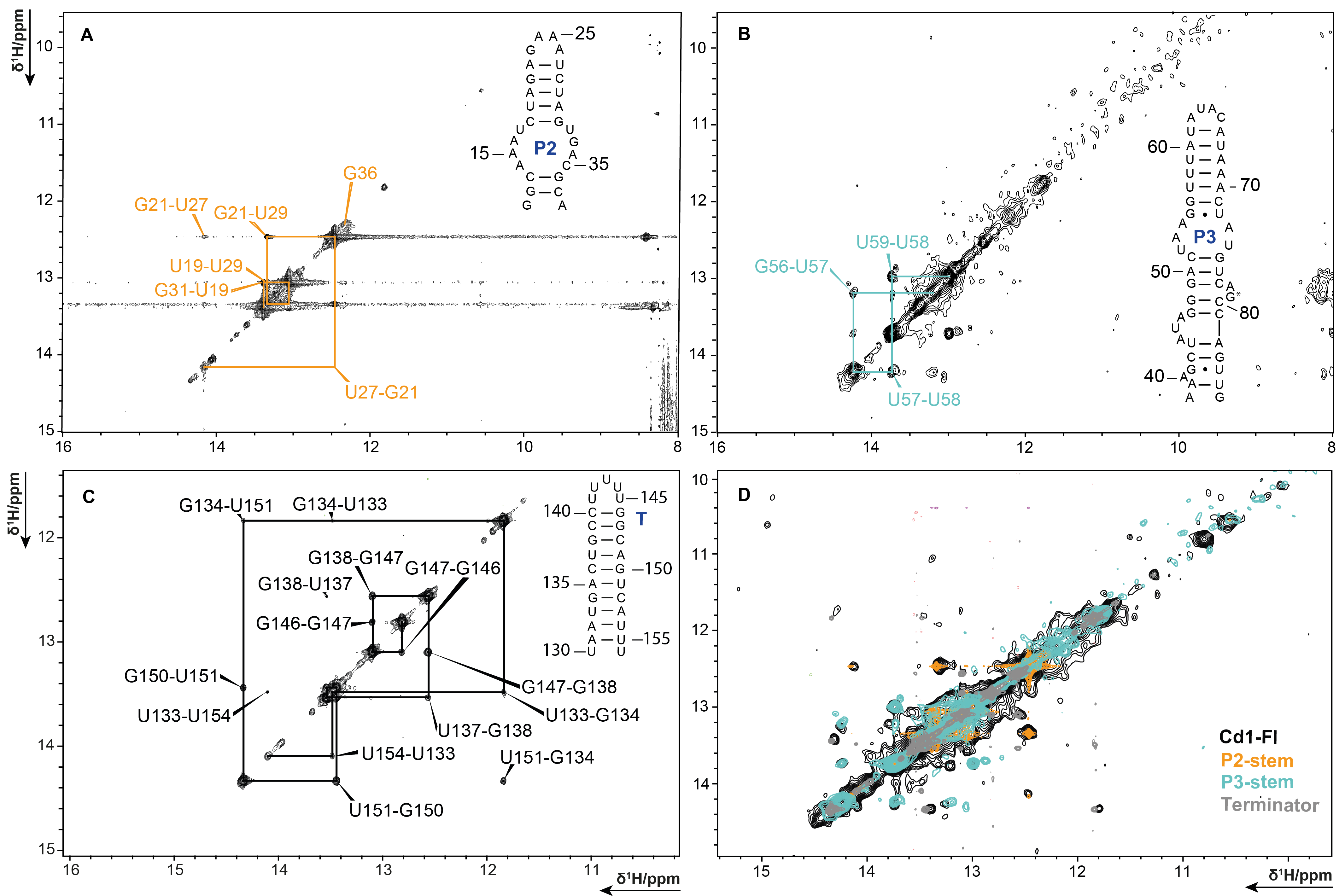


**SI Figure 3:** NOESY Spectra for Model RNAs P2, P3 and Termiantor-Model from the Cd1 Riboswitch.

**A** ^1^H/^1^H jump-and-return echo NOESY of RNA P2-Model at a concentration of 2,5 mM at 298K. The sample also contained 50 mM Bis-Tris Buffer, 120 mM NaCl and 10 mM MgCl_2_. The spectra were recorded with 4k/1k Time domain points, 84 scans and 25 ppm spectral width at 700 MHz.

**B** ^1^H/^1^H jump-and-return echo NOESY of RNA P3-Model at a concentration of 540 μM at 298K. The sample also contained 50 mM Bis-Tris Buffer, 120 mM NaCl and 10 mM MgCl_2_. The spectra were recorded with 4k/768 Time domain points, 272 scans and 24 ppm spectral width at 950 MHz.

**C** ^1^H/^1^H jump-and-return echo NOESY of RNA Terminator-Model at a concentration of 1 mM at 298K. The sample also contained 50 mM Bis-Tris Buffer, 110 mM NaCl and 8 mM MgCl_2_. The spectra were recorded with 2k/768 Time domain points, 56 scans and 25 ppm spectral width at 700 MHz.

**D** ^1^H/^1^H jump-and-return echo NOESY of RNA Cd1 full length at a concentration of 350 μM at 298K. The sample also contained 50 mM Bis-Tris Buffer, 120 mM NaCl and 10 mM MgCl_2_. The spectra were recorded with 2k/512 Time domain points, 272 scans and 24 ppm spectral width at 800 MHz. The NOESY of RNA Cd1 full length is overlayed withe the spectra from A-C.


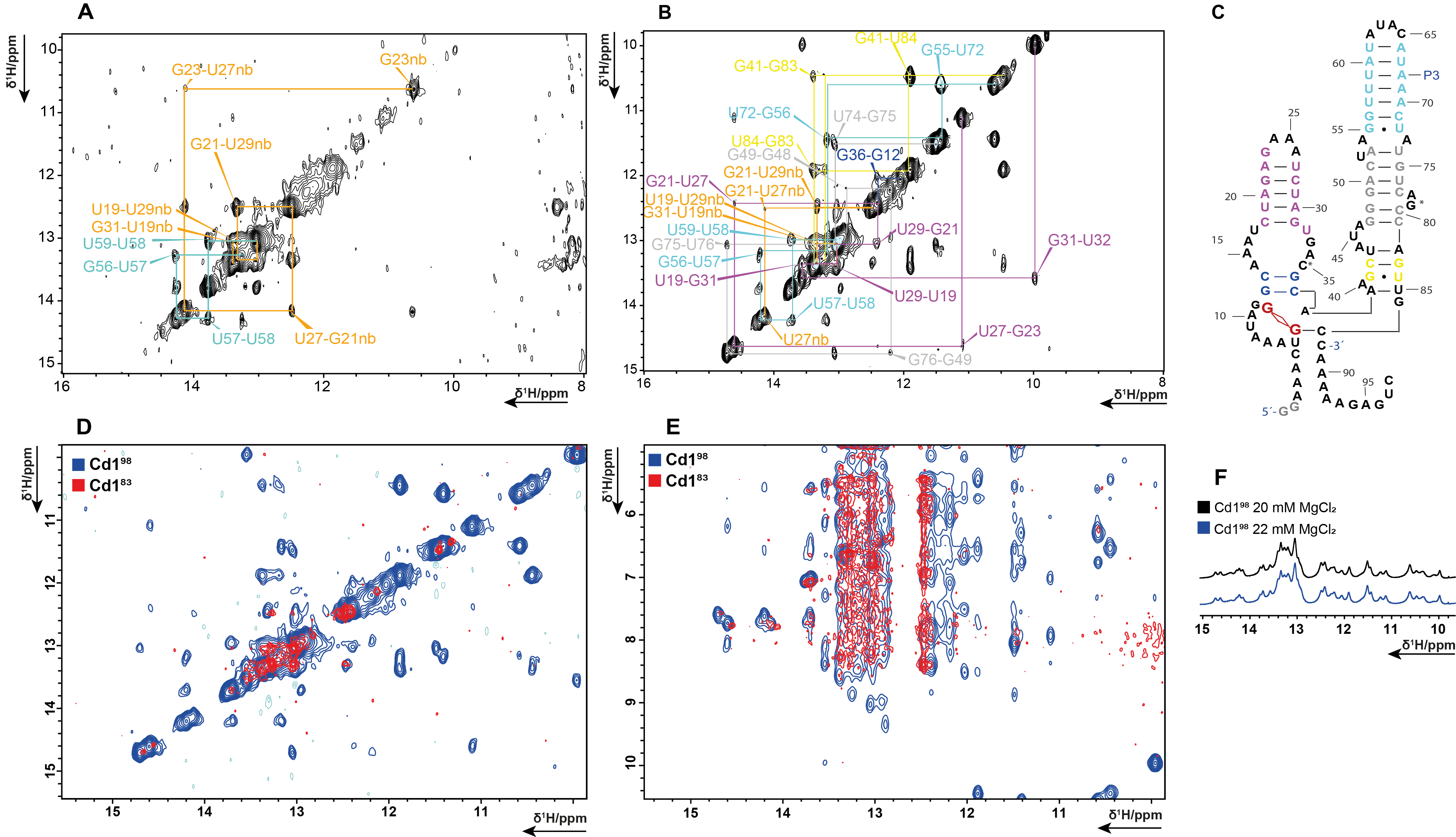


**SI Figure 4: (A)** ^1^H/^1^H jump-and-return echo NOESY of RNA Cd1^98^ at a concentration of 500 µM at 298K. The sample also contained 50 mM Bis-Tris Buffer, 120 mM NaCl and 20 mM MgCl_2_. The spectra were recorded with 2k/380 Time domain points, 384 scans and 24 ppm spectral width at 600 MHz. **(B)** ^1^H/^1^H jump-and-return echo NOESY of RNA Cd1^98^ at a concentration of 500 μM at 298K. The sample also contained 50 mM Bis-Tris Buffer, 120 mM NaCl, 22 mM MgCl_2_ and 500 μM c-di-GMP. The spectra were recorded with 2k/380 Time domain points, 368 scans and 24 ppm spectral width at 600 MHz. **(C)** Secondary Structure for Cd1^98^ with bound c-di-GMP. **(D and E)** Comparison of Cd1^98^ and Cd1^83^ ^1^H/^1^H jump-and-return echo NOESY Spectra. Cd1^98^ is the same spectra as in B. The Cd1^83^ ^1^H/^1^H jump-and-return echo NOESY was recorded at a concentration of 230 µM at 310 K. The sample also contained 50 mM Bis-Tris Buffer, 120 mM NaCl and 8 mM MgCl_2_. The spectra were recorded with 2k/512 Time domain points, 676 scans and 24 ppm spectral width at 600 MHz. **(F)** Comparison of the Imino region ^1^H spectra of Cd1^98^ with 20 and 22 mM MgCl_2_ recorded with 126 Scans at 600 MHz.


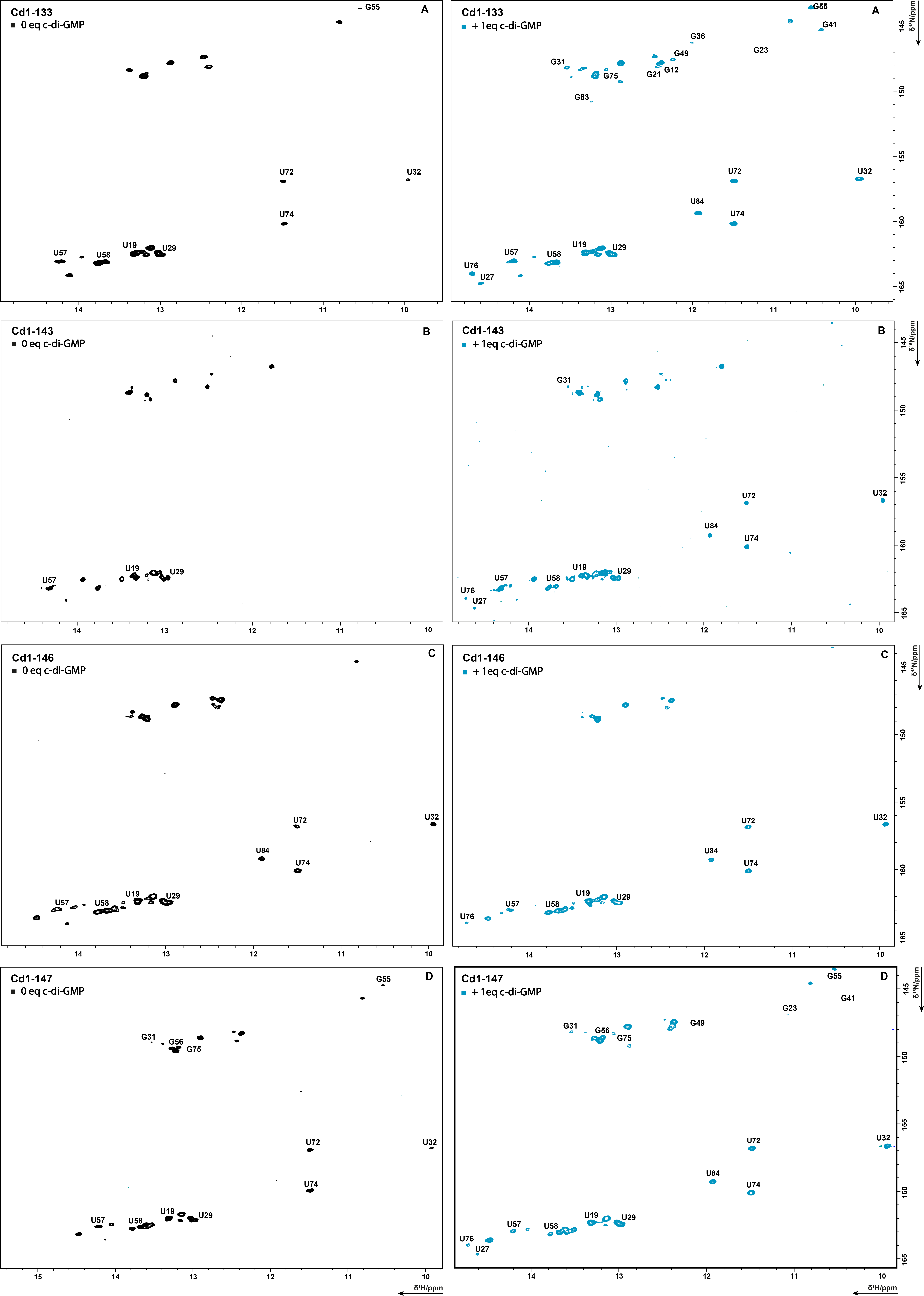


**
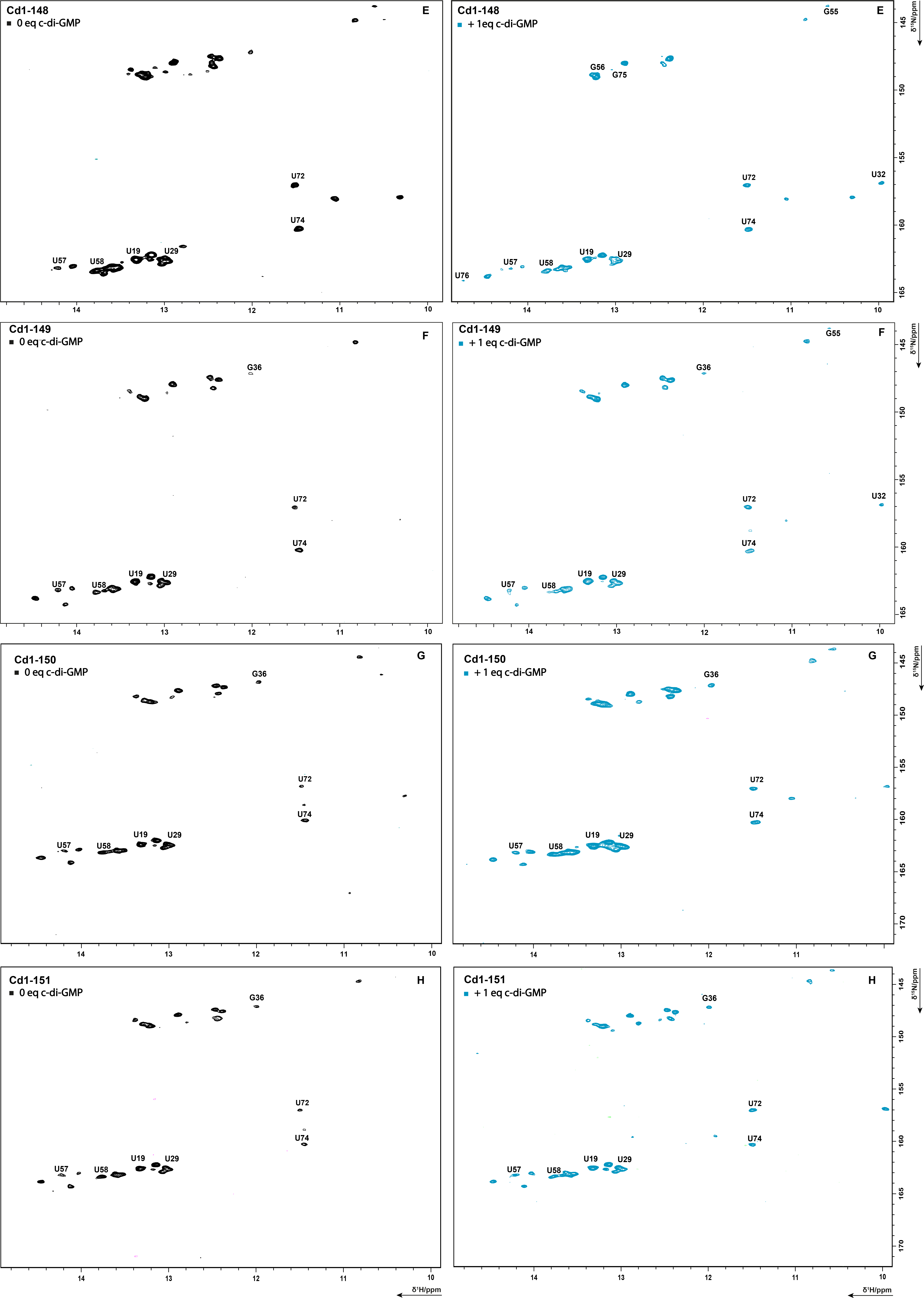
**

**SI Figure 5: (A)** 1H-2D ^15^N best TROSY NMR spectra of Cd1^133^ at a concentration of 200 μM at 298K. The sample also contained 50 mM Bis-Tris Buffer, 120 mM NaCl and 8 mM MgCl_2_. The spectra were recorded with 1k/256 Time domain points, 12 scans and 24 ppm spectral width at 700 MHz. Spectra in black represent Measurements with c-di-GMP in the sample and turquoise Measurements without. **(B)** ^15^N best TROSY NMR spectra of Cd1^143^ at a concentration of 200 μM at 298K. The sample also contained 50 mM Bis-Tris Buffer, 120 mM NaCl and 8 mM MgCl_2_. The spectra were recorded with 2k/256 Time domain points, 8 scans and 24 ppm spectral width at 800 MHz. Spectra in black represent Measurements with c-di-GMP in the sample and turquoise Measurements without. **(C)** 1H-2D ^15^N best TROSY NMR spectra of Cd1^146^ at a concentration of 200 μM at 298K. The sample also contained 50 mM Bis-Tris Buffer, 120 mM NaCl and 8 mM MgCl_2_. The spectra were recorded with 2k/256 Time domain points, 12 scans and 24 ppm spectral width at 800 MHz. Spectra in black represent Measurements with c-di-GMP in the sample and turquoise Measurements without. **(D)** 1H-2D ^15^N best TROSY NMR spectra of Cd1^147^ at a concentration of 200 μM at 298K. The sample also contained 50 mM Bis-Tris Buffer, 120 mM NaCl and 8 mM MgCl_2_. The spectra were recorded with 2k/256 Time domain points, 8 scans and 24 ppm spectral width at 800 MHz. Spectra in black represent Measurements with c-di-GMP in the sample and turquoise Measurements without. **(E)** 1H-2D ^15^N best TROSY NMR spectra of Cd1^148^ at a concentration of 190 μM at 298K. The sample also contained 50 mM Bis-Tris Buffer, 120 mM NaCl and 7,8 mM MgCl_2_. The spectra were recorded with 1k/256 Time domain points, 8 scans and 24 ppm spectral width at 600 MHz. Spectra in black represent Measurements with c-di-GMP in the sample and turquoise Measurements without. **(F)** 1H-2D ^15^N best TROSY NMR spectra of Cd1^149^ at a concentration of 200 μM at 298K. The sample also contained 50 mM Bis-Tris Buffer, 120 mM NaCl and 8 mM MgCl_2_. The spectra were recorded with 1k/256 Time domain points, 8 scans and 24 ppm spectral width at 600 MHz. Spectra in black represent Measurements with c-di-GMP in the sample and turquoise Measurements without. **(G)** 1H-2D ^15^N best TROSY NMR spectra of Cd1^150^ at a concentration of 200 μM at 298K. The sample also contained 50 mM Bis-Tris Buffer, 120 mM NaCl and 8 mM MgCl_2_. The spectra were recorded with 1k/256 Time domain points, 8 scans and 24 ppm spectral width at 600 MHz. Spectra in black represent Measurements with c-di-GMP in the sample and turquoise Measurements without. **(H)** 1H-2D ^15^N best TROSY NMR spectra of Cd1^151^ at a concentration of 200 μM at 298K. The sample also contained 50 mM Bis-Tris Buffer, 120 mM NaCl and 8 mM MgCl_2_. The spectra were recorded with 1k/256 Time domain points, 8 scans and 24 ppm spectral width at 600 MHz. Spectra in black represent Measurements with c-di-GMP in the sample and turquoise Measurements without.


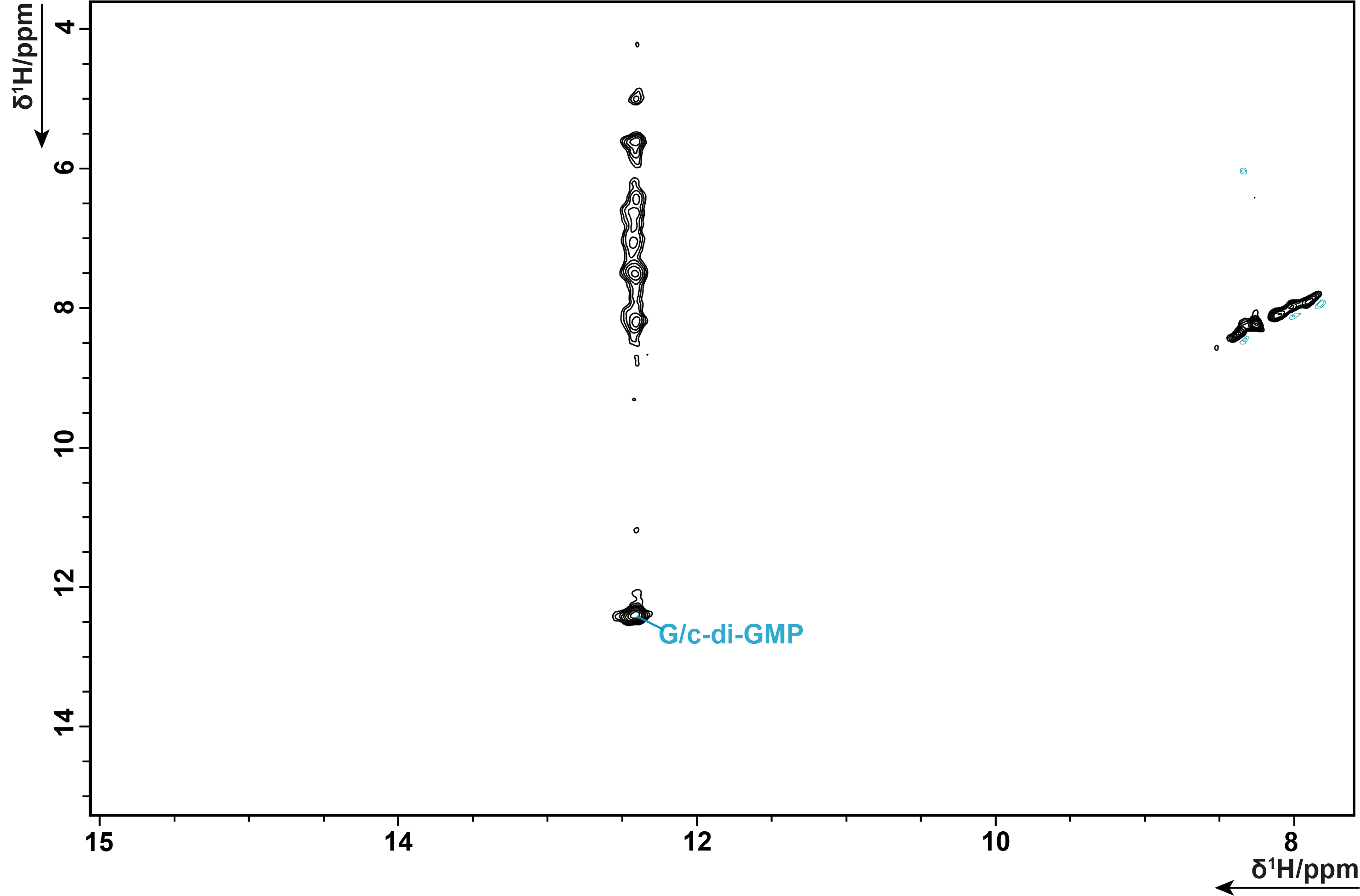


**SI Figure 6:** x-filter-NOESY Spectra for unlabeled Cd1^88^ with ^13^C/^15^N labeled c-di-GMP filtered to show ^1^H bound to ^15^N. RNA Cd1^88^ is present at a concentration of 200 µM. The sample also contained 50 mM Bis-Tris Buffer, 120 mM NaCl and 8 mM MgCl_2_ and 300 µM ^13^C/^15^N labeled c-di-GMP. The spectra were recorded with 2k/256 Time domain points, 768 scans and 24 ppm spectral width at 800 MHz at 281K.


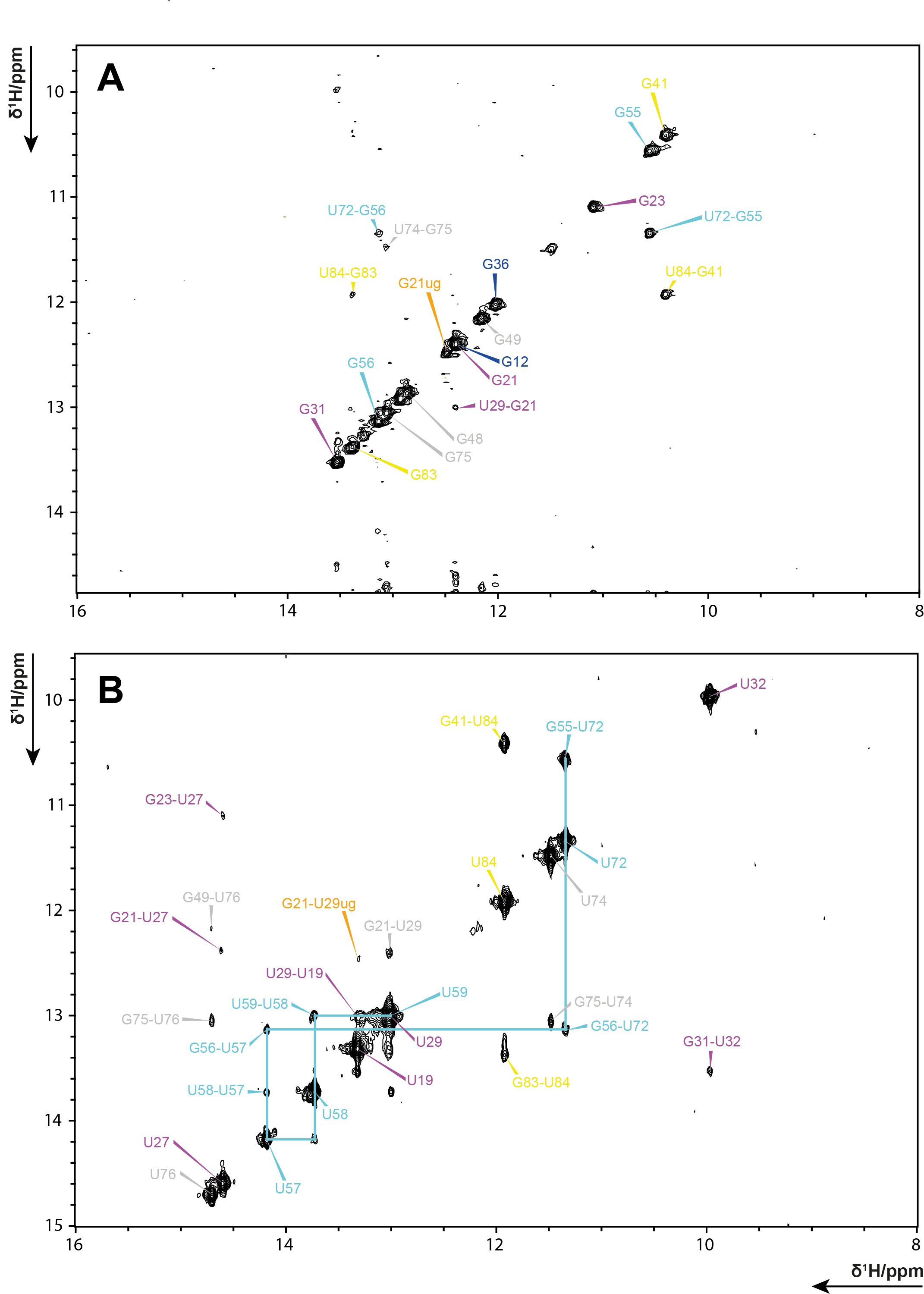


**SI Figure 7: A)** x-filter-NOESY Spectra for ^15^N G labeled Cd1^98^ was filtered to show ^1^H bound to ^15^N. RNA Cd1^98^ is present at a concentration of 130 µM. The sample also contained 50 mM Bis-Tris Buffer, 110 mM NaCl and 8 mM MgCl_2_ and 130 µM c-di-GMP. The spectra were recorded with 2688/512 Time domain points, 520 scans and 24 ppm spectral width at 800 MHz at 298K. **B)** x-filter-NOESY Spectra for ^15^N U labeled Cd1^98^ was filtered to show ^1^H bound to ^15^N. RNA Cd1^98^ is present at a concentration of 160 µM. The sample also contained 50 mM Bis-Tris Buffer, 110 mM NaCl and 8 mM MgCl_2_ and 160 µM c-di-GMP. The spectra were recorded with 4k/512 Time domain points, 368 scans and 24 ppm spectral width at 700 MHz at 298 K.

**SI Table 3:** Peak List of the resonance assignments for the Cd1 Riboswitch

| **Assignment** | **w1/1H ppm** | **w2/1H ppm** | **Assignment** | **15N ppm** | **1H ppm** |
| --- | --- | --- | --- | --- | --- |
|  |  |  |  |  |  |
| **G31H1-H1** | 13.317 | 13.313 | **U19N3-H3** | 161.850 | 13.314 |
| **G31H1-U32H3** | 13.532 | 9.984 | **G23N1-H1** | 146.439 | 11.075 |
| **U32H3-H3** | 9.985 | 9.983 | **U27N3-H3** | 164.233 | 14.597 |
| **G36H1-G12H1** | 12.047 | 12.405 | **U29N3-H3** | 161.850 | 13.017 |
| **G36H1-H1** | 12.044 | 12.043 | **G31N1-H1** | 147.664 | 13.543 |
| **G41H1-H1** | 10.408 | 10.407 | **U32N3-H3** | 156.201 | 9.953 |
| **G41H1-G83H1** | 10.407 | 13.334 | **G36N1-H1** | 145.729 | 12.011 |
| **G41H1-U84H3** | 10.411 | 11.934 | **G41N1-H1** | 144.759 | 10.417 |
| **G48H1-H1** | 12.885 | 12.882 | **G49N1-H1** | 147.062 | 12.241 |
| **G49H1-G48H1** | 12.202 | 12.878 | **G55N1-H1** | 143.074 | 10.546 |
| **G49H1-H1** | 12.201 | 12.197 | **G56N1-H1** | 148.202 | 13.162 |
| **A50H2-U74H3** | 8.041 | 11.477 | **U57N3-H3** | 162.478 | 14.193 |
| **A50H2-U76H3** | 7.584 | 14.689 | **U58N3-H3** | 162.583 | 13.673 |
| **G55H1-H1** | 10.545 | 10.546 | **U72N3-H3** | 156.367 | 11.482 |
| **G55H1-U72H3** | 10.549 | 11.415 | **U74N3-H3** | 159.656 | 11.493 |
| **G56H1-H1** | 13.165 | 13.160 | **G75N1-H1** | 147.863 | 13.058 |
| **G56H1-U57H3** | 13.158 | 14.177 | **U76N3-H3** | 163.496 | 14.705 |
| **U57H3-H3** | 14.185 | 14.163 | **G83N1-H1** | 150.273 | 13.241 |
| **U57H3-U58H3** | 14.153 | 13.684 | **U84N3-H3** | 158.830 | 11.928 |
| **U58H3-H3** | 13.685 | 13.684 |  |  |  |
| **U59H3-U58H3** | 12.973 | 13.686 |  |  |  |
| **U59H3-H3** | 12.980 | 12.970 |  |  |  |
| **A68H2-U59H3** | 6.395 | 12.972 |  |  |  |
| **A69H2-U58H3** | 7.035 | 13.683 |  |  |  |
| **A70H2-U57H3** | 7.608 | 14.172 |  |  |  |
| **A70H2-U58H3** | 7.631 | 13.682 |  |  |  |
| **U72H3-G56H1** | 11.422 | 13.154 |  |  |  |
| **U72H3-H3** | 11.421 | 11.418 |  |  |  |
| **U74H3-H3** | 11.481 | 11.480 |  |  |  |
| **U74H3-G75H1** | 11.480 | 13.059 |  |  |  |
| **G75H1-H1** | 13.061 | 13.057 |  |  |  |
| **G75H1-U76H3** | 13.065 | 14.691 |  |  |  |
| **U76H3-G49H1** | 14.692 | 12.203 |  |  |  |
| **U76H3-H3** | 14.692 | 14.689 |  |  |  |
| **U84H3-G83H1** | 11.933 | 13.340 |  |  |  |
| **U84H3-H3** | 11.935 | 11.933 |  |  |  |

**SI Table 4:** Peak List of the resonance assignments for the *apo* form of pilM Riboswitch found in Fig. 7 A, B and C

| **Assignment 7A** | **w1/1H ppm** | **w2/1H ppm** | **Assignment 7B** | **w1/1H ppm** | **w2/1H ppm** | **Assignment 7C** | **w1/1H ppm** | **w2/1H ppm** |
| --- | --- | --- | --- | --- | --- | --- | --- | --- |
| **U15H3-G38H1** | 10,807 | 11,032 | **U15H3-G38H1** | 10,183 | 10,775 | **U15H3-H3** | 10.766 | 10.771 |
| **U15H3-G39H1** | 10,799 | 12,927 | **U15H3-G39H1** | 10,181 | 12,196 | **U15H3-G38H1** | 10.77 | 11.037 |
| **U15H3-H3** | 10,805 | 10,805 | **U15H3-H3** | 10,182 | 10,188 | **U22H3-H3** | 11.872 | 11.875 |
| **U22H3-G30H1** | 11,854 | 12,885 | **U22H3-G30H1** | 11,761 | 12,809 | **U22H3-G30H1** | 11.876 | 12.901 |
| **U22H3-G31H1** | 11,859 | 11,355 | **U22H3-G31H1** | 11,767 | 11,361 | **U22H3-G31H1** | 11.873 | 11.37 |
| **U22H3-H3** | 11,855 | 11,855 | **U22H3-H3** | 11,762 | 11,768 | **U22H3-U32H3** | 11.872 | 13.601 |
| **U22H3-U32H3** | 11,854 | 13,593 | **U22H3-U32H3** | 11,761 | 13,616 | **G25H1-H1** | 12.247 | 12.246 |
| **G25H1-H1** | 12,149 | 12,155 | **G25H1-H1** | 12,204 | 12,206 | **A28H2-H2** | 9.485 | 9.488 |
| **G27H1-H1** | 9,553 | 9,557 | **A28H2-G62H1** | 9,449 | 12,929 | **A28H2-G62H1** | 9.48 | 12.903 |
| **A28H2-G52H1** | 9,489 | 12,902 | **A28H2-H2** | 9,444 | 9,446 | **A28H2-A63H8** | 9.485 | 8.706 |
| **A28H2-H2** | 9,489 | 9,490 | **A28H2-U55H3** | 9,423 | 14,016 | **G29H1-H1** | 12.37 | 12.372 |
| **A28H2-U55H3** | 9,510 | 14,010 | **G29H1-G25H1** | 12,358 | 12,203 | **G29H1-G30H1** | 12.364 | 12.902 |
| **G29H1-G30H1** | 12,369 | 12,885 | **G29H1-G25H1** | 12,210 | 12,331 | **G30H1-U22H3** | 12.901 | 11.878 |
| **G29H1-H1** | 12,366 | 12,364 | **G29H1-G30H1** | 12,353 | 12,806 | **G30H1-G29H1** | 12.904 | 12.37 |
| **G30H1-G29H1** | 12,892 | 12,362 | **G29H1-H1** | 12,340 | 12,348 | **G30H1-H1** | 12.895 | 12.901 |
| **G30H1-G31H1** | 12,884 | 11,357 | **G30H1-G29H1** | 12,836 | 12,363 | **G30H1-G31H1** | 12.907 | 11.373 |
| **G30H1-H1** | 12,894 | 12,895 | **G30H1-G31H1** | 12,825 | 11,352 | **G31H1-U22H3** | 11.367 | 11.877 |
| **G30H1-U22H3** | 12,887 | 11,860 | **G30H1-H1** | 12,807 | 12,812 | **G31H1-G30H1** | 11.371 | 12.904 |
| **G31H1-G30H1** | 11,342 | 12,887 | **G30H1-U22H3** | 12,808 | 11,768 | **G31H1-H1** | 11.366 | 11.37 |
| **G31H1-H1** | 11,353 | 11,355 | **G31H1-G30H1** | 11,363 | 12,819 | **G31H1-U32H3** | 11.37 | 13.603 |
| **G31H1-U22H3** | 11,353 | 11,859 | **G31H1-G33H1** | 11,339 | 11,834 | **U32H3-U22H3** | 13.6 | 11.877 |
| **G31H1-U32H3** | 11,350 | 13,593 | **G31H1-H1** | 11,354 | 11,359 | **U32H3-G31H1** | 13.598 | 11.367 |
| **U32H3-G31H1** | 13,593 | 11,354 | **G31H1-U22H3** | 11,357 | 11,768 | **U32H3-H3** | 13.601 | 13.602 |
| **U32H3-H3** | 13,592 | 13,592 | **G31H1-U32H3** | 11,354 | 13,613 | **G33H1-G34H1** | 11.874 | 13.205 |
| **U32H3-U22H3** | 13,589 | 11,857 | **U32H3-G31H1** | 13,605 | 11,359 | **G34H1-G33H1** | 13.196 | 11.873 |
| **G33H1-G34H1** | 11,849 | 13,192 | **U32H3-G33H1** | 13,606 | 11,884 | **G34H1-H1** | 13.208 | 13.209 |
| **G33H1-H1** | 11,855 | 11,855 | **U32H3-H3** | 13,612 | 13,614 | **G35H1-G34H1** | 10.953 | 13.212 |
| **G34H1-G33H1** | 13,191 | 11,853 | **U32H3-U22H3** | 13,611 | 11,768 | **G35H1-H1** | 10.94 | 10.947 |
| **G34H1-G35H1** | 13,179 | 10,944 | **G33H1-G31H1** | 11,843 | 11,332 | **C37H41-G66H1** | 9.031 | 13.165 |
| **G34H1-H1** | 13,188 | 13,192 | **G33H1-G34H1** | 11,890 | 13,217 | **G38H1-U15H3** | 11.037 | 10.771 |
| **G35H1-G34H1** | 10,935 | 13,193 | **G33H1-H1** | 11,866 | 11,881 | **G38H1-H1** | 11.037 | 11.043 |
| **G35H1-H1** | 10,933 | 10,937 | **G34H1-G33H1** | 13,200 | 11,886 | **U46H3-H3** | 10.326 | 10.327 |
| **C37H41-G38H1** | 9,084 | 11,028 | **G34H1-G35H1** | 13,201 | 11,132 | **G50H1-H1** | 12.64 | 12.641 |
| **C37H41-G66H1** | 9,091 | 13,170 | **G34H1-H1** | 13,207 | 13,213 | **G50H1-G51H1** | 12.632 | 13.016 |
| **C37H41-H41** | 9,079 | 9,086 | **G35H1-G34H1** | 11,144 | 13,220 | **G51H1-G50H1** | 13 | 12.643 |
| **C37H41-U15H3** | 9,092 | 10,804 | **G35H1-H1** | 11,131 | 11,135 | **G51H1-H1** | 13.018 | 13.018 |
| **G38H1-C37H41** | 11,031 | 9,108 | **G38H1-G39H1** | 10,779 | 12,200 | **G51H1-G52H1** | 13.031 | 12.892 |
| **G38H1-G39H1** | 11,021 | 12,930 | **G38H1-G66H1** | 10,772 | 13,095 | **G52H1-G51H1** | 12.912 | 13.022 |
| **G38H1-G66H1** | 11,003 | 13,161 | **G38H1-H1** | 10,776 | 10,780 | **G52H1-H1** | 12.902 | 12.915 |
| **G38H1-H1** | 11,030 | 11,032 | **G38H1-U15H3** | 10,776 | 10,189 | **G52H1-U53H3** | 12.898 | 14.781 |
| **G38H1-U15H3** | 11,033 | 10,807 | **G39H1-G38H1** | 12,181 | 10,780 | **U53H3-G52H1** | 14.777 | 12.898 |
| **G39H1-G38H1** | 12,915 | 11,031 | **G39H1-H1** | 12,204 | 12,206 | **U53H3-H3** | 14.779 | 14.78 |
| **G39H1-U15H3** | 12,931 | 10,805 | **G39H1-U15H3** | 12,198 | 10,184 | **U53H3-G62H1** | 14.778 | 12.9 |
| **U46H3-G69H1** | 10,294 | 9,154 | **U46H3-G69H1** | 10,344 | 9,116 | **U55H3-A28H2** | 14.006 | 9.48 |
| **U46H3-G70H1** | 10,288 | 13,105 | **U46H3-G70H1** | 10,356 | 13,076 | **U55H3-H3** | 14.004 | 14.005 |
| **U46H3-H3** | 10,294 | 10,295 | **U46H3-H3** | 10,334 | 10,342 | **U55H3-G60H1** | 13.999 | 12.391 |
| **G50H1-G51H1** | 12,661 | 13,022 | **G50H1-G51H1** | 12,628 | 13,041 | **U55H3-G62H1** | 14.004 | 12.905 |
| **G50H1-H1** | 12,664 | 12,664 | **G50H1-H1** | 12,629 | 12,634 | **G60H1-U55H3** | 12.39 | 14.007 |
| **G51H1-G50H1** | 13,022 | 12,667 | **G51H1-G50H1** | 13,023 | 12,636 | **G60H1-H1** | 12.392 | 12.388 |
| **G51H1-H1** | 13,026 | 13,026 | **G51H1-H1** | 13,028 | 13,034 | **G62H1-A28H2** | 12.899 | 9.483 |
| **G52H1-A28H2** | 12,905 | 9,490 | **G52H1-H1** | 12,929 | 12,925 | **G62H1-U53H3** | 12.896 | 14.779 |
| **G52H1-H1** | 12,894 | 12,895 | **G52H1-U53H3** | 12,827 | 14,824 | **G62H1-U55H3** | 12.892 | 13.991 |
| **G52H1-U53H3** | 12,891 | 14,775 | **U53H3-G52H1** | 14,814 | 12,897 | **G62H1-H1** | 12.902 | 12.904 |
| **U53H3-G52H1** | 14,777 | 12,900 | **U53H3-G62H1** | 14,799 | 12,907 | **G69H1-U46H3** | 9.179 | 10.313 |
| **U53H3-G62H1** | 14,777 | 12,900 | **U53H3-H3** | 14,822 | 14,823 | **G69H1-H1** | 9.182 | 9.187 |
| **U53H3-G62H1** | 14,777 | 12,900 | **U55H3-A28H2** | 14,014 | 9,439 | **G70H1-H1** | 13.055 | 13.063 |
| **U53H3-H3** | 14,766 | 14,775 | **U55H3-G60H1** | 14,009 | 12,401 | **G70H1-G71H1** | 13.063 | 12.45 |
| **U53H3-H3** | 14,797 | 14,785 | **U55H3-G62H1** | 14,019 | 12,929 | **G71H1-G70H1** | 12.45 | 13.059 |
| **U55H3-A28H2** | 13,998 | 9,490 | **U55H3-H3** | 14,013 | 14,016 | **G71H1-H1** | 12.448 | 12.449 |
| **U55H3-G60H1** | 13,995 | 12,385 | **G60H1-H1** | 12,391 | 12,400 |  |  |  |
| **U55H3-G62H1** | 13,999 | 12,903 | **G60H1-U55H3** | 12,401 | 14,015 |  |  |  |
| **U55H3-H3** | 13,999 | 13,999 | **G62H1-A28H2** | 12,926 | 9,448 |  |  |  |
| **G60H1-H1** | 12,366 | 12,381 | **G62H1-H1** | 12,925 | 12,930 |  |  |  |
| **G60H1-U55H3** | 12,381 | 13,993 | **G62H1-U53H3** | 12,827 | 14,824 |  |  |  |
| **G62H1-H1** | 12,894 | 12,895 | **G62H1-U55H3** | 12,923 | 14,011 |  |  |  |
| **G62H1-U53H3** | 12,891 | 14,775 | **G66H1-G38H1** | 13,089 | 10,793 |  |  |  |
| **G62H1-U55H3** | 12,900 | 14,000 | **G66H1-H1** | 13,086 | 13,084 |  |  |  |
| **G66H1-C37H41** | 13,159 | 9,107 | **G69H1-G70H1** | 9,065 | 13,033 |  |  |  |
| **G66H1-G38H1** | 13,166 | 11,034 | **G69H1-H1** | 9,098 | 9,109 |  |  |  |
| **G66H1-H1** | 13,164 | 13,159 | **G69H1-U46H3** | 9,094 | 10,340 |  |  |  |
| **G69H1-H1** | 9,119 | 9,128 | **G70H1-G69H1** | 13,060 | 9,089 |  |  |  |
| **G69H1-U46H3** | 9,143 | 10,296 | **G70H1-G71H1** | 13,066 | 12,476 |  |  |  |
| **G70H1-G71H1** | 13,102 | 12,448 | **G70H1-H1** | 13,055 | 13,055 |  |  |  |
| **G70H1-H1** | 13,104 | 13,106 | **G70H1-U46H3** | 13,052 | 10,342 |  |  |  |
| **G70H1-U46H3** | 13,096 | 10,293 | **G71H1-G70H1** | 12,452 | 13,074 |  |  |  |
| **G71H1-G70H1** | 12,442 | 13,102 | **G71H1-H1** | 12,466 | 12,473 |  |  |  |
| **G71H1-H1** | 12,442 | 12,446 |  |  |  |  |  |  |


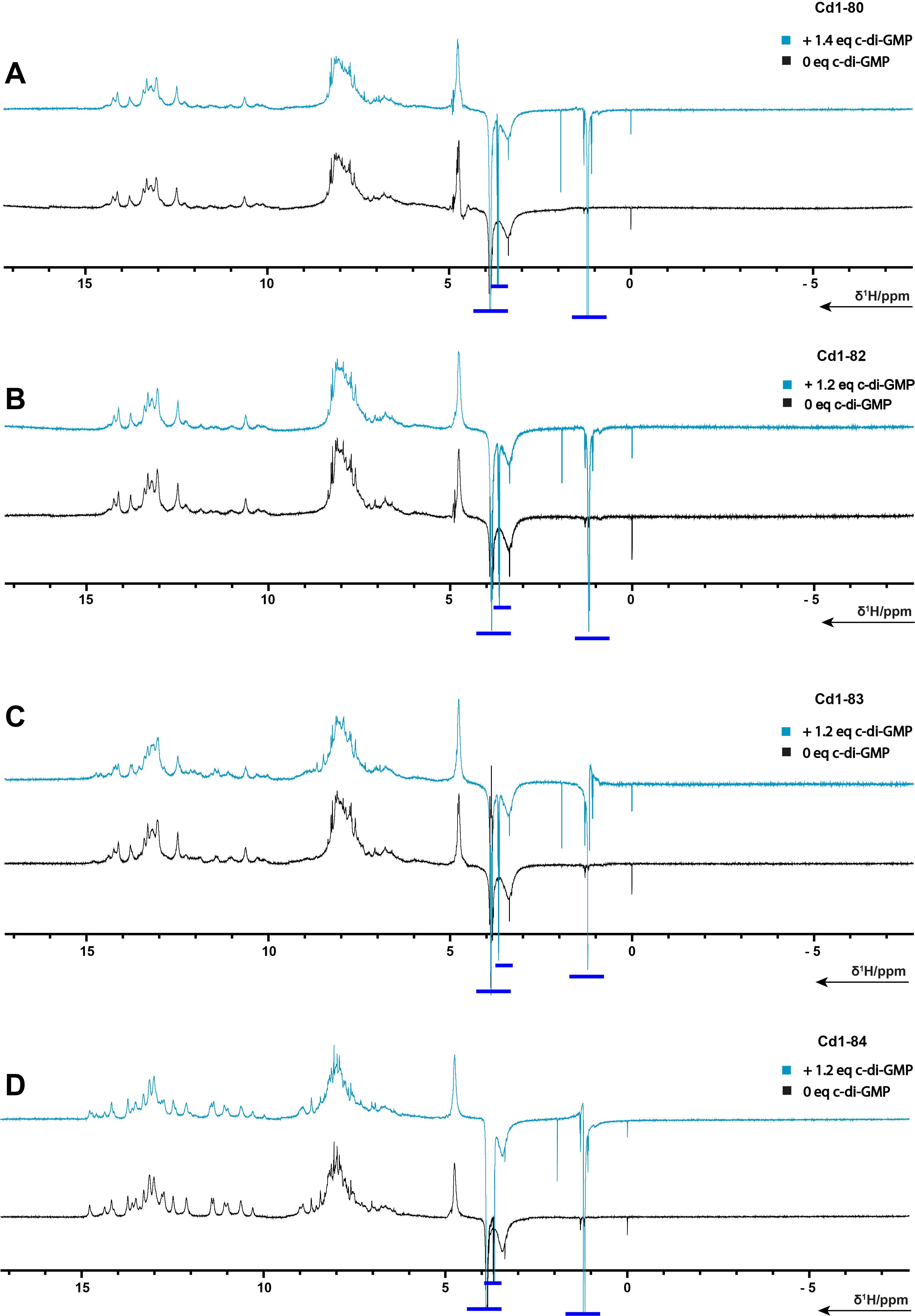


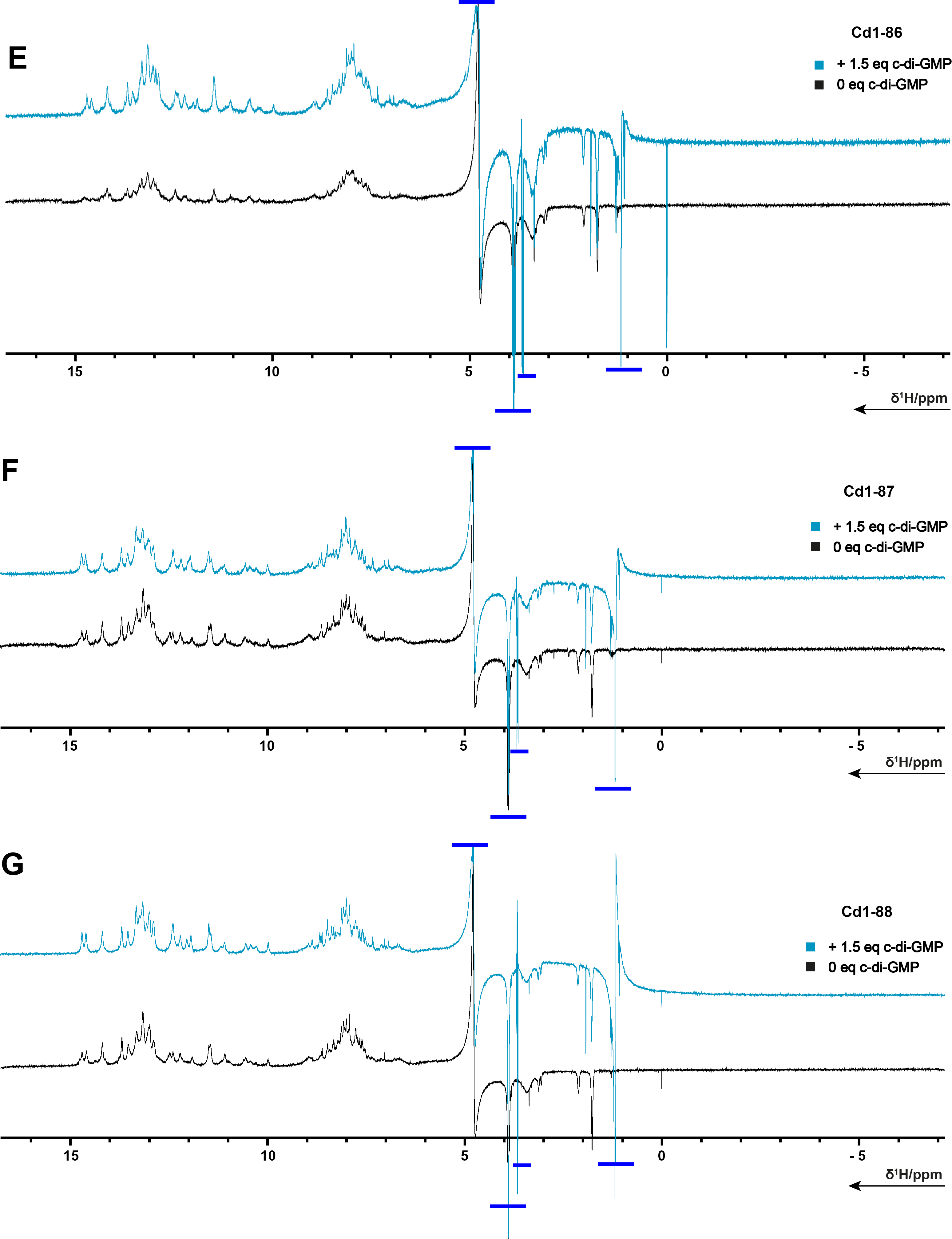


**SI Figure 8: (A)** ^1^H-1D jump-and-return echo NMR spectra of Cd1^80^ at a concentration of 200 μM at 298K. The sample also contained 50 mM Bis-Tris Buffer, 120 mM NaCl and 8mM MgCl_2_. The spectra were recorded with 16k Time domain points, 256 scans and 25 ppm spectral width at 600 MHz**. (B)** ^1^H-1D jump-and-return echo NMR spectra of Cd1^82^ at a concentration of 200 μM at 298K. The sample also contained 50 mM Bis-Tris Buffer, 120 mM NaCl and 8mM MgCl_2_. The spectra were recorded with 16k Time domain points, 256 scans and 25 ppm spectral width at 600 MHz**. (C)** ^1^H-1D jump-and-return echo NMR spectra of Cd1^83^ at a concentration of 200 μM at 298K. The sample also contained 50 mM Bis-Tris Buffer, 120 mM NaCl and 8mM MgCl_2_. The spectra were recorded with 16k Time domain points, 256 scans and 25 ppm spectral width at 600 MHz. **(D)** ^1^H-1D jump-and-return echo NMR spectra of Cd1^84^ at a concentration of 200 μM at 298K. The sample also contained 50 mM Bis-Tris Buffer, 120 mM NaCl and 8mM MgCl_2_. The spectra were recorded with 16k Time domain points, 256 scans and 25 ppm spectral width at 600 MHz**. (E)** ^1^H-1D jump-and-return echo NMR spectra of Cd1^86^ at a concentration of 70 μM at 298K. The sample also contained 50 mM Bis-Tris Buffer, 110 mM NaCl and 2.8 mM MgCl_2_. The spectra were recorded with 16k Time domain points, 256 scans and 24 ppm spectral width at 599 MHz**. (F)** ^1^H-1D jump-and-return echo NMR spectra of Cd1^87^ at a concentration of 200 μM at 298K. The sample also contained 50 mM Bis-Tris Buffer, 110 mM NaCl and 8 mM MgCl_2_. The spectra were recorded with 16k Time domain points, 128 scans and 24 ppm spectral width at 599 MHz. **(G)** ^1^H-1D jump-and-return echo NMR spectra of Cd1^88^ at a concentration of 200 μM at 298K. The sample also contained 50 mM Bis-Tris Buffer, 110 mM NaCl and 8 mM MgCl_2_. The spectra were recorded with 16k Time domain points, 256 scans and 24 ppm spectral width at 599 MHz. Spectra in black represent Measurements with c-di-GMP in the sample and turquoise Measurements without.


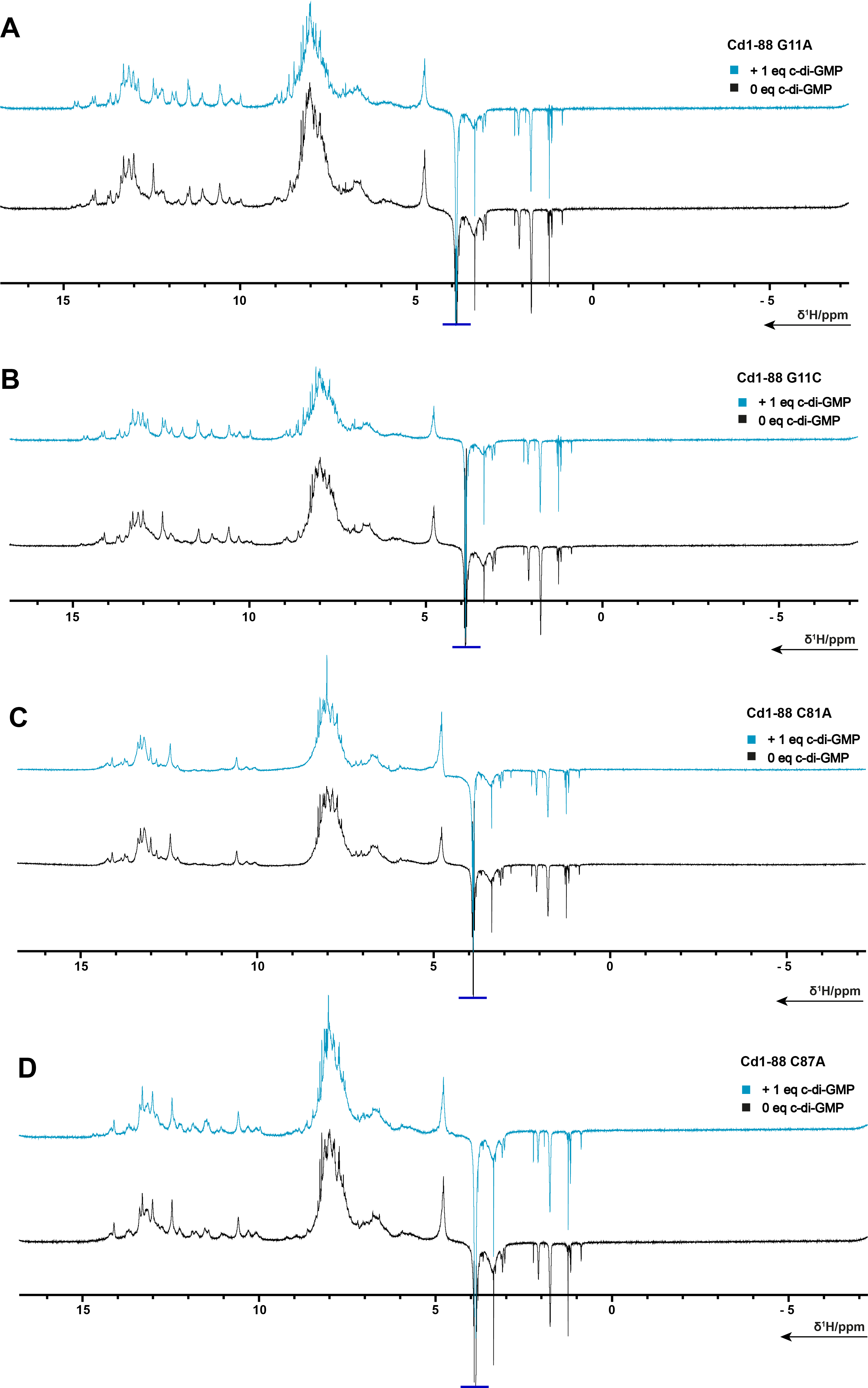


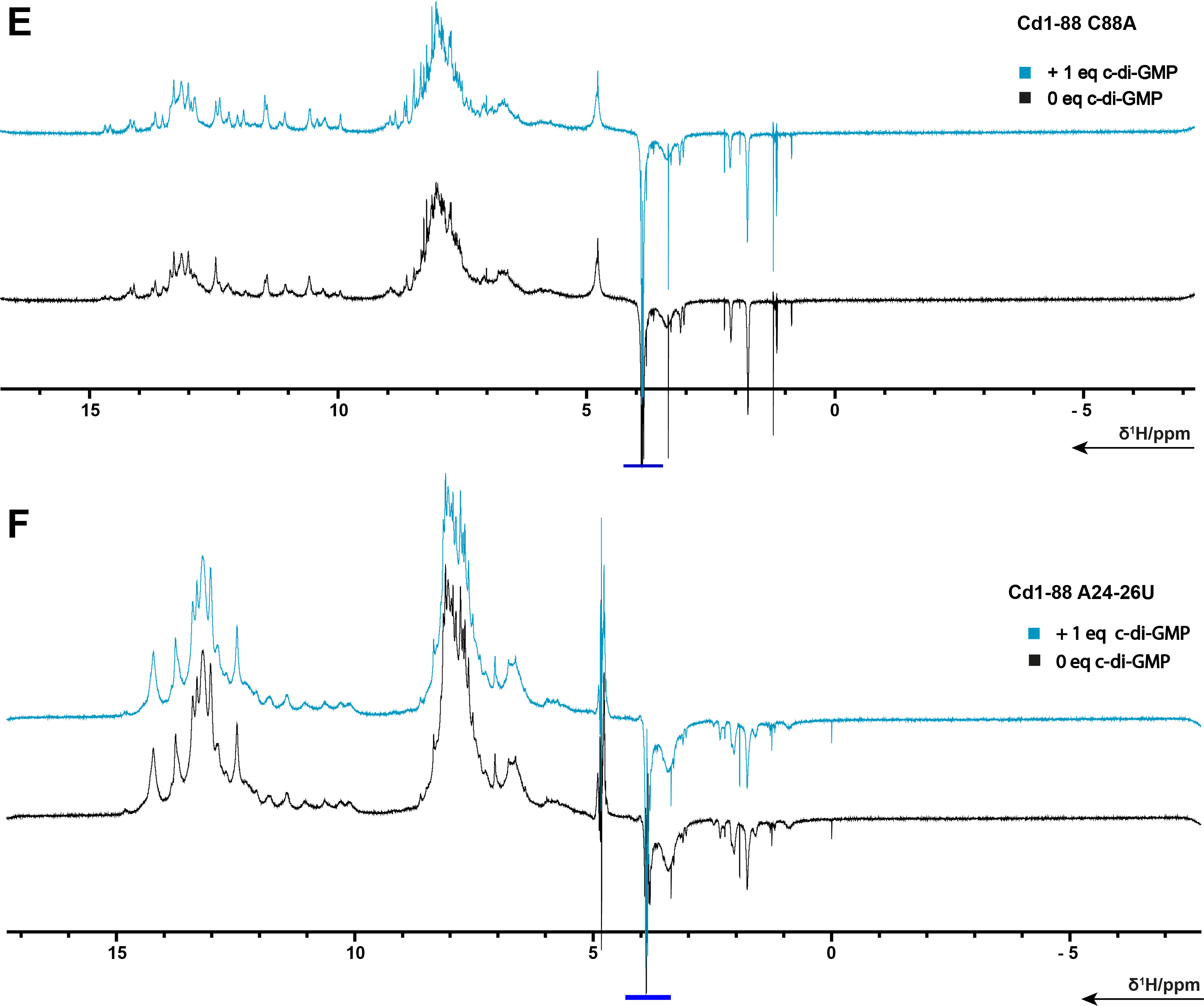


**SI Figure 9: (A)** ^1^H-1D jump-and-return echo NMR spectra of Cd1-88C11A at a concentration of 200 μM at 298K. The sample also contained 50 mM Bis-Tris Buffer, 110 mM NaCl and 8 mM MgCl_2_. The spectra were recorded with 16k Time domain points, 256 scans and 24 ppm spectral width at 800 MHz. **(B)** ^1^H-1D jump-and-return echo NMR spectra of Cd1-88 C11G at a concentration of 200 μM at 298K. The sample also contained 50 mM Bis-Tris Buffer, 110 mM NaCl and 8 mM MgCl_2_. The spectra were recorded with 16k Time domain points, 256 scans and 24 ppm spectral width at 800 MHz. **(C)** ^1^H-1D jump-and-return echo NMR spectra of Cd1-88 C81A at a concentration of 200 μM at 298K. The sample also contained 50 mM Bis-Tris Buffer, 110 mM NaCl and 8 mM MgCl_2_. The spectra were recorded with 16k Time domain points, 256 scans and 24 ppm spectral width at 800MHz. **(D)** ^1^H-1D jump-and-return echo NMR spectra of Cd1-88 C87A at a concentration of 200 μM at 298K. The sample also contained 50 mM Bis-Tris Buffer, 110 mM NaCl and 8 mM MgCl_2_. The spectra were recorded with 16k Time domain points, 256 scans and 24 ppm spectral width at 800 MHz. **(E)** ^1^H-1D jump-and-return echo NMR spectra of Cd1-88 C88A at a concentration of 200 μM at 298K. The sample also contained 50 mM Bis-Tris Buffer, 110 mM NaCl and 8 mM MgCl_2_. The spectra were recorded with 16k Time domain points, 256 scans and 24 ppm spectral width at 800 MHz. **(F)** ^1^H-1D jump-and-return echo NMR spectra of Cd1-88 A24-26U at a concentration of 500 μM at 298K. The sample also contained 50 mM Bis-Tris Buffer, 120 mM NaCl and 8 mM MgCl_2_. The spectra were recorded with 16k Time domain points, 256 scans and 25 ppm spectral width at 700 MHz. Spectra in black represent Measurements with c-di-GMP in the sample and turquoise Measurements without.


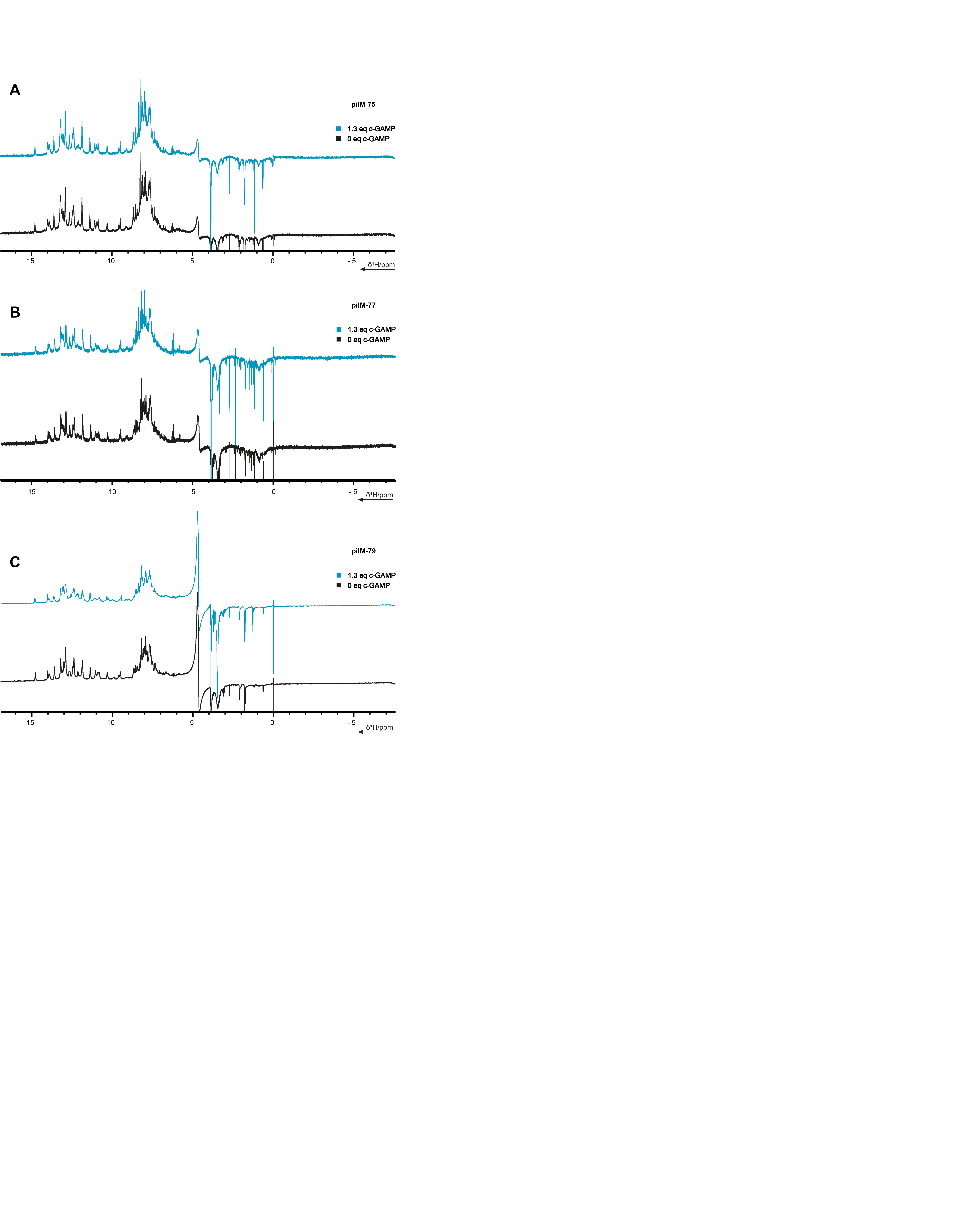


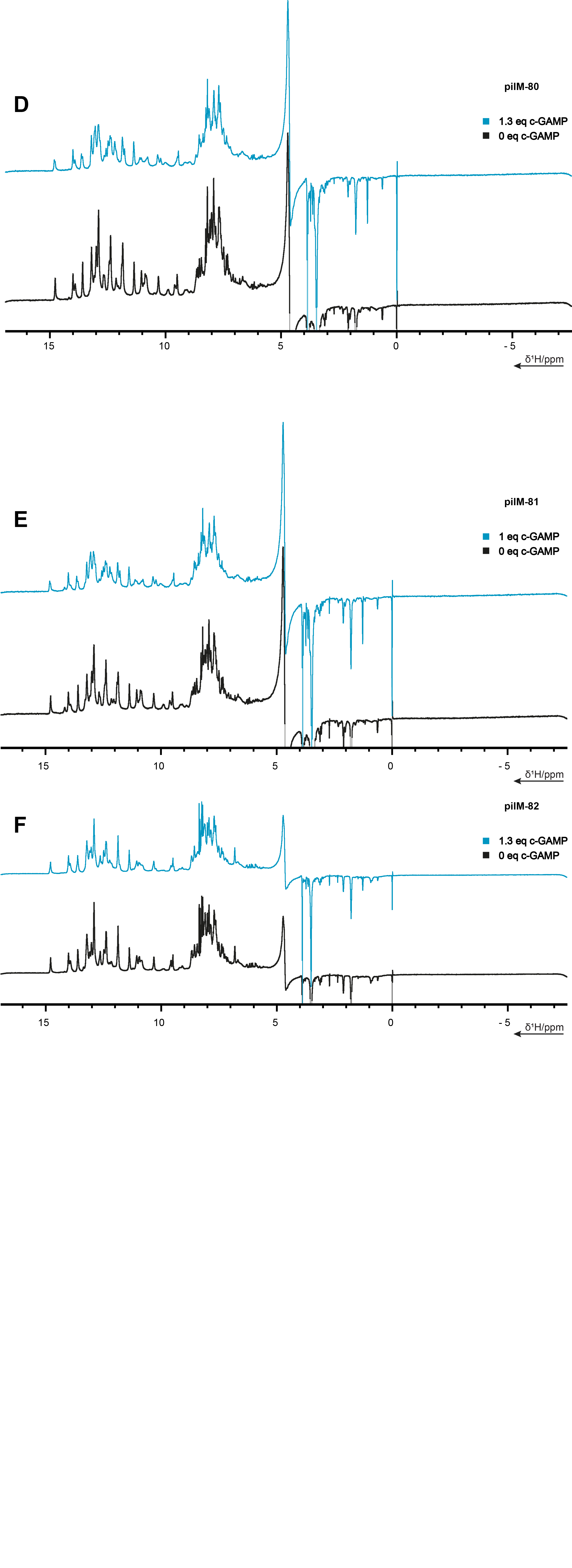


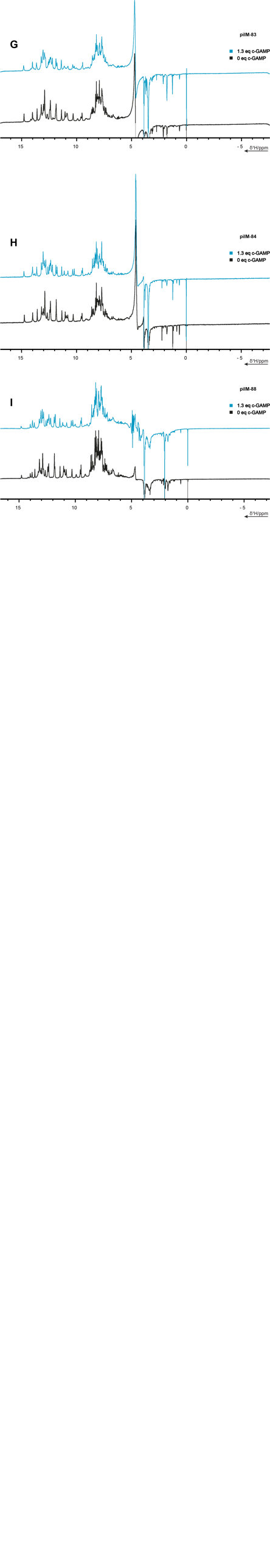


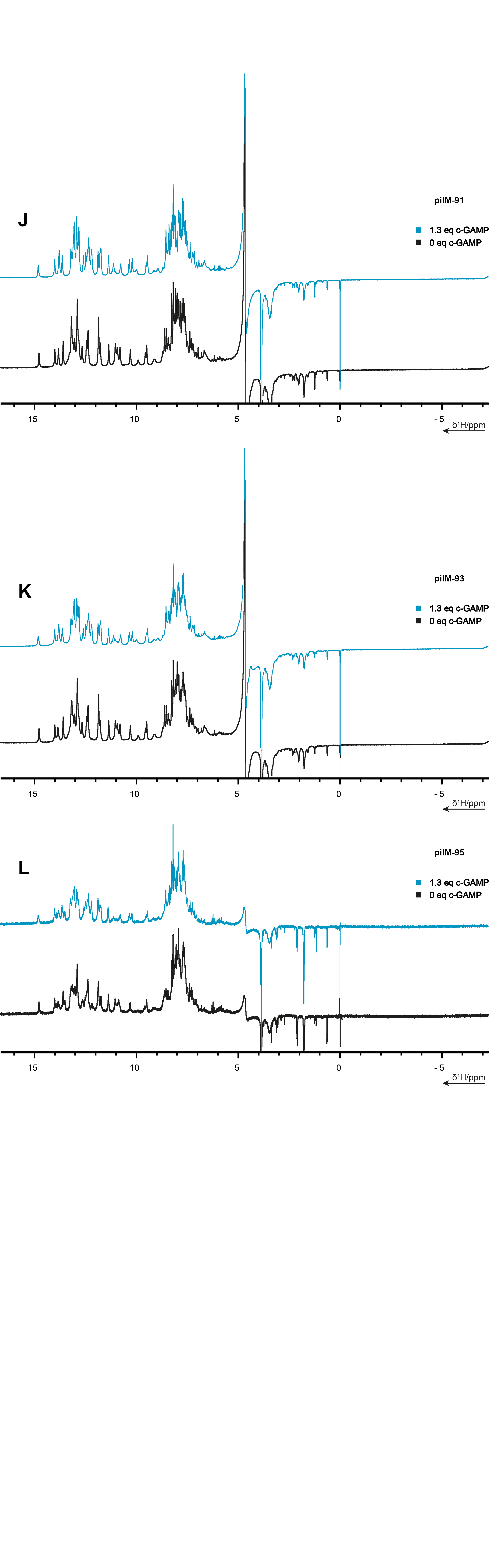


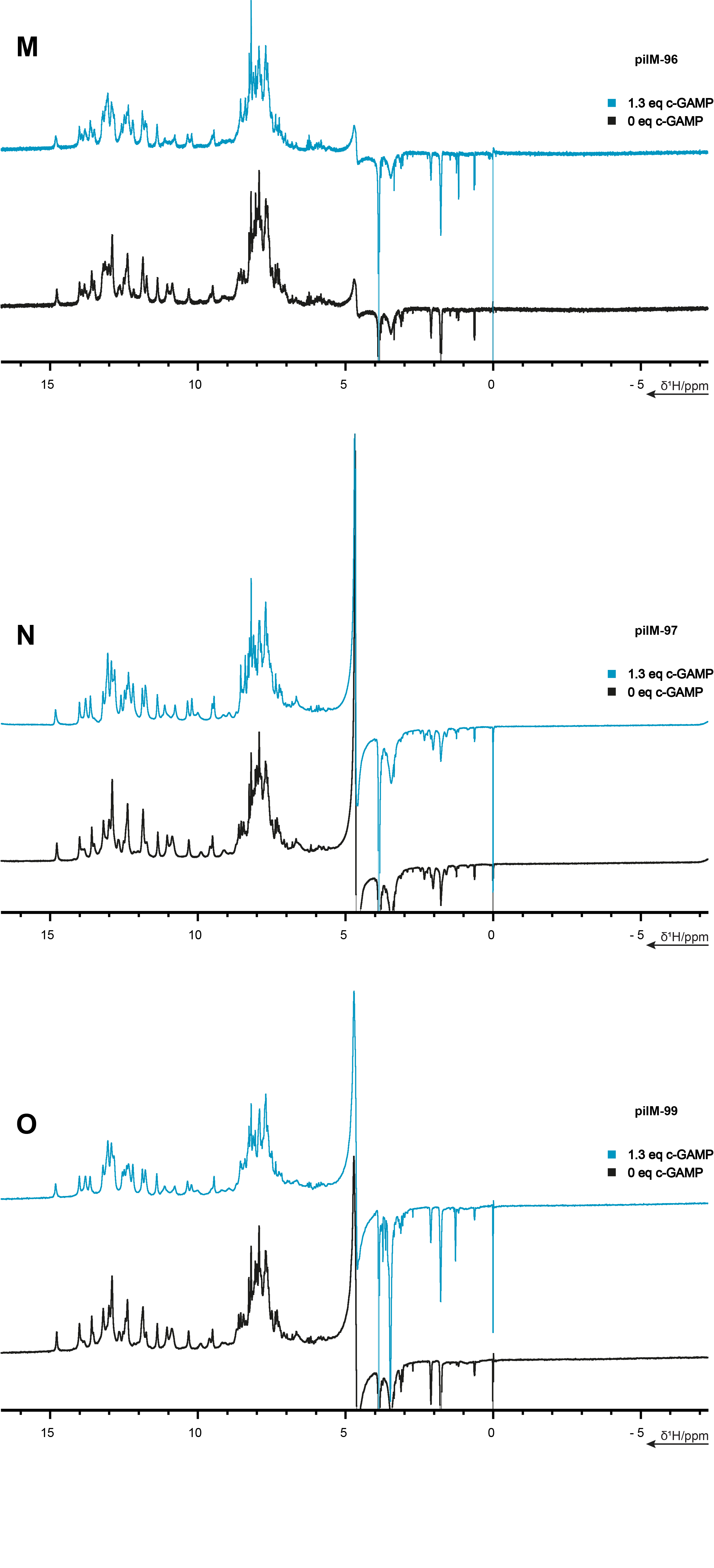

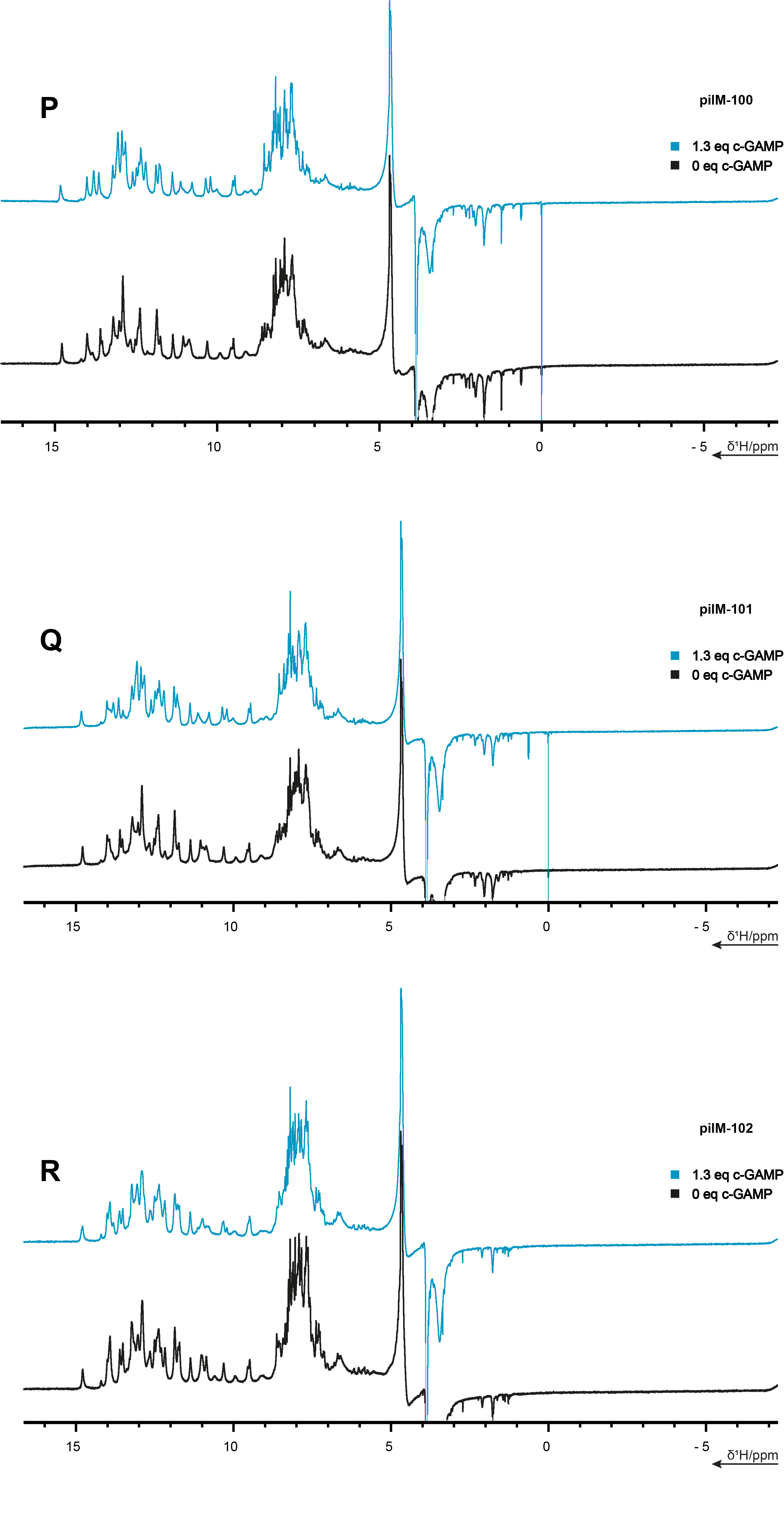


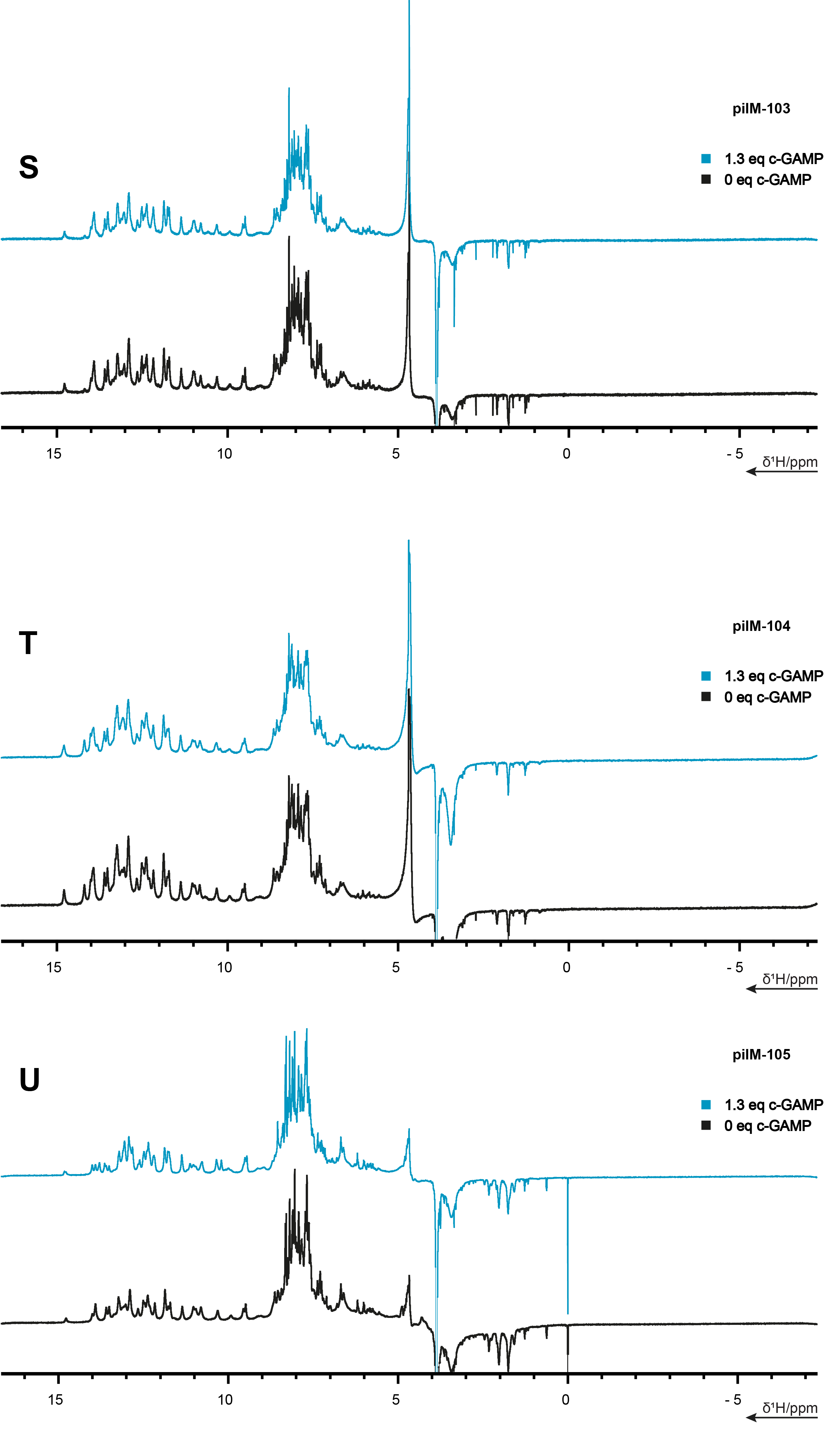


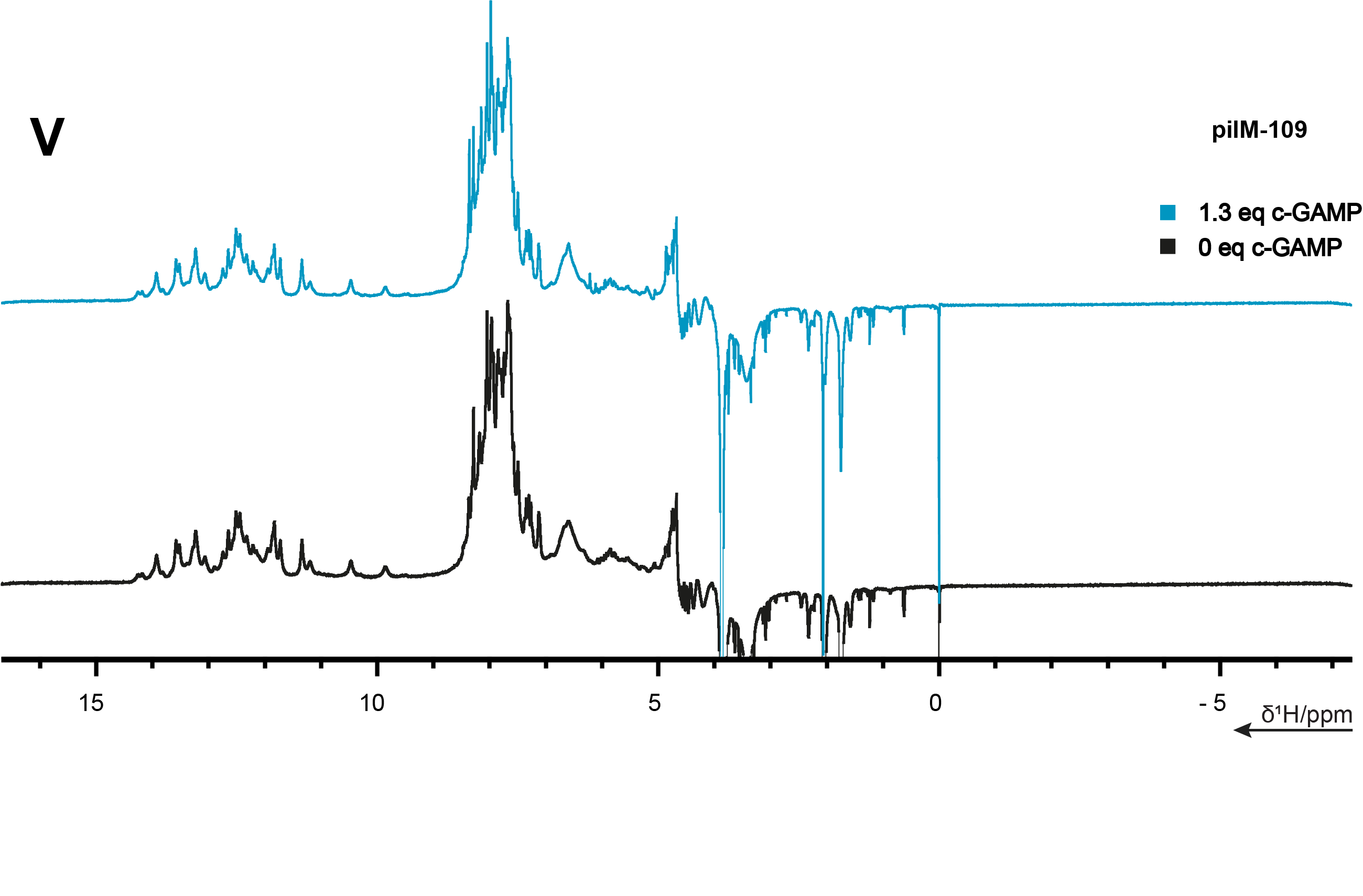


**SI Figure 10: (A)** ^1^H-1D jump-and-return echo NMR spectra of pilM^75^ at a concentration of 200 μM at 308K. The sample also contained 50 mM Bis-Tris Buffer, 50 mM KCl and 5mM MgCl_2_. The spectra were recorded with 32k Time domain points, 1024 scans and 24.5 ppm spectral width at 600 MHz**. (B)** ^1^H-1D jump-and-return echo NMR spectra of pilM^77^ at a concentration of 75 μM at 308K. The sample also contained 50 mM Bis-Tris Buffer, 50 mM KCl and 5mM MgCl_2_. The spectra were recorded with 32k Time domain points, 2048 scans and 24.5 ppm spectral width at 600 MHz**. (C)** ^1^H-1D jump-and-return echo NMR spectra of pilM^79^ at a concentration of 200 μM at 308K. The sample also contained 50 mM Bis-Tris Buffer, 50 mM KCl and 5mM MgCl_2_. The spectra were recorded 4k Time domain points, 512 scans and 24.5 ppm spectral width at 600 MHz**. (D)** ^1^H-1D jump-and-return echo NMR spectra of pilM^80^ at a concentration of 200 μM at 308K. The sample also contained 50 mM Bis-Tris Buffer, 50 mM KCl and 5mM MgCl_2_. The spectra were recorded with 4k Time domain points, 512 scans and 24.5 ppm spectral width at 600 MHz**. (E)** ^1^H-1D jump-and-return echo NMR spectra of pilM^81^ at a concentration of 200 μM at 308K. The sample also contained 50 mM Bis-Tris Buffer, 50 mM KCl and 5mM MgCl_2_. The spectra were recorded with 4k Time domain points, 512 scans and 24.5 ppm spectral width at 600 MHz**. (F)** ^1^H-1D jump-and-return echo NMR spectra of pilM^82^ at a concentration of 200 μM at 308K. The sample also contained 50 mM Bis-Tris Buffer, 50 mM KCl and 5mM MgCl_2_. The spectra were recorded with 4k Time domain points, 512 scans and 24.5 ppm spectral width at 600 MHz**. (G)** ^1^H-1D jump-and-return echo NMR spectra of pilM^83^ at a concentration of 200 μM at 308K. The sample also contained 50 mM Bis-Tris Buffer, 50 mM KCl and 5mM MgCl_2_. The spectra were recorded with 4k Time domain points, 512 scans and 24.5 ppm spectral width at 600 MHz**. (H)** ^1^H-1D jump-and-return echo NMR spectra of pilM^84^ at a concentration of 100 μM at 308K. The sample also contained 50 mM Bis-Tris Buffer, 50 mM KCl and 5mM MgCl_2_. The spectra were recorded with 65k Time domain points, 256 scans and 25 ppm spectral width at 600 MHz**. (I)** ^1^H-1D jump-and-return echo NMR spectra of pilM^88^ at a concentration of 200 μM at 308K. The sample also contained 50 mM Bis-Tris Buffer, 50 mM KCl and 5mM MgCl_2_. The spectra were recorded with 8k Time domain points, 1024 scans and 24 ppm spectral width at 800 MHz**. (J)** ^1^H-1D jump-and-return echo NMR spectra of pilM^91^ at a concentration of 200 μM at 308K. The sample also contained 50 mM Bis-Tris Buffer, 50 mM KCl and 5mM MgCl_2_. The spectra were recorded with 8k Time domain points, 1024 scans and 24 ppm spectral width at 600 MHz**. (K)** ^1^H-1D jump-and-return echo NMR spectra of pilM^93^ at a concentration of 200 μM at 308K. The sample also contained 50 mM Bis-Tris Buffer, 50 mM KCl and 5mM MgCl_2_. The spectra were recorded with 8k Time domain points, 1024 scans and 24 ppm spectral width at 600 MHz**. (L)** ^1^H-1D jump-and-return echo NMR spectra of pilM^95^ at a concentration of 200 μM at 308K. The sample also contained 50 mM Bis-Tris Buffer, 50 mM KCl and 5mM MgCl_2_. The spectra were recorded with 32k Time domain points, 1024 scans and 24.5 ppm spectral width at 600 MHz**. (M)** ^1^H-1D jump-and-return echo NMR spectra of pilM^96^ at a concentration of 200 μM at 308K. The sample also contained 50 mM Bis-Tris Buffer, 50 mM KCl and 5mM MgCl_2_. The spectra were recorded with 32k Time domain points, 1024 scans and 24.5 ppm spectral width at 600 MHz**. (N)** ^1^H-1D jump-and-return echo NMR spectra of pilM^97^ at a concentration of 200 μM at 308K. The sample also contained 50 mM Bis-Tris Buffer, 50 mM KCl and 5mM MgCl_2_. The spectra were recorded with 8k Time domain points, 1024 scans and 24 ppm spectral width at 600 MHz**. (O)** ^1^H-1D jump-and-return echo NMR spectra of pilM^99^ at a concentration of 200 μM at 308K. The sample also contained 50 mM Bis-Tris Buffer, 50 mM KCl and 5mM MgCl_2_. The spectra were recorded with 4k Time domain points, 512 scans and 24.5 ppm spectral width at 600 MHz**. (P)** ^1^H-1D jump-and-return echo NMR spectra of pilM^100^ at a concentration of 200 μM at 308K. The sample also contained 50 mM Bis-Tris Buffer, 50 mM KCl and 5mM MgCl_2_. The spectra were recorded with 8k Time domain points, 512 scans and 24 ppm spectral width at 600 MHz**. (Q)** ^1^H-1D jump-and-return echo NMR spectra of pilM^101^ at a concentration of 200 μM at 308K. The sample also contained 50 mM Bis-Tris Buffer, 50 mM KCl and 5mM MgCl_2_. The spectra were recorded with 8k Time domain points, 512 scans and 24 ppm spectral width at 600 MHz**. (R)** ^1^H-1D jump-and-return echo NMR spectra of pilM^102^ at a concentration of 200 μM at 308K. The sample also contained 50 mM Bis-Tris Buffer, 50 mM KCl and 5mM MgCl_2_. The spectra were recorded with 8k Time domain points, 512 scans and 24 ppm spectral width at 600 MHz**. (S)** ^1^H-1D jump-and-return echo NMR spectra of pilM^103^ at a concentration of 200 μM at 308K. The sample also contained 50 mM Bis-Tris Buffer, 50 mM KCl and 5mM MgCl_2_. The spectra were recorded with 32k Time domain points, 512 scans and 24 ppm spectral width at 950 MHz**. (T)** ^1^H-1D jump-and-return echo NMR spectra of pilM^104^ at a concentration of 200 μM at 308K. The sample also contained 50 mM Bis-Tris Buffer, 50 mM KCl and 5mM MgCl_2_. The spectra were recorded with 8k Time domain points, 512 scans and 24 ppm spectral width at 600 MHz**. (U)** ^1^H-1D jump-and-return echo NMR spectra of pilM^105^ at a concentration of 200 μM at 308K. The sample also contained 50 mM Bis-Tris Buffer, 50 mM KCl and 5mM MgCl_2_. The spectra were recorded with 8k Time domain points, 1024 scans and 24 ppm spectral width at 800 MHz**. (V)** ^1^H-1D jump-and-return echo NMR spectra of pilM^109^ at a concentration of 200 μM at 308K. The sample also contained 50 mM Bis-Tris Buffer, 50 mM KCl and 5mM MgCl_2_. The spectra were recorded with 8k Time domain points, 1024 scans and 24 ppm spectral width at 800 MHz**.**





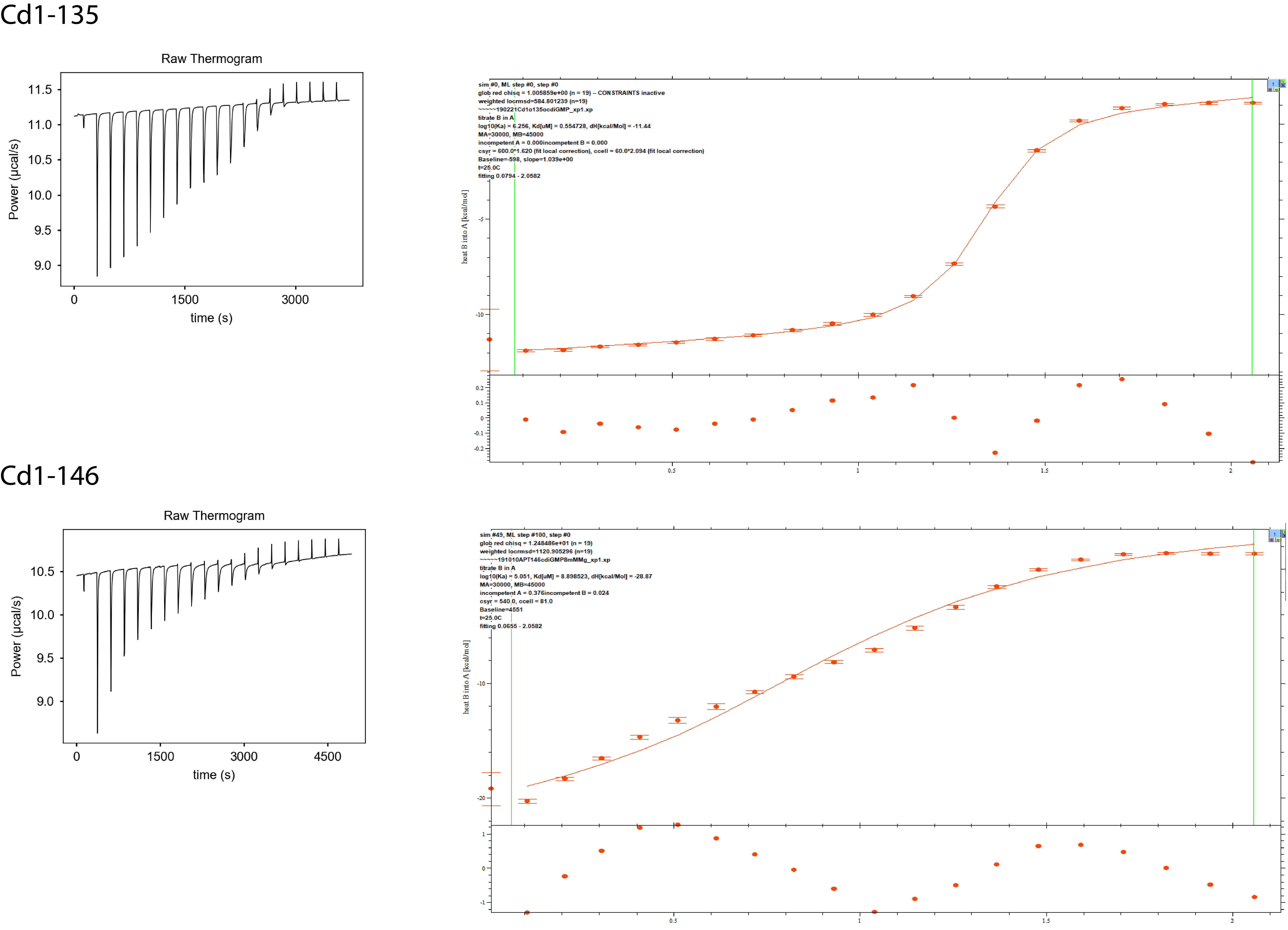


SI Figure 11: ITC raw data for the Cd1 riboswitch: the Raw Thermogram and the original Fit in Sedphat are shown. ITC were measured in 50 mM Bis-TRIS buffer with 8 mM MgCl_2_ and 110 mM NaCl. Concentration of the RNA in single messuments was adjusted to be around 60 µM with the sole exception being 146 with a concentration of 81 µM.


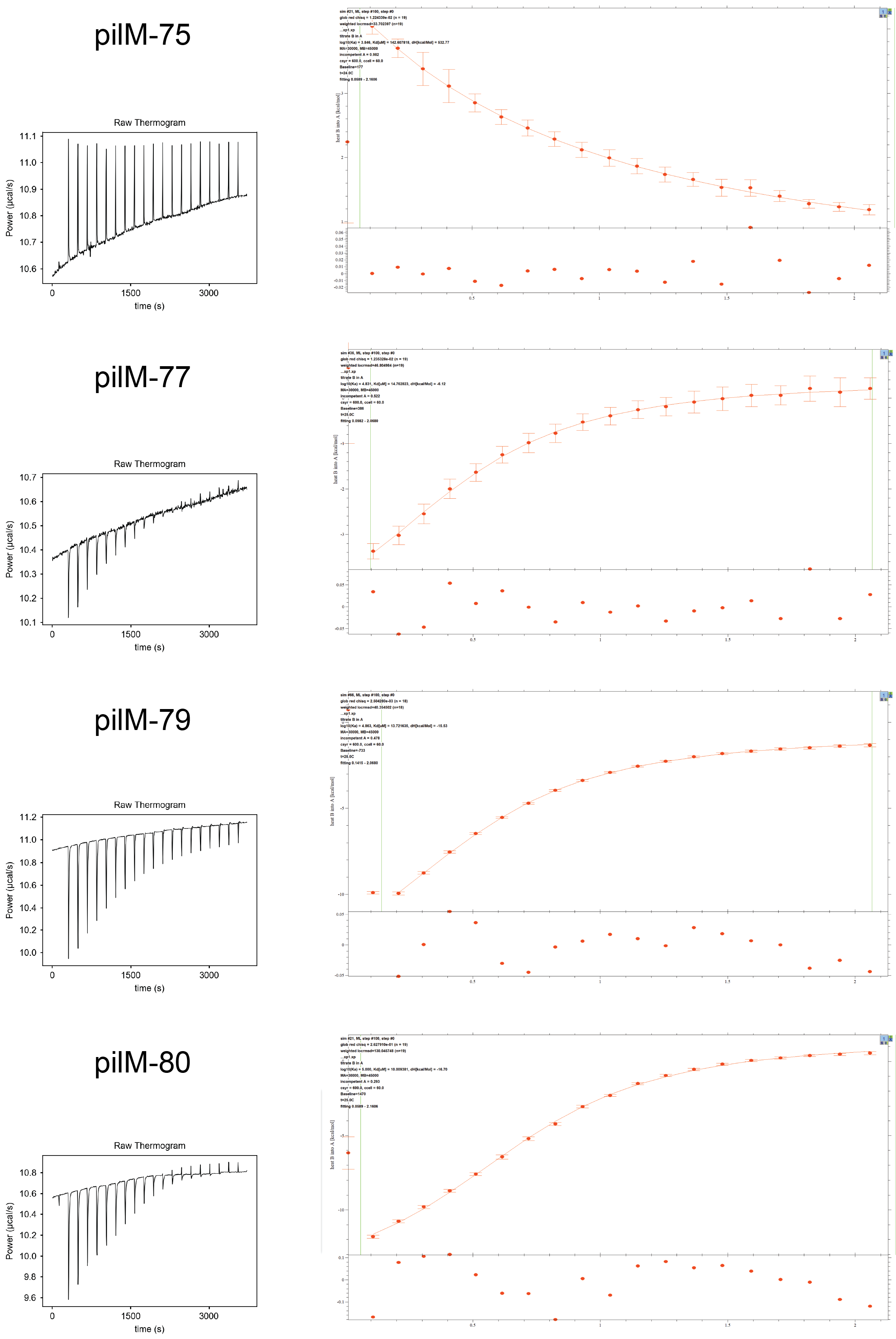


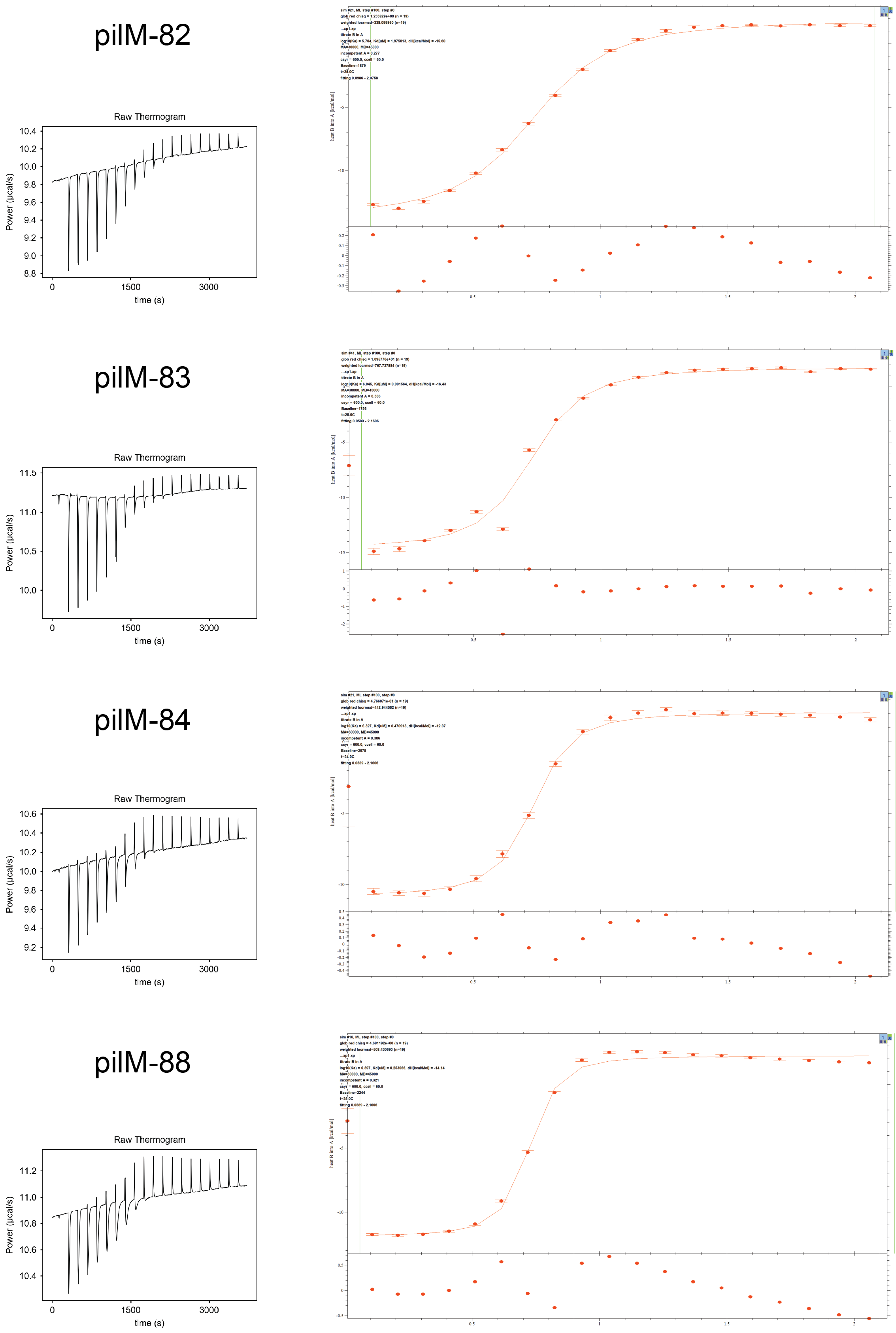

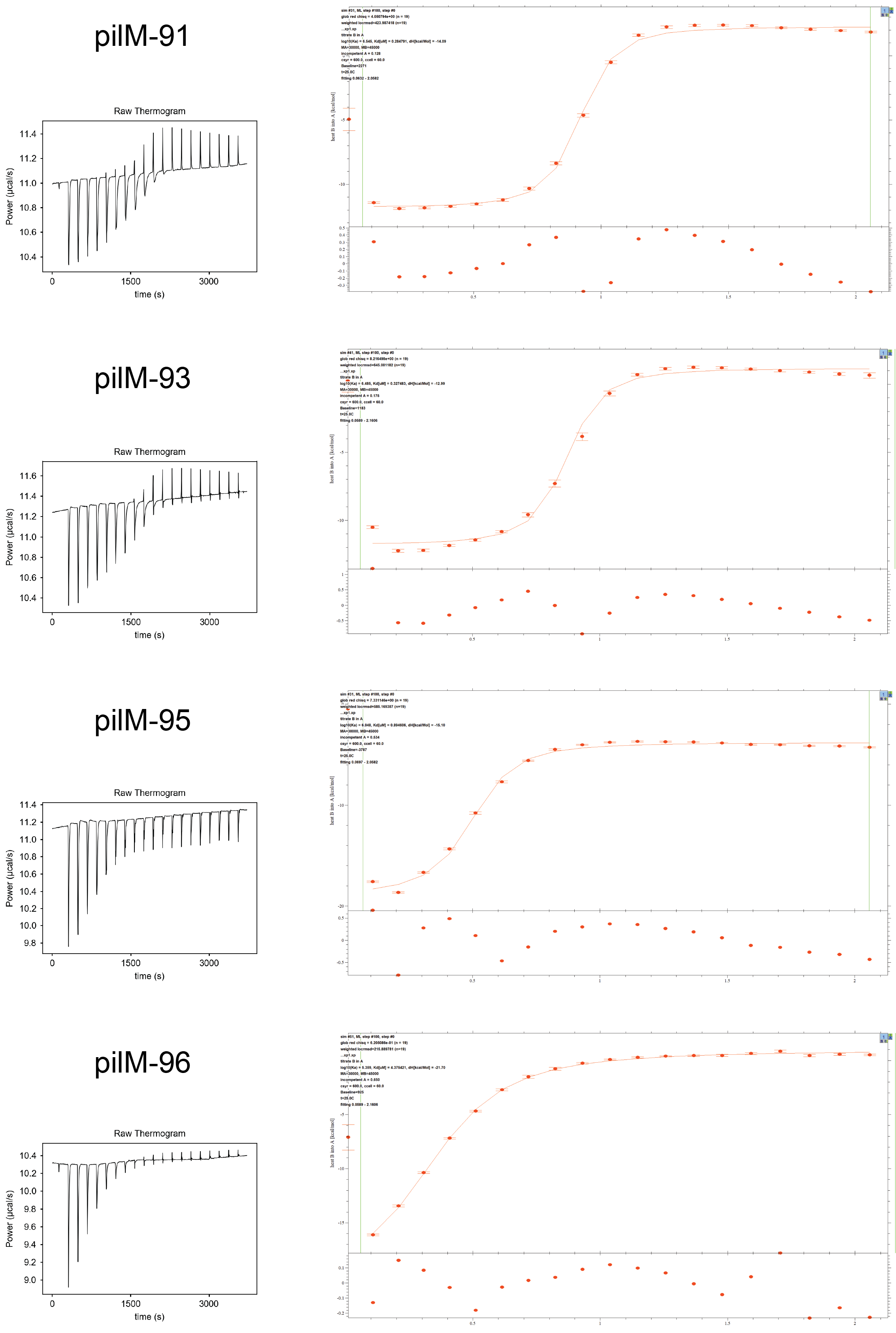

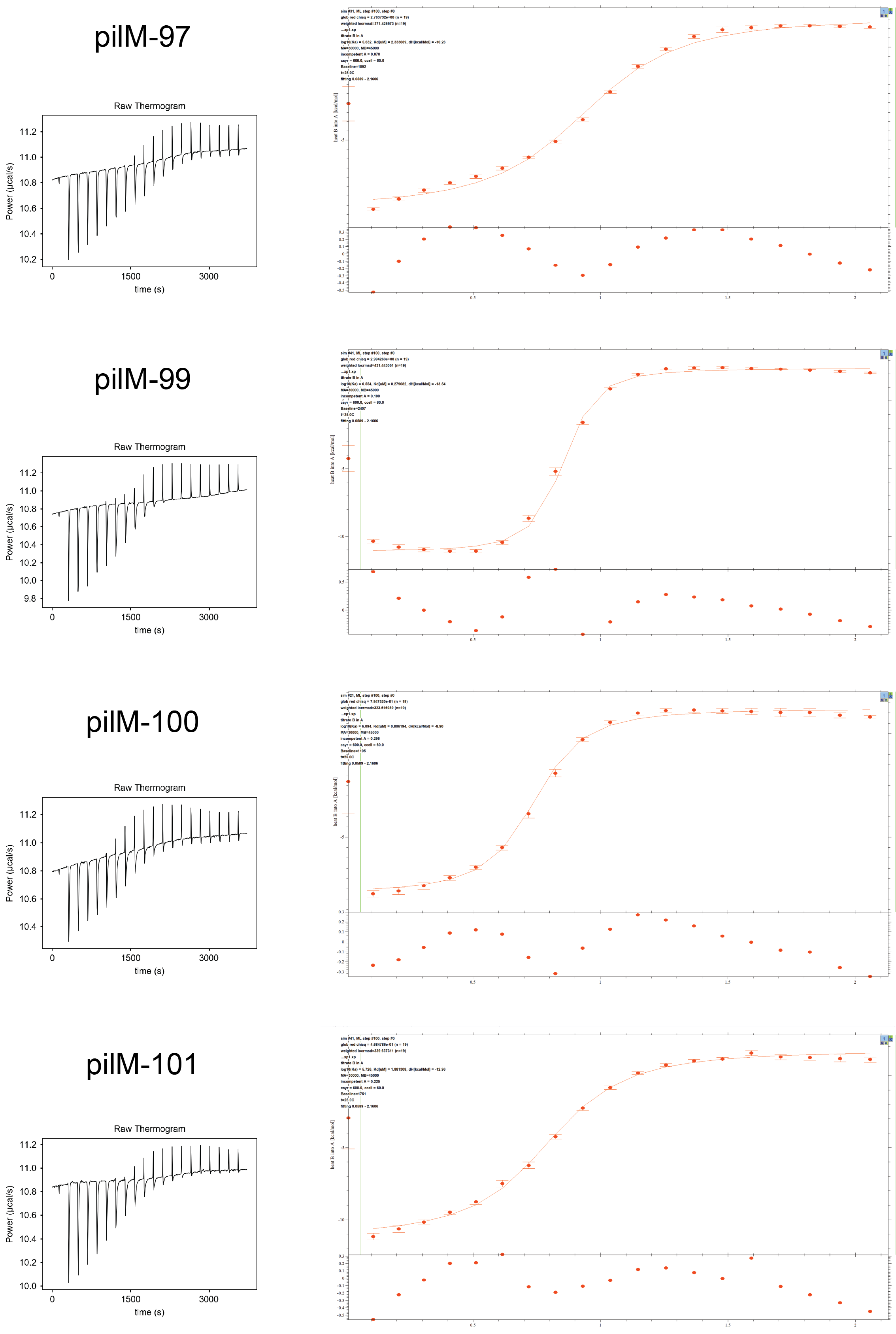

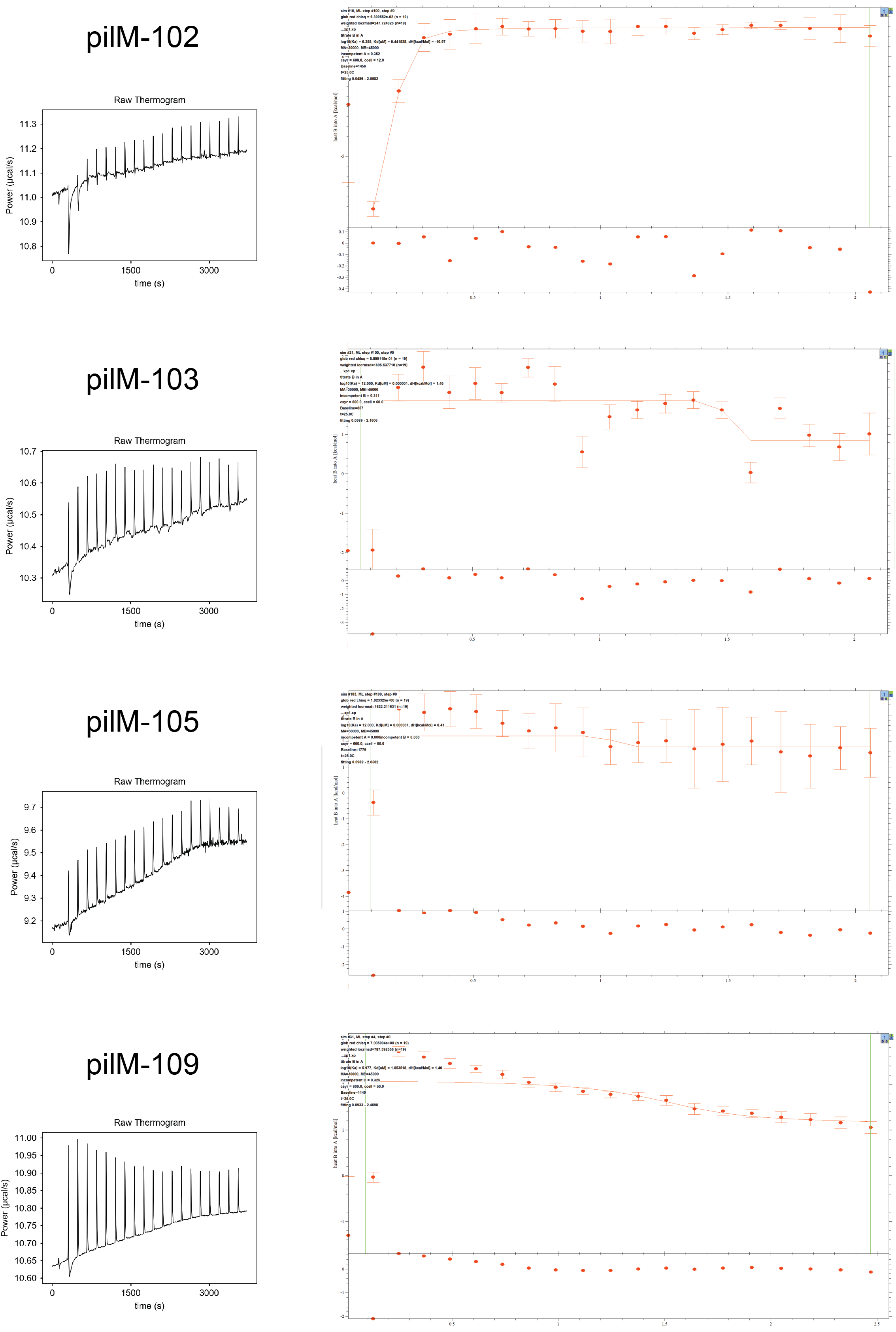


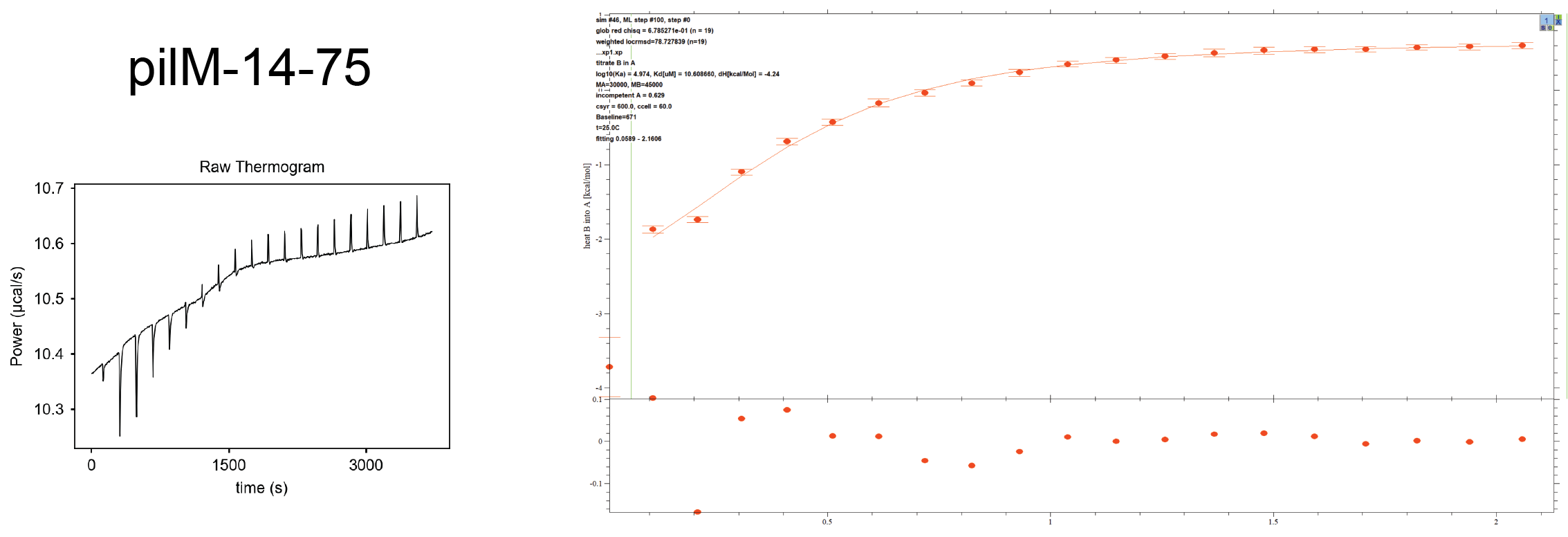


SI Figure 12: ITC raw data for the pilM riboswitch: the Raw Thermogram and the original Fit in Sedphat are shown. The pilM-109 measurement was carried out at 50 µM RNA concentration all others were conduced at 60 µM RNA concentration. The sample also contained 50 mM Bis-Tris Buffer, 50 mM KCl and 5mM MgCl_2._


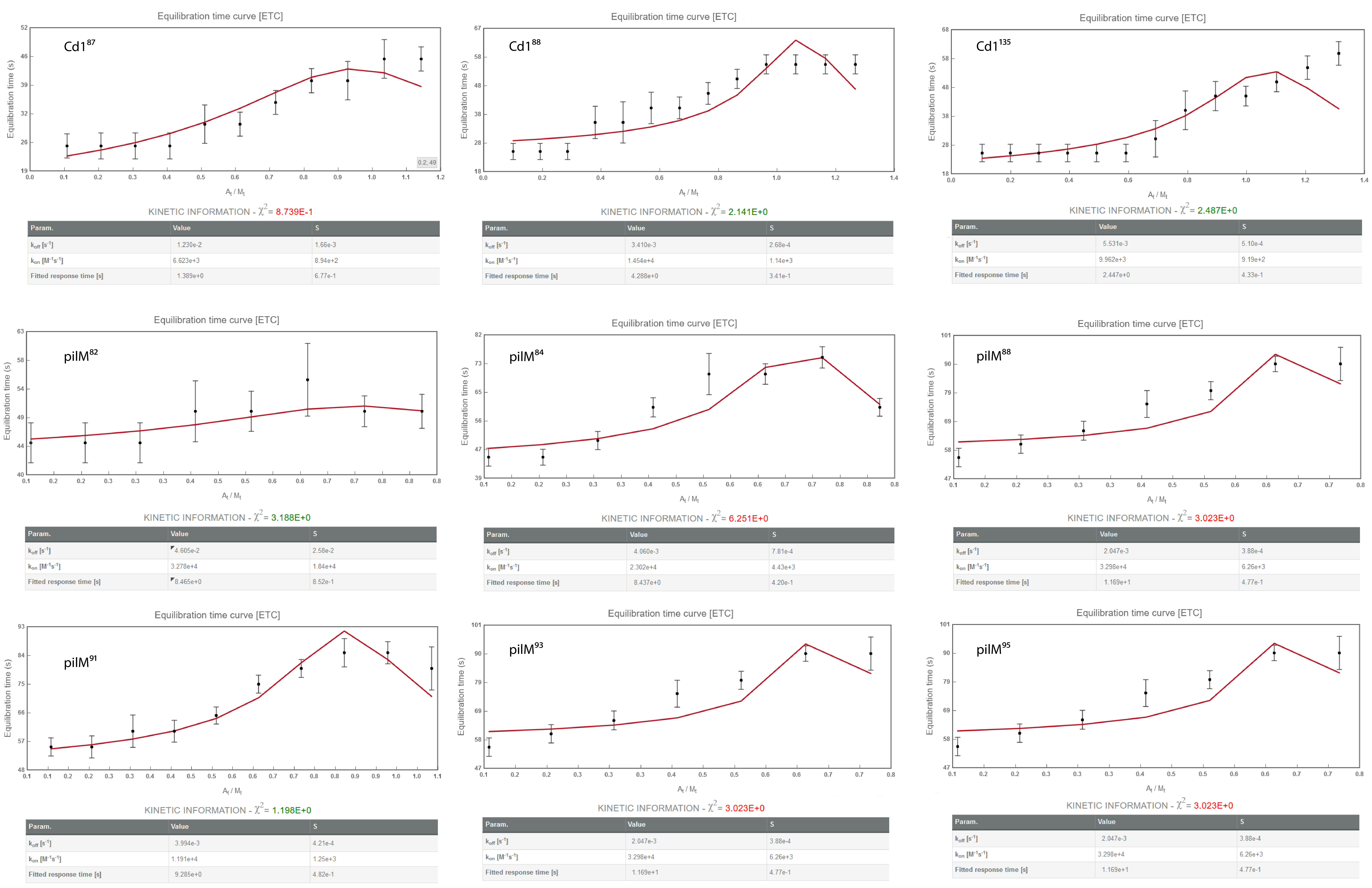


SI Figure 13: Kinetic information obtained for strongly binding pilM and Cd1 riboswitch Constructs using kinITC

**
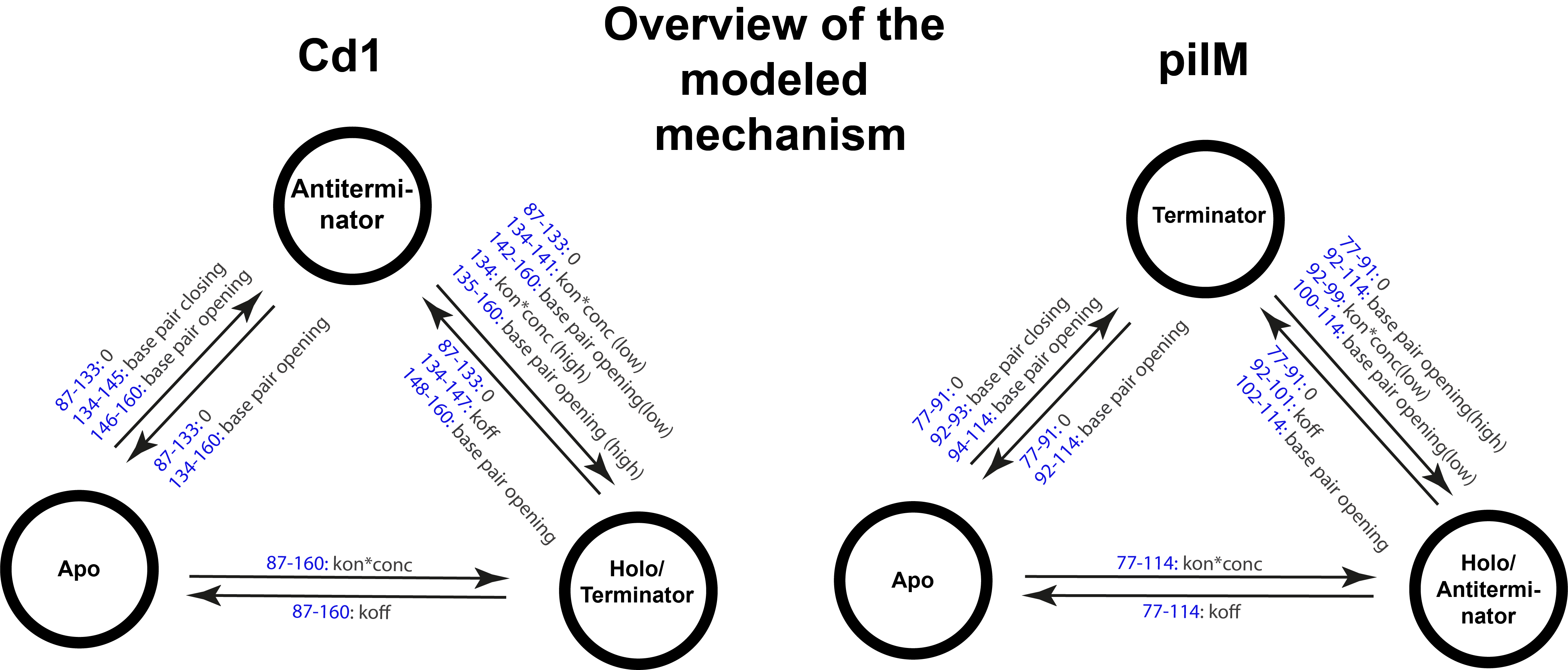
**

**SI Figure 14:** Graphical description of the three state model used in the Markov modeling. The blue numbers refer to the RNA length ranges for which the indicated rates were applied. Low and high designate which rate was used for low or for high ligand concentration simulations (100 µM to 100 nM), respectively.


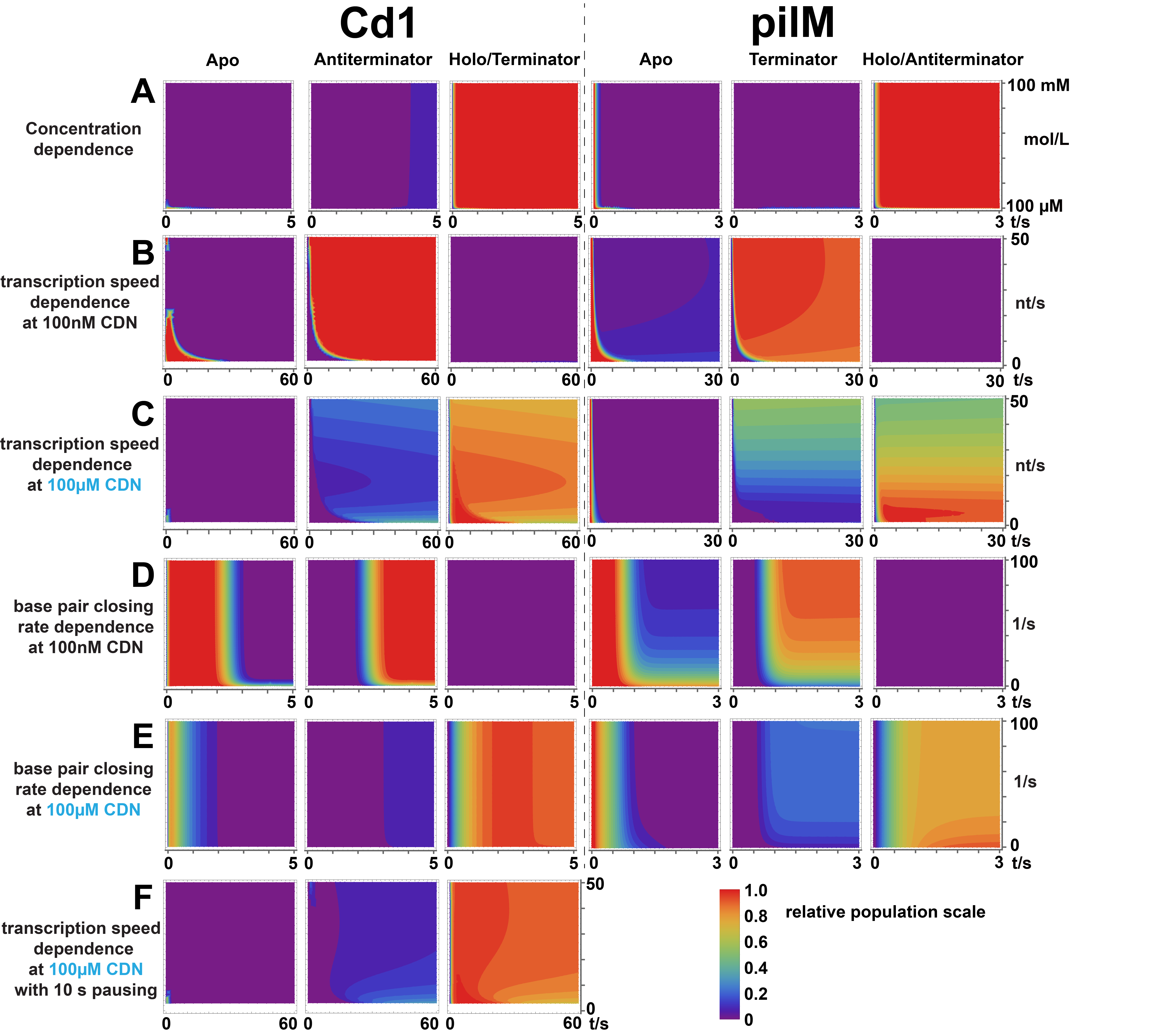


SI Figure 15: Contour level plots of cotranscriptional folding states for the Cd1 and pilM riboswitch, the population of the three functional relevant states over time is depicted in 5% levels of relative population. The population of the macrostates is the sum of all contributing RNA lengths that are in the corresponding state. The conditions of the corresponding simulation were 20nt/s transcription speed, no pausing, 400/s base pair closing rate and either 100nM or 100µM cyclic-di-nucleotide (CDN), c-di-GMP for Cd1 and c-GAMP for pilM. The second variables in the contour level plots were the concentration of CDN (A), the transcription speed at low ligand concentration (B), the transcription speed at high ligand concentration (simulated from 2 to 50 nt/s) (C), the base pair closing rate at low ligand concentration (simulated from 2 to 100 nt/s) (D), the base pair closing rate at high ligand concentration (E) and the transcription speed at high ligand concentration with 10 s pausing at the possible pause site at nucleotides 141-145 (simulated from 3 to 50 nt/s) (F).
